# Divergent patterns of cranial suture fusion in marsupial and placental mammals

**DOI:** 10.1093/zoolinnean/zlae060

**Published:** 2024-05-24

**Authors:** Heather E. White, Abigail S. Tucker, Anjali Goswami

**Affiliations:** 1 Centre for Craniofacial and Regenerative Biology, King’s College London, Great Maze Pond, London SE1 9RT, United Kingdom; 2 Science Department, Natural History Museum, Cromwell Road, London SW7 5BD, United Kingdom; 3 Division of Biosciences, University College London, Gower Street, London WC1E 6DE, United Kingdom

**Keywords:** mammal, marsupial, suture, fusion, Krogman

## Abstract

Cranial sutures, both open and closed, support a myriad of skull functions, including redistributing strain, accommodating brain expansion, supporting cranial bone growth, and protecting the brain. Thus, variation in the degree, timing, and pattern of suture fusion has functional implications. Using a comparative ontogenetic framework across Mammalia, we quantified degree and pattern of suture fusion through ontogeny for 22 mammalian species (*N* = 165). Suture closure was scored on a discrete scale for 31 cranial sutures and used to calculate closure scores for individual sutures and specimens. Ancestral state estimations found the degree of ancestral marsupial fusion to be more derived, differing from both the ancestral placental and ancestral therian. The average placental pattern followed the Krogman pattern of suture fusion (cranial vault, cranial base, circum-meatal, palatal, facial, and cranio-facial), whereas marsupials showed a distinct pattern. We propose a new pattern of suture fusion for marsupials: vault, cranio-facial, facial, circum-meatal, palate, cranial base. Delayed fusion of the marsupial cranial base is hypothesized here to support prolonged postnatal growth of the marsupial brain. Collectively, our study has identified a clear marsupial-placental dichotomy in the degree, timing, and pattern of suture fusion, with implications for understanding skull function and ontogeny.

## Introduction

Cranial suture fusion is not simultaneous, nor does it occur to the same degree in each suture (Sánchez-Villagra 2010); some sutures fuse early during ontogeny, while others never fuse. Both fusion and patency (an open suture) are important to the overall functioning of the skull (White *et al*. 2021). Open sutures primarily function as sites of interstitial bone growth (Opperman 2000) through the action of pluripotent suture mesenchymal stem cells (Zhao *et al*. 2015, Maruyama *et al*. 2016). These suture mesenchymal cells differentiate into osteoblasts to produce bone matrix, enabling the growth, homeostasis, and repair of cranial bones (Lana-Elola *et al*. 2007, Zhao *et al*. 2015). Consequently, suture fusion prevents bone growth and results in the termination of skull development (Goswami *et al*. 2013). Suture fusion status is tightly controlled by an array of signalling factors throughout ontogeny (Rice *et al*. 2000, Lenton *et al*. 2005, Grova *et al*. 2012, Flaherty *et al*. 2016). Perturbations in these signalling pathways cause deviations from a standard pattern and timing of suture fusion and lead to cranial deformities such as craniosynostosis (Morriss-Kay and Wilkie 2005, Flaherty *et al*. 2016). Ontogeny of suture fusion, therefore, plays a crucial role in the development of the cranium.

Suture fusion supports the cranium under excessive biomechanical strain following localized functional demands (Herring 1974, Bärmann and Sánchez-Villagra 2012). For example, a long-standing hypothesis proposes that suture fusion acts to protect the fully developed brain via strengthening surrounding cranial regions. Nevertheless, strain gradients known to be present across the adult mammalian skull suggest that the cranial vault experiences low strain compared to the craniofacial region (Ross and Metzger 2004). Thus, protection of the brain via suture fusion is less likely to be a requirement in mammalian taxa. In addition to this suture fusion, suture patency has also been linked to supporting the brain. Suture patency and brain size are tightly integrated (Richtsmeier and Flaherty 2013) due to the role patent sutures have in supporting cranial bone growth and skull expansion. Suture patency is, therefore, thought to support the growth of the developing brain and thus enabling extreme encephalization in humans (Jeffery and Spoor 2002, Lesciotto and Richtsmeier 2019). However, it has been conversely hypothesized that sutures may play a greater role in shock absorption than supporting brain growth since masticatory forces have a greater impact on the level of suture fusion than brain expansion (Cray *et al*. 2010). The masticatory process is supported by patent sutures in the adult cranium that act to redistribute biomechanical strain across the skull (Rayfield 2005, Moazen *et al*. 2009). Open sutures in the adult cranium, therefore, reduce the impact of highly localized strains arising from a heightened bite force (Moazen *et al*. 2009). Via similar mechanisms, strain redistribution of patent adult sutures can provide shock absorption (Moazen *et al*. 2009) from additional external pressures, such as head-to-head fighting and locomotion. There is, however, conflicting evidence as to the role of suture fusion under biomechanical demands. For example, it has been suggested that premature fusion may instead act to strengthen the skull to with-stand excessive biomechanical strain on a specific cranial region, such as the facial and palate sutures of peccaries (*Pecari*), extinct even-toed ungulates (*Cainotherium*), and bovids (Herring 1974, Bärmann and Sánchez-Villagra 2012). Beyond suture fusion, final adult suture morphology is also known to impact the nature of stress. Interdigitated sutures, a late-developing feature, are associated with compressive stress and strain, whilst butt-ended sutures are linked to tensile stress and strain, and overlapping joints accommodate complex loading (Herring 2008). Nevertheless, it is clear that both suture fusion and patency serve important functional roles in the developed adult specimen, though the relative weighting of these different roles remains ambiguous. This functional importance between fused and patent is particularly relevant during ontogeny as sutures shift from a patent to a fused state (Grova *et al*. 2012, Goswami *et al*. 2013, Flaherty *et al*. 2016). Suture fusion changes consequently have significant implications for skull function.

A high degree of variability has been reported in the timing and location of suture fusion across mammals. Across mammalian infraclasses, placentals have been found to have more closed sutures than marsupials (Rager *et al*. 2014). At the species-level, interspecific variation in suture fusion is observed between closely related species within the same order, including artiodactyls, primates, rodents, and bats (Giannini *et al*. 2006, Cray *et al*. 2008, 2014, Wilson and Sánchez-Villagra 2009, Bärmann and Sánchez-Villagra 2012). Interspecific variation in suture closure is thought to be associated with varying levels of biomechanical stress (Herring 1972, 1974, Bärmann and Sánchez-Villagra 2012, Cray *et al*. 2014). For example, the side-to-side movements of ruminants impose greater shearing forces on the skull than the up–down biting action of carnivores. This has been proposed to lead to an overall lower degree of sutural fusion in ruminants (Bärmann and Sánchez-Villagra 2012). Variation is also evident within a species. For example, sexual dimorphism in suture fusion has been reported in rhesus monkeys (*Macaca mulatta*) (Wang *et al*. 2006) but not in water deer (*Hydropotes inermis*) (Oh *et al*. 2017), although research regarding sexual dimorphism is limited.

Variation in the degree of suture fusion is also evident across the skull of an individual, where not all sutures within the cranium of a single species fuse. In the mouse and rat, for example, only the interfrontal suture is reported to fuse (Bradley *et al*. 1996, Opperman 2000). In many other species of placental mammal (e.g. rhesus monkey, giraffe, several species of deer, Eurasian beaver, lowland paca, red-rumped agouti, capybara, southern elephant seal, and black-capped squirrel monkey), several sutures have been identified to remain completely patent in the adult stages (Wilson and Sánchez-Villagra 2009, Bärmann and Sánchez-Villagra 2012, Flores and Barone 2012, Goswami *et al*. 2013, Oh *et al*. 2017).

In addition to differences in the degree and location of fusion, the direction of fusion is also thought to be highly variable, and reported to start both ectocranially or endocranially (Todd and Lyon 1924, Krogman 1930, Sun *et al*. 2004). Studies focusing on humans and primates (Todd and Lyon 1924, Krogman 1930) identify initial fusion at the endocranial surface, whereas in pigs (*Sus scrofa*) fusion has been reported to occur ectocranially first (Sun *et al*. 2004). Nevertheless, due to the ease of study, most research focuses on the ectocranial surface (Wilson and Sánchez-Villagra 2009, Bärmann and Sánchez-Villagra 2012, Goswami *et al*. 2013, Oh *et al*. 2017).

An order of suture closure has long been defined in hominids (Krogman 1930), widely referred to as the Krogman pattern: cranial vault (first to fuse), cranial base, circum-meatal, palatal, facial, and cranio-facial (last to fuse). More recently an alternative hypothesis has been proposed, suggesting that fusion occurs in an anteroposterior direction across Mammalia. Analysis of this antero-posterior fusion pattern was, however, only performed for three sutures (metopic, coronal, and sagittal) compared to the entire skull of Krogman (1930). Since the proposal of the Krogman pattern, comparative studies have identified both similarity and dissimilarity to this pattern in a range of mammalian species. Specifically, the Krogman pattern has been corroborated in Old and New World monkeys, (Schweikher 1930, Chopra 1957), and in several, but not all, species of Carnivora and hystricognath rodents (Wilson and Sánchez-Villagra 2009, Goswami *et al*. 2013). Identification of the Krogman pattern in these non-hominid taxa thus suggests that the Krogman pattern may be common to Mammalia. However, peccaries (*Pecari*), megabats (*Pteropus*), and some species of Carnivora and hystricognath rodents appear to deviate from the Krogman pattern of suture fusion (Herring 1972, 1974, Giannini *et al*. 2006, Wilson and Sánchez-Villagra 2009, Goswami *et al*. 2013), making it unclear whether the suture fusion pattern is species-specific or more conserved across Mammalia.

One element of suture fusion that is better established as a conserved pattern across mammalian species is the early fusion of sutures surrounding the foramen magnum (Bärmann and Sánchez-Villagra 2012). This conserved pattern is observed in diverse taxa, including artiodactyls (Bärmann and Sánchez-Villagra 2012), rodents (Wilson and Sánchez-Villagra 2009), primates (Wang *et al*. 2006), megabats (Giannini *et al*. 2006), and seals (Brunner *et al*. 2004), with the Florida manatee (*Trichechus manatus latirostris*) being identified as an exception (Hoson *et al*. 2009). Further deviations from this pattern are observed in species of primates (*Saimiri sciureus* and *Saguinus nigricollis*, and *Pan troglodytes* and *Gorilla gorilla*) (Dolan 1971, Cray *et al*. 2008), despite primates forming the focal group for the Krogman pattern (Krogman 1930). Both similarities in pattern and deviations from relatively conserved patterns are observed in closely related species and thus have been interpreted as evidence that suture closure patterns may closely reflect phylogenetic relatedness (Bärmann and Sánchez-Villagra 2012, Cray *et al*. 2014, Oh *et al*. 2017).

The study of suture fusion patterns has been revolutionized by the implementation of developmental sequence-based analysis (Smith 2001a, 2008), which in turn has allowed for increased sampling of diverse species, despite the absence of well-staged developmental data in museum collections. Whilst the methods proposed by Smith (2001a) have driven an increase in studies exploring suture fusion, such studies often lack ontogenetic data and, as a result, offer limited resolution of fusion patterns. Moreover, many of these recent studies are limited to analyses of relatively small clades. Overwhelmingly primates have formed the main clade of focus for suture fusion analysis (Krogman 1930, Chopra 1957, Dolan 1971, Wang *et al*. 2006, Cray *et al*. 2008, 2014, Flores and Barone 2012), although other studied clades include artiodactyls (Herring 1974, Sánchez-Villagra 2010, Bärmann and Sánchez-Villagra 2012, Oh *et al*. 2017), carnivorans, including feliforms, caniformes, and pinnipeds (Schweikher 1930, Brunner *et al*. 2004, Goswami *et al*. 2013), hystricognath rodents (Wilson and Sánchez-Villagra 2009), megabats (Giannini *et al*. 2006), and one afrotherian taxon, the Florida manatee (*Trichechus manatus latirostris*) (Hoson *et al*. 2009). In contrast, studies of suture fusion across a broad range of mammalian species are limited. Rager *et al*. (2014) analysed the most comprehensive dataset to date, as well as being the first study to incorporate xenarthrans and marsupials. However, its omission of ontogeny limits our ability to determine exact suture fusion timings and the developmental influence on the evolution of suture fusion. Given that developmental traits underpin evolutionary transformations, comparative ontogenetic frameworks are thought to be particularly important when studying suture fusion timings and patterns (Goswami *et al*. 2013).

Here, we expand on previous studies of suture fusion by implementing the most detailed approach of suture closure assessment to date, and by incorporating rarely studied species of Monotremata, Marsupialia, Xenarthra, and Afrotheria into a large comparative ontogenetic dataset spanning the phylogenetic breadth of Mammalia. Most previous studies perform ectocranial analysis of suture fusion, although variation in patterns of fusion is also known to occur endocranially. Therefore, in addition to ectocranial suture fusion analysis we also quantified cross-sectional suture fusion. Cross-sectional analysis was able to provide confirmation of ectocranial results or highlight the need for studying suture fusion in three-dimensions. Thus, data collection for both ectocranial and cross-sectional analysis simultaneously is pertinent to determining the need for cross-sectional analysis in future studies. In our study, from the ectocranial analysis, we quantify developmental patterns of suture fusion (degree, timing, and pattern) in a macroevolutionary frame-work. Specifically, we aim to (i) determine the appropriateness of using suture fusion as a proxy for developmental age; (ii) identify whether a conserved pattern of suture fusion is observed across Mammalia; (iii) test the applicability of the Krogman pattern to a non-primate only dataset; and (iv) quantify whether the different developmental modes of marsupials and placentals affect the degree of suture fusion or pattern of suture closure. Our work, therefore, builds on the available literature to produce a clearer picture of the evolution of mammalian suture fusion from a developmental and macroevolutionary perspective.

## Materials and Methods

### Specimens and scans

A comparative ontogenetic framework was compiled, consisting of 165 specimens for 22 mammalian species (as described in: White *et al*. 2023a) (total: *N* = 165; adults: *N* = 22). For each species, 4–13 specimens were sampled, in most cases ranging from foetal to adult developmental stages. Species spanned the phylogenetic breadth of Mammalia, to include representatives of the four major placental superorders (Xenarthra, Afrotheria, Laurasiatheria, and Euarchontoglires), three diverse marsupial orders (Didelphimorphia, Dasyuromorphia, and Diprotodontia), and Monotremata. The dataset was comprised of specimens from global museum collections (Natural History Musum, London, NHMUK; Texas Memorial Museum, TMM; South Australian Museum, SAM; Zoologisches Museum Berlin, ZMB; Muséum National d’Histoire Naturelle, MNHN; University Museum of Zoology in Cambridge, UMZC; University Museum of University of Tokyo, UMUT; Duke Lemur Centre, DLC;). Full specimen details can be found in the Supporting Information, Tables S1 and S2.

All specimens were imaged using X-ray micro-computed tomography (CT) (details of specific instruments in Supporting Information, Tables S2, S3). Three-dimensional CT models were reconstructed using AVIZO v.9.3 (FEI, OR, USA) to produce 3D surface meshes, and decimated to reduce the number of faces in MESHLAB v.2020.7 (Cignoni *et al*. 2008). Meshes were cleaned in Geomagic Wrap (3D Systems) to remove artefacts and unwanted bones (e.g. vertebrae and mandible). The left-hand side of the skull was used for data collection; therefore, specimens with damage to the left side but intact right sides were mirrored in Geomagic Wrap (3D Systems) before fusion data was collected (Supporting Information, Table S4).

### Ontogenetic age

Specimen age was estimated using both a continuous and discrete approach (Supporting Information, Table S5). The continuous approach used centroid size, which was determined from 69 Type I and II landmarks that were positioned using Stratovan Checkpoint (Stratovan Corporation, CA, USA) and subjected to generalized Procrustes’ analysis, as described in White *et al*. (2023a) and detailed in the Supporting Information, Table S6. To correct for differences in overall body size, we calculated CS (centroid size) for each specimen as a percentage of the adult size for their species, providing a continuous proxy for developmental age (Supporting Information, Table S5). It is important to note here that variation in the rate of development based on the altricial–precocial spectrum impacts continuous age, with more developed precocial taxa displaying a greater percentage of adult centroid size than their altricial counterparts. For greater details see figure 4B in White *et al*. (2023a).

We also grouped specimens into four discrete age categories, consisting of foetal (F), infant (I), juvenile (J), and adult (A) stages (Supporting Information, Table S5), as outlined in White *et al*. (2023a). Museum specimen information was used to first subdivide specimens into foetal (F), infant (I), and adult (A) categories. As the infant age category was large, we secondarily subdivided this into two categories: infant and juvenile. The juvenile category was determined using the percentage of adult CS, described above, and defined as the second largest specimen for each species. Where species had only one infant specimen, no juvenile was assigned to this species. In select cases, multiple specimens for an individual species presented with a high percentage of adult CS; thus, when more than one specimen for a species presented with >95% for the percentage of adult CS, all such specimens were considered juvenile. Marsupials were assigned a ‘foetal’ stage to reflect similar categories assigned to placentals. Any specimen with a known age of <20 days old, and any marsupial specimens with a similar visual level of ossification to those <20 days old, where absolute age data was unavailable, were assigned to the marsupial ‘foetal’ category. This categorization of marsupial ‘foetal’ specimens was determined based on visual assessment of marsupial ossification compared to ossification levels of placental foetal specimens. For the only monotreme within the dataset, the youngest *Ornithorhynchus anatinus* presented with a much higher level of ossification and cranial maturity than the youngest marsupials. Therefore, no foetal stage was assigned for *Ornithorhynchus anatinus*.

### Phylogeny

For the phylogenetic framework, a recent and extensive (5911 mammalian species) time-calibrated maximum clade credibility phylogeny (Upham *et al*. 2019), produced using molecular data and dated with extensive fossil calibrations, was used here. This phylogeny was trimmed using the ‘*keep.tip*’ function in the *ape* R package (Paradis *et al*. 2004) to generate a phylogeny for the 22 species in the dataset (Supporting Information, Fig. S1). We cross-checked our trimmed marsupial topology with the most recently published marsupial phylogeny (Beck *et al*. 2022). Phylogenetically informed analyses for the adult-only specimens were performed using this trimmed phylogeny. However, no phylogenetic analyses were performed for the ontogenetic dataset as multiple developmental stages for the same species cannot be constrained into a single phylogeny (Navalón *et al*. 2021).

### Suture data collection

**Figure 1:**
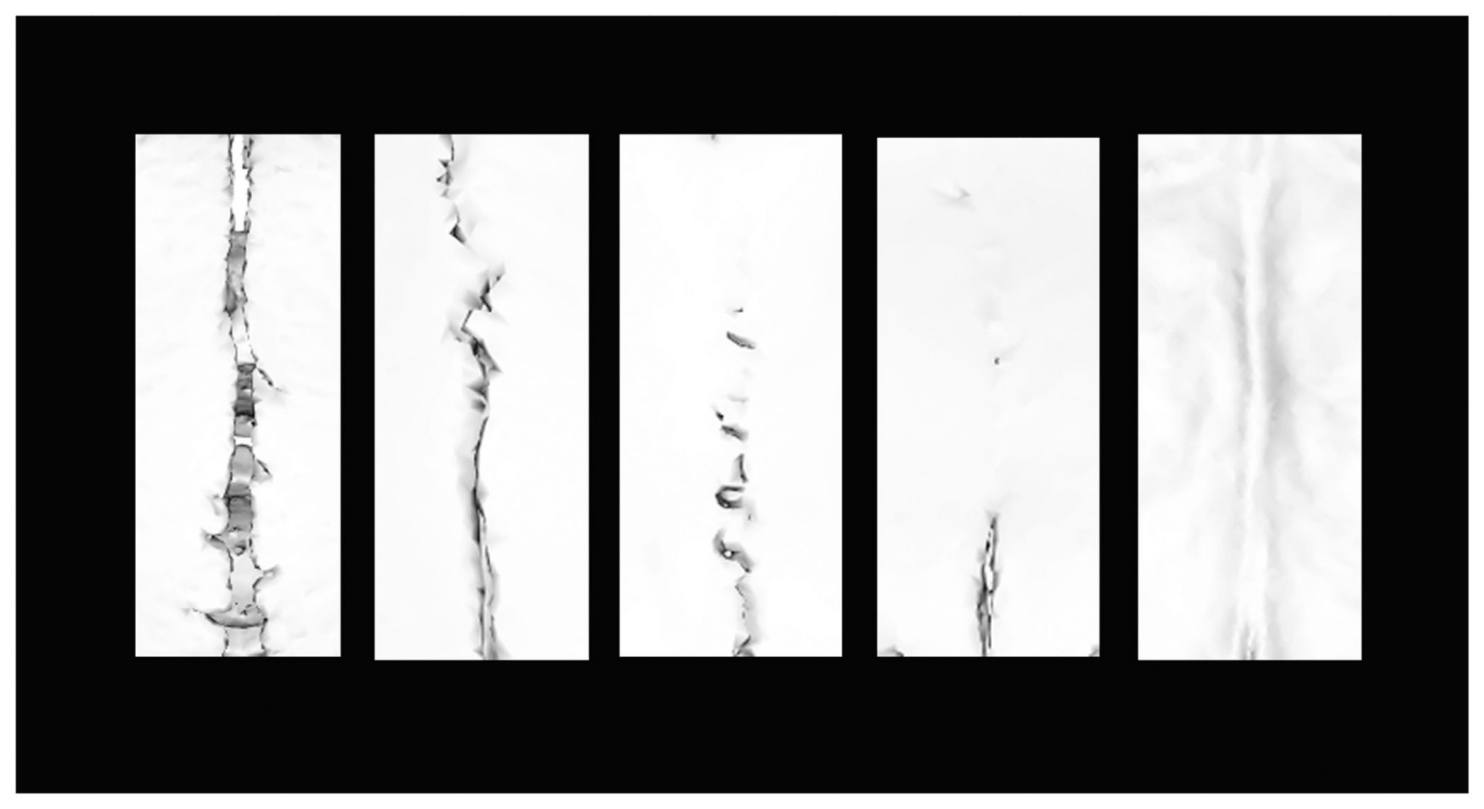
Scoring ectocranial suture fusion, illustrating the varying levels of fusion: fully open (1); quarter fused (2); half fused (3); three-quarter fused (4); entirely fused (5).

Sutures were scored based on the extent of fusion using the re-constructed 3D meshes for the 165 specimens, for 31 sutures and synchondroses covering the cranium (Table 1; Supporting Information, Table S7), collectively referred hereafter as sutures. Each of these sutures was assigned a developmental origin (neural crest, mesoderm, or boundary) based on the origin of the surrounding cranial bones reported within the literature from mouse lineage studies (Supporting Information, Table S8). Suture fusion has often been captured using a binary approach (0,1) (Rager *et al*. 2014), which disregards the possibility of a suture being partially open or partially fused and thus limits the ability to distinguish between open and partially fused sutures or fused and partially open sutures. To provide a more accurate measure of suture fusion, these intermediate states are accommodated by quantifying the degree of suture fusion based on the approach implemented by Wilson and Sánchez-Villagra (2009) and Goswami *et al*. (2013). The degree of suture fusion was scored for the left-hand side of the skull and was based on the observed proportion of closure: fully open (score 1); quarter fused (score 2); half fused (score 3); three-quarter fused (score 4); entirely fused (score 5) (Fig. 1; Supporting Information, Table S7). An entirely fused suture refers to a suture that is fused across its full length (100%). Note, complete fusion of the suture length does not necessarily mean the suture is completely obliterated from view.

Variably present or missing bones resulted in missing data for some sutures. First, variably present bones lead to the absence of certain sutures for some species; these include the maxilla-jugal suture, jugo-squamosal suture, and premaxillo-maxillary ventral suture. When absent, the degree of suture fusion was, therefore, scored as 1 (open). Variably present bones were also common for the youngest foetal specimens, as some cranial bones were not yet established; these sutures were similarly considered as open (1). In several species, the interface between the supraoccipital and parietal was absent due to the presence of the interparietal bone in some species (*Bradypus tridactylus, Cyclopes didactylus, Dasypus novemcinctus, Monodelphis domestica, Ornithorhynchus anatinus, Phacochoerus africanus, Sapajus apella, Setifer setosus, Sminthopsis macroura*, and *Talpa europaea*). For species with an interparietal present, the supraoccipito-parietal suture was scored at the interparietal–parietal interface as the interparietal is known in many species to fuse with the supraoccipital (Koyabu *et al*. 2012). Second, for missing bones, i.e. those that are preservationally damaged or broken off, sutures were initially scored with a ‘?’. Using developmental stages either side of the ‘?’, scores were interpolated. For example, if three specimens in a developmental series in ascending age order scored ‘2’ for the youngest, ‘?’ for the middle stage, and ‘4’ for the oldest, then the middle specimen would be inferred as stage ‘3’.

Finally, as fusion is known to vary along the length and depth of a suture (Krogman 1930, Sun *et al*. 2004), cross-sectional suture fusion was additionally collected from the X-ray projection slices for adult specimens for three sutures (interfrontal, sagittal, and coronal) at three equidistant points along the length of the suture (Supporting Information, Fig. S2). Cross-sectional data collection was captured using CT scan X-ray projection slice images where sutures were scored as either open (0), partially closed (half), or closed (1) (for further details, see Supporting Information, Supplemental Methods; Fig. S3). In contrast, ectocranial scoring was conducted using the reconstructed 3D meshes.

### Calculation of closure scores

Suture closure score (%) was calculated for each suture separately, combining the fusion data for all adult specimens (*N* = 22) and all specimens of the full developmental dataset (*N* = 165). Suture closure score was calculated as the sum of the percentage of quarter fused sutures (score 2) across all specimens multiplied by 0.25, the percentage of half fused sutures (score 3) across all specimens multiplied by 0.5, the percentage of three-quarter fused sutures (score 4) across all specimens multiplied by 0.75, and the percentage of entirely fused sutures (score 5) across all specimens (Equation 1), adapted from Goswami *et al*. (2013).

(1) $$\begin{array}{l} \;Suture\;closure\;score\;=(\text{\%})=(\text{\%}(\;score\;2)\times 0.25)\\ \;+(\text{\%}(\;score\;3)\times 0.5)\\ \;+(\text{\%}(\;score\;4)\times 0.75)\\ \;+(\text{\%}\;(\;score\;5)) \end{array}$$

Total suture closure score (%) was similarly calculated for each specimen separately, combining the fusion data for all sutures pertaining to the same specimen using Equation 1. For each species, an average suture closure score was calculated for each developmental stage (F, I, SA, A). For both suture closure scores and total suture closure scores, 100% indicates an entirely fused suture, whereas 0% indicates an open suture.

Using the adult species’ total suture closure scores, ancestral total suture closure scores were estimated, by implementing the ‘*anc.ML*’ function in the *phytools* R package (Revell 2012), as described by Morris et al. (2019). Ancestral states were estimated under an assumption of Brownian evolution using the trimmed time-calibrated maximum clade credibility phylogeny from Upham *et al*. (2019). Estimated ancestral total suture closure scores were mapped as continuous characters on to the phylogeny using the ‘*contMap*’ function in the *phytools* R package (Revell 2012).

### Correlates of suture closure

First, the influence of developmental origin (neural crest, mesoderm, or boundary) on the degree of suture fusion was assessed by performing an ANOVA for suture closure scores and developmental origin across the dataset. A phylogenetic ANOVA was not performed here, as all species were combined to obtain a suture closure score. Second, the correlation between total suture closure score and log-transformed CS was used to estimate the relationship between fusion and skull size. This relationship was quantified using Spearman’s rank correlation coefficient both across all specimens and within each species, by implementing the ‘*spearman*’ option in the ‘*cor.test*’ function in the *stats* R package (R Core Team 2017). For the adult-only dataset (*N* = 22), this relationship between fusion and size was calculated by implementing the ‘*gls*’ function in the *nlme* R package (Pinheiro and Bates 2000) under an assumption of Brownian evolution.

### Rank order

To determine the pattern of suture closure, each suture was assigned a closure rank (1–31), depending on the suture closure score, as outlined by Goswami *et al*. (2013). The earliest fusing suture (highest suture closure score) was assigned a closure rank of 1 and the latest fusing suture (lowest suture closure score) was assigned a closure rank of 31. As Kendall’s tau tests for the conservation of a sequence or rank order within a set of traits (Goswami *et al*. 2009, 2013), it was used to perform pairwise comparisons across the 22 species to assess the similarity in the order of suture closure. Kendall’s tau was implemented using the ‘*kendall*’ option in the ‘*cor.test*’ function in the *stats* R package (R Core Team 2017) with a Bonferroni correction to adjust for multiple comparisons (*P* < 0.05).

Each of the sutures was assigned to one of the six Krogman regions (Krogman 1930) (Table 1), which have been proposed to fuse in the following order: vault (1), base (2), circum-meatal (3), palatal (4), facial (5), and cranio-facial (6). To assess whether the closure order of the dataset follows the Krogman pattern, suture closure scores were averaged across the Krogman regions and ranked between 1 and 6 (first and last to fuse). Kendall’s tau (*P* < 0.05) was implemented to assess similarity in the order of fusion for the adult dataset (*N* = 22) to Krogman’s order. The datasets generated and analysed throughout our study are available at the Github repository: https://github.com/HeatherEWhite/mammal_suture_fusion (White *et al*. 2023b).

## Results

### Suture closure for suture locales (adult suture closure score)

To quantify variation in suture patency across the analysed sutures (*N* = 31), closure scores pertaining to a single suture were combined for all adult specimens (Equation 1) to calculate a suture closure score at the fully developed state across mammals. The highest suture closure scores in the adult-only dataset were the alispheno-squamosal (90.90%), interfrontal (89.77%) and fronto-parietal (89.77%) sutures (Supporting Information, Table S9). These highly fused sutures are predominantly within the cranial vault (Table 1). In contrast, sutures with the lowest suture closure score, thus the most patent sutures across the dataset, were the jugo-squamosal (53.41%), basispheno-presphenoid (59.09%), and basispheno-basioccipital (60.23%) sutures. The jugo-squamosal presented with the lowest fusion due to the absence of the jugal bone in several species, while the remaining sutures with low closure scores are associated with the cranial base. No significant association between suture closure score and the developmental origin of the suture (mesoderm, neural crest, or boundary tissue) was identified for either the full dataset or adults-only (ANOVA: *P* = 0.292 or *P* = 0.186, respectively) (Supporting Information, Fig. S4).

### Suture closure for species (adult total suture closure score)

**Figure 2:**
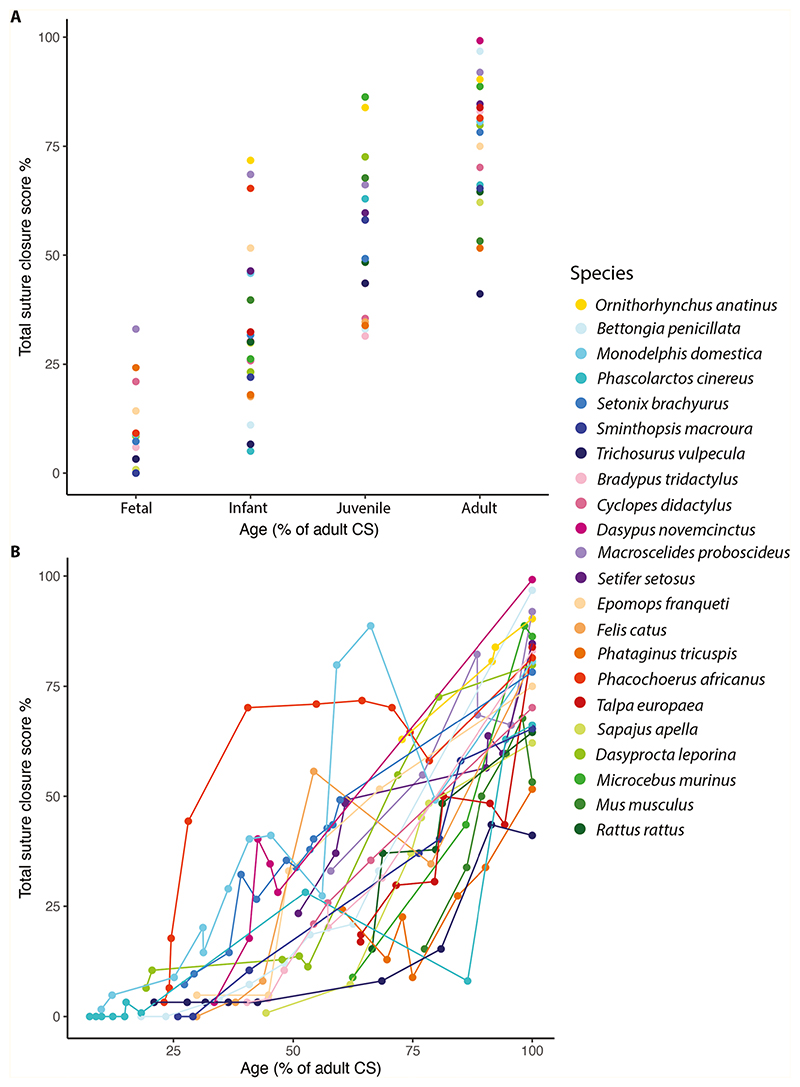
Ancestral state estimation of total suture closure scores based on ectocranial analysis. Branch colours correspond to the total suture closure score, where blue indicates a higher total suture closure score (%) and red indicates a lower total suture closure score (%). Full results can be found in the Supporting Information, Table S11.

To quantify the degree of overall suture patency for each mammalian species analysed here, closure scores pertaining to a single adult specimen were combined (Equation 1) to calculate a total suture closure score at the fully developed stage. Analysis of the adult-only dataset showed that total suture closure score (Supporting Information, Table S10) ranged from 41.13% to 99.19%. *Dasypus novemcinctus* (99.19%), *Bettongia penicillata* (96.77%), and *Macroscelides proboscideus* (91.94%) presented with the highest total suture closure scores. *Trichosurus vulpecula* (41.13%), *Phataginus tricuspis* (51.61%), and *Mus musculus* (53.23%) displayed the lowest total suture closure scores. A large range in total suture closure score was visible across the marsupials, ranging from 96.77% (*Bettongia penicillata*) to 41.13% (*Trichosurus vulpecula*) (Fig. 2). The only monotreme (*Ornithorhynchus anatinus*) in the dataset had a highly fused adult skull (90.32%). Total suture closure scores were generally high for the studied Xenarthra and Afrotheria species, all of which presented with mid to high levels of fusion (70.16%–99.19%). In contrast, Laurasiatheria and Euarchontoglires fall in the low to mid-range of total suture closure (51.61%–88.71%). Marsupials had, on average, a lower total suture closure score (71.36%) than placentals (76.63%), but this was found to be non-significant. Ancestral state estimations, using adult specimens only, similarly supported this difference between marsupials and placentals. A highly similar level of suture fusion was observed for the ancestral placental node (78.69%) and the ancestral therian node (78.25%) (Supporting Information, Table S11). In contrast, the ancestral marsupial differed from these highly similar placental and therian nodes, with a lower degree of overall suture fusion (72.26%).

Evaluation of suture fusion in the cross-section for adults only (for further details see the supplemental methods and supplemental results sections, as well as Tables S15 and S16 and Figures S2 and S3, within the Supporting Information) showed that species displaying at least some degree of fusion (fused in one or more of the cross-sections) across all three analysed sutures (interfrontal, sagittal, and coronal) were: *Phacochoerus africanus, Ornithorhynchus anatinus, Talpa europaea, Bradypus tridactylus*, and *Microcebus murinus*. Species exhibiting some degree of fusion in all three cross-sectional sutures were also found to be species with high levels of ectocranial fusion. Specifically, these highly fused species from the cross-sectional analysis fell into the 40% of species with the highest degree of ectocranial fusion. Nevertheless, there were some differences between ectocranial and cross-sectional fusion; for example, the three species with the highest overall ectocranial fusion (*Dasypus novemcinctus, Bettongia penicillata*, and *Macroscelides proboscideus*) did not exhibit fusion for all three of the analysed cross-sectional sutures. Whilst similarities are noted between ectocranial and cross-sectional analysis, variation is also noted. The raw closure scores for the three sutures analysed in cross-section, and the same three sutures analysed ectocranially, highlight differences in the level of fusion. Several species with highly fused ectocranial sutures appear open when scored in the cross-section (*Bettongia penicillata, Macroscelides proboscideus, Phascolarctos cinereus*, and *Setifer setosus*) (Supporting Information, Table S12).

### Suture closure across ontogeny (developmental total suture closure score)

**Figure 3:**
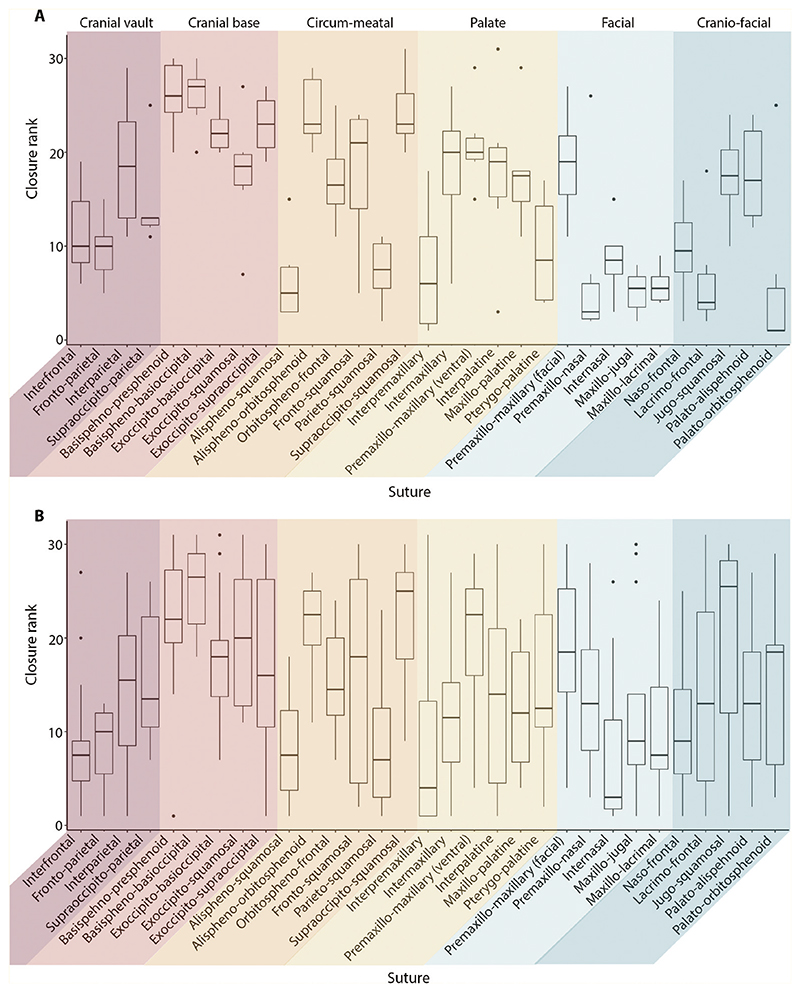
Change in total suture closure scores across ontogeny, based on ectocranial analysis. A, ontogenetic stage is binned into four discrete age categories: foetal, infant, late infant, adult; B, ontogeny is captured as a continuous variable, calculated as the percentage of adult centroid size, where the adult reflects 100%. Colours indicate the species analysed (N = 22).

Beyond the analysis of adult-only specimens, total suture closure scores were studied across ontogeny, where, as expected, an increase in fusion was observed with developmental age (Fig. 3). Several species display fluctuations in the level of suture fusion across ontogeny, probably the result of intraspecific variation within the dataset. Euarchontoglirans show a delayed onset of fusion, followed by rapid fusion occurring in the later developmental stages, relative to other placentals. Similarly, marsupial species presented with a very low level of suture fusion in the earliest sampled stages (Fig. 3B), whereas rapid suture fusion was observed in the early developmental stages of the laurasiatherian placental *Phacochoerus africanus*.

### Suture closure and skull size

Total suture closure score was significantly and positively correlated (Spearman’s ρ = 0.5; *P* < 0.001) with skull size (logCS) across the ontogenetic comparative dataset (*N* = 165) (Supporting Information, Fig. S5). For the 22 species, a significant positive correlation between total suture closure score and skull size (*P* < 0.05) was identified for 18 of the 22 sampled species, with the exceptions of *Dasypus novemcinctus, Macroscelides proboscideus, Phataginus tricuspis*, and *Microcebus murinus* (Table 2; Supporting Information, Table S13). However, when only the adult specimens were considered, no correlation (Spearman’s ρ = 0.087; *P* = 0.700) was found between the degree of suture closure and skull size, which remained non-significant after accounting for phylogenetic relatedness (AIC = 194.145; *P* = 0.392) (Supporting Information, Fig. S6).

### Pattern of suture fusion

Across species, extensive variation was observed in the pattern of suture fusion, calculated using suture closure scores (Supporting Information, Fig. S7, Table S14). Nevertheless, the facial region, for the most part, fused early and the cranial base fused last. The circum-meatal was the most variable region. Only 10 of 231 pairwise comparisons across the 22 species comparing the pattern of suture closure, were significantly correlated following a Bonferroni correction (*P* < 0.05).

**Figure 4:**
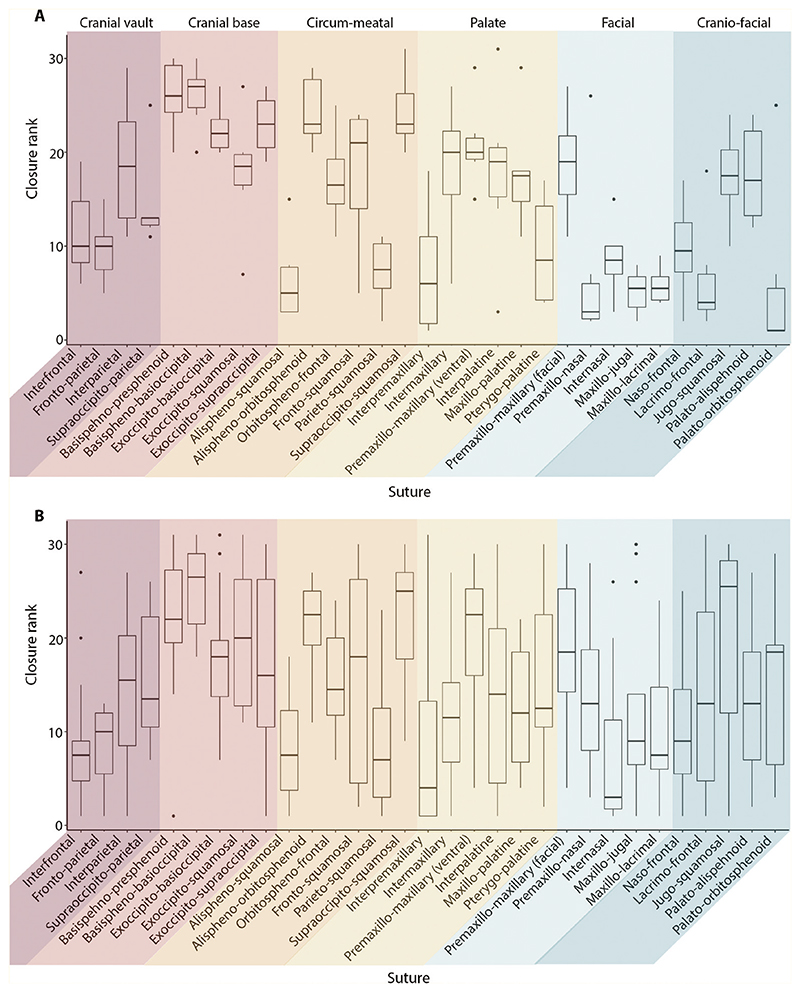
Distribution of closure ranks, based on ectocranial analysis, for each suture (*N* = 31) across every specimen: A, marsupials (*N* = 6); B, placentals (*N* = 15). Sutures grouped by Krogman region (1930).

Separate analyses of marsupials and placentals indicated a much stronger pattern of suture fusion (Fig. 4; Table 3). Marsupials presented with less variability in the suture closure rank than the respective sutures for placentals. Therefore, a clearer pattern of suture fusion was observed for the marsupials where the facial region fused first, alongside several sutures of the cranio-facial region, followed by the cranial vault. The palate and cranial base fused last, whilst the circum-meatal remained highly variable. This pattern of early fusion of the marsupial craniofacial and facial regions was corroborated by the total suture closure scores through ontogeny (Supporting Information, Fig. S8E, F). In contrast, the placentals showed more variability, which reflected the high variation that was observed in the combined dataset (Supporting Information, Fig. S7). Compared to the marsupials, the placentals instead experienced a delayed fusion of the facial sutures. Moreover, the sutures of the cranial base and palate appeared to fuse earlier in placentals than in the marsupials, alongside two sutures of the vault (interfrontal and fronto-parietal). Nevertheless, the fusion of the cranial base was still largely delayed during ontogeny compared to the other cranial regions, with many species indicating no fusion during early development (Supporting Information, Fig. S8B).

Consistent with the Krogman pattern (1930), the cranial vault was the most fused region in the adult dataset (86.65%) (Table 4). Beyond this region, no correlation (*P* > 0.05) between the Krogman pattern and the overall fusion pattern of the dataset was identified. However, when the dataset was subdivided into marsupials and placentals, a significant correlation was identified between the average placental pattern of suture closure and the Krogman pattern of closure (Kendall’s τ = 0.828; *P* = 0.022), although high variation was observed between individual species and the Krogman pattern. Specifically, only three of the 15 placental species displayed a significant positive correlation with the Krogman pattern (Supporting Information, Table S13). This result, however, did not extend to the monotreme or the marsupials, which showed no correlation to the Krogman pattern (respectively: Kendall’s τ = 0; *P* = 1; and Kendall’s τ = –0.2; *P* = 0.573). The monotreme within the dataset (*Ornithorhynchus anatinus*) was, however, only represented by one species and had no foetal or early developmental stages. Therefore, the majority of sutures for *Ornithynchus anatinus* presented with a very high level of fusion (degree of overall fusion: 90.32%), meaning that it was difficult to reliably determine a pattern of suture fusion pattern in monotremes. The average marsupial pattern differed significantly from the Krogman pattern and instead fused in the following order: vault, cranio-facial, facial, circum-meatal, palate, and cranial base.

## Discussion

Sutures support a myriad of functions. Largely through their patency, cranial sutures act to redistribute strain across the skull, accommodate brain expansion, support cranial bone growth, and allow joint movement during feeding (Opperman 2000, Morriss-Kay 2001, Rayfield 2005, Flores *et al*. 2006, Lana-Elola *et al*. 2007, Moazen *et al*. 2009, Goswami *et al*. 2013, Lesciotto and Richtsmeier 2019). Assessing suture fusion with ontogenetic data provides direct information on the degree and pattern of suture fusion and thus the importance of fusion for the developing skull. Using the most detailed approach to date to gather information on both ectocranial and cross-sectional suture fusion, we have captured the degree of ectocranial suture fusion for 31 sutures across 22 species of mammal in a comparative ontogenetic framework (*N* = 165). Our results provide new insights into the many aspects of the marsupial–placental dichotomy. Ancestral state estimations of the overall degree of suture fusion identified similar higher levels of suture fusion for the ancestral therian and ancestral placental than was estimated for the ancestral marsupial. Moreover, suture closure scores indicated the early fusion of cranial vault sutures across all studied taxa, although the subsequent order of fusion varied greatly between marsupials and placentals. The average placental suture fusion pattern correlated with the Krogman pattern, whilst marsupials deviated from this order. Deviation in the marsupial pattern was seen primarily through the early fusion of the facial region and the late fusion of the cranial base.

### Variation between ectocranial and cross-sectional suture fusion

Similarities are observed between ectocranial and cross-sectional analysis. Species undergoing cross-sectional suture fusion were found to have higher levels of ectocranial fusion (*Bradypus tridactylus, Microcebus murinus, Phacochoerus africanus, Ornithorhynchus anatinus*, and *Talpa europaea*). Nevertheless, variation between the two approaches was also noted. One explanation for this difference between ectocranial and cross-sectional fusion is that suture voxels were added to the 3D cranial models during the mesh reconstruction process when grayscale thresholding was performed. However, it is possible that suture fusion varies between the ectocranial and endocranial surfaces, as has been previously suggested in the literature (Krogman 1930, Sun *et al*. 2004). In some species, fusion is reported to first occur ectocranially, whilst others experience initial fusion from the endocranial aspect (Krogman 1930, Sun *et al*. 2004). Here, in the species where ectocranial scores differed largely from cross-sectional scores, ectocranial fusion was visible whilst internally the suture remained patent. It should be noted here, however, that cross-sectional analysis was only scored at three points along the length of the suture rather than the entire suture length as with ectocranial scoring and thus the two approaches are not directly comparable. Sutures such as these, which indicate ectocranial fusion but which remain patent internally, are thus still able to function as sites of interstitial bone growth (Opperman 2000) and provide shock absorption (Moazen *et al*. 2009). Therefore, as indicated here, by cross-sectional analysis on an adult-only subset of the full dataset, ectocranial fusion may not reflect the full picture of suture fusion. Many previous studies (Wilson and Sánchez-Villagra 2009, Sánchez-Villagra 2010, Goswami *et al*. 2013, Rager *et al*. 2014) that have focused on ectocranial analysis, as well as future studies quantifying suture fusion, may, therefore, gain additional insights by including cross-sectional analysis. To note, as cross-sectional fusion was conducted on a small sample of adult-only specimens, subsequent discussion here is based upon ectocranial analysis and thus fusion patterns that are observed ectocranially may not be reflected endocranially.

### Suture fusion as a proxy for developmental age

The significant positive correlation between total suture closure score and centroid size [a known proxy for developmental age (Zelditch *et al*. 2012, Navalón *et al*. 2021)] across all species unsurprisingly corroborates the expectation that, as developmental age increases, so too does the level of suture fusion. This supports previous studies that identified a similar correlation between suture fusion and skull length (Wilson and Sánchez-Villagra 2009, Goswami *et al*. 2013), and suture fusion and body size (Bärmann and Sánchez-Villagra 2012).

The relationship between fusion and skull size has previously been implemented to determine ontogenetic age of fossil specimens of Dinosauria and Synapsida (Sampson *et al*. 1997, Sánchez-Villagra 2010, Fujiwara and Takakuwa 2011, Longrich and Field 2012), and is commonly used to assess hominid age in archaeological studies (Key *et al*. 1994, Vodanović *et al*. 2011, Wood 2015, Ubelaker and Khosrowshahi 2019). Given the significant correlation between suture fusion and centroid size across the dataset, suture fusion might, therefore, provide a reliable predictor for developmental age in mammalian species. Concerns, however, have been expressed several times within the field of archaeology, regarding the reliability of this age-determination approach (Powers 1962, Sahni *et al*. 2005). Such concerns about the use of suture fusion for age approximation also hold here, as not all species had a significant correlation with centroid size and many species displayed some degree of intraspecific variation. Specifically, degree of fusion in *Dasypus novemcinctus* (xenarthran), *Macroscelides proboscideus* (afrotherian), *Phataginus tricuspis* (laurasiatherian), and *Microcebus murinus* (euarchontogliran) showed no correlation with centroid size, thus suggesting that suture fusion is not a reliable discriminator of age for species in each of the placental superorders. In contrast, all marsupial specimens and the monotreme had a significant correlation between degree of fusion and centroid size, indicating that suture fusion may act as a suitable age discriminator for these infraclasses. Nevertheless, increased sampling is required to assess whether this pattern holds. Our analyses combine closure scores for all cranial sutures pertaining to a single specimen to obtain an overall level of cranial suture fusion (Equation 1), as has been previously implemented (Wilson and Sánchez-Villagra 2009, Bärmann and Sánchez-Villagra 2012, Goswami *et al*. 2013). However, specific sutures have been suggested to be more reliable predictors of age than others (maxilla–premaxilla and spheno-occiptal sutures) (Flores and Barone 2012); therefore, this approach of combining all cranial sutures might mislead age estimates and overestimate their utility for ageing specimens.

### Ecology and suture fusion

A myriad of functions underpins cranial suture form and fusion, e.g. interstitial bone growth, support brain expansion, provide shock absorption during feeding, and strain redistribution (Herrel *et al*. 2000, Opperman 2000, Moazen *et al*. 2009, Cray *et al*. 2010). This vast array of functions performed by a suture thus complicates the biological interpretation of form. Much discussion within the literature has been held about the reliability of interpreting form from function, in particular relating to cranial form (Seilacher 1970, Lauder 1995, Gould 2002). Whilst suture function has previously been linked to ecological factors, our study does not test the influence of any ecological factors on suture fusion. We do, however, highlight some potentially interesting links to ecology based on previous work and propose avenues for future research.

Rapid fusion observed in *Phacochoerus africanus* is probably due to its highly developed state at birth, resulting in a reduced demand for sutures to support extensive postnatal cranial bone expansion (DuBrul and Laskin 1961, Opperman 2000). Relative to the other species studied here, *Phacochoerus africanus* displayed a particularly early fusion of the cranial base region (Supporting Information, Fig. S8B). Early fusion of the basisphenoid–basioccipital suture has been associated with increased flexion in the cranial base (DuBrul and Laskin 1961), as is observed in *Phacochoerus africanus* (Supporting Information, Fig. S9). This phenomenon of cranial base flexion has predominantly been associated with encephalization in humans (Virchow 1857, Ranke 1892, Ross and Ravosa 1993, Ross *et al*. 2004, Bastir *et al*. 2010, Lesciotto and Richtsmeier 2019) and has also been linked to the vertical posture of the neck (DeBrul and Laskin 1961, Ross 1995). However, as the neck of *Phacochoerus africanus* is virtually horizontal (to the Frankfurt plane) and its cranial vault is comparatively small to the rest of its skull, neither explanation elucidates the reason for such pronounced cranial base flexion occurring from this early suture fusion. Similarly, in other species of Artiodactyla, no correlation between cranial base flexion and neck posture was found, even in the famously vertical necked species (*Giraffa* and *Okapia*) (Bärmann and Sánchez-Villagra 2012). Instead, it is possible that the modular nature of the mammalian skull (Goswami 2006, Porto *et al*. 2009, Goswami and Finarelli 2016, Parr *et al*. 2016), which enables various cranial regions to respond semi-independently to selective pressures, might have instead supported the morphological expansion of the facial region compared to other cranial regions and thus led to cranial base flexion. Alternatively, snout foraging behaviour (Edossa *et al*. 2021), observed early in postnatal development for the precocial *Phacochoerus africanus*, might exert a selective pressure that results in rapid fusion of the basisphenoid–basioccipital suture, to support cranial base flexion and thus an enlarged facial region for foraging. A multitude of possibilities are discussed here for differences observed in *Phacochoerus africanus* suture fusion, although none are directly assessed. Nevertheless, the number of possibilities discussed here highlights the myriad of functions underpinning suture fusion and thus the difficulties of teasing out a suture form–function relationship.

A significant correlation between size and fusion was not identified in the adult-only dataset, supporting the findings of Goswami *et al*. (2013) and Rager *et al*. (2014) but contradicting the findings of Wilson and Sánchez-Villagra (2009) and Bärmann and Sánchez-Villagra (2012). As size was not found to be a significant correlate of suture fusion across adult mammalian species, it is highly possible that ecological factors, such as diet or locomotion, provide better explanations for the level of suture fusion. Ruminant species, for example, have a higher proportion of open sutures than non-ruminant species, to enable the deflection of higher shearing stresses across the skull (Herring 1993, Bärmann and Sánchez-Villagra 2012). This link between suture patency and stress deflection has also been found in fossorial species that similarly experience external stress across the skull from their mode of locomotion (Wilson and Sánchez-Villagra 2009). Given the ontogenetic focus of this dataset, the sample size and breadth of adult species used here was too small to test similar ecological hypotheses. Nevertheless, suture fusion and its ecological correlates would provide an interesting avenue for future work implementing a broader comparative dataset.

As suture fusion is not the only component of suture morphology, and that suture morphology is also comprised of shape and complexity, it would be pertinent for future studies aiming to determine a form–function relationship to also quantify the relationship between shape and/or complexity and suture fusion. Such analysis would be important in determining what role function plays in suture fusion, given that suture shape and complexity are known to be associated with varying patterns of stress and strain (Herring 2008) as with suture fusion.

### Placentals, the Krogman pattern, and divergent marsupial fusion

The average suture fusion pattern across placental mammals within the dataset correlated with the pattern proposed by Krogman (1930). This longstanding hypothesis was originally identified in hominoid species (Krogman 1930) and has since been found to hold for several species of Carnivora (Goswami *et al*. 2013) and hystricognath rodents (Wilson and Sánchez-Villagra 2009). Nevertheless, interspecific variation has been reported to result in divergence from the Krogman pattern for many of these species (Wilson and Sánchez-Villagra 2009, Goswami *et al*. 2013), suggesting that the order of suture fusion is probably more complex than a single pattern for all mammalian species. The small number of pairwise correlations observed here between species and the Krogman pattern, despite the significant correlation with the average placental pattern, supports this hypothesis that suture fusion pattern is probably more complex than a single conserved Krogman pattern of suture fusion across Placentalia.

Rank order analysis revealed that cranial vault sutures were the first to fuse both in placentals and marsupials, as with the Krogman pattern. Conserved early fusion of the cranial vault for both marsupials and placentals could be due to region-specific signalling pathways. As the cranial vault overlays the brain, growth and transcription factors from the dura mater are known to be central for maintaining cranial vault suture patency (Opperman *et al*. 1993, 1995, Roth *et al*. 1996). Cessation of such signalling could result in the early fusion of the cranial vault sutures relative to the other Krogman regions. This early fusion may, therefore, act to strengthen the cranial vault region and protect the brain.

Beyond the early fusion of the cranial vault, marsupials diverged in their pattern of suture fusion from placentals and the Krogman pattern. Therefore, we here propose a new pattern of suture fusion specific to marsupials: vault, cranio-facial, facial, circum-meatal, palate, cranial base. Nevertheless, variation is observed across marsupial taxa within this dataset. As this study presents a large comparative ontogenetic dataset across a broad range of mammalian taxa, increasing species’ sampling in this ontogenetic framework presents a challenge, due to limited ontogenetic specimen availability within museum collections. However, suture fusion pattern could be determined using the methods of Smith (2001a, 2008) for an adult-only dataset, which would vastly improve taxonomic sampling. Increased sampling across marsupials would, therefore, be necessary for confirming or rejecting this hypothesis of marsupial suture fusion.

Subsequent variation in the pattern of fusion following the early cranial vault fusion of both marsupials and placentals could arise from different behavioural, ecological, and life history demands resulting in differing regional biomechanical stresses that are known to influence suture fusion (Herring 1972, 2008). Of these factors producing differing biomechanical stresses, life history differences may drive marsupial–placental divergences. Specifically, early fusion of the cranio-facial and facial regions is probably driven by the need to strengthen this region to meet the functional demand of continuously suckling at the teat for a prolonged period (Smith 1997, 2001a, 2006, Goswami *et al*. 2016). Furthermore, suture development is thought to be shaped by strain variation through ontogeny. For example, brain tissue growth and its associated intracranial pressure are thought to exert quasi-static strain, defined as strain from the growth of neighbouring tissues, acting on sutural growth, especially during the foetal stage (Herring 2008). Variation in the timing and rate of brain development is widely reported in the literature across the marsupial–placental dichotomy (Lillegraven 1975, Tyndale-Biscoe and Renfree 1987, Nunn and Smith 1998, Weisbecker and Goswami 2010, Bennett and Goswami 2013). Such differences in ontogeny and brain development for the marsupial–placental dichotomy may result in variation of quasi-static strains and produce highly divergent patterns of suture fusion, as observed here. Thus, our results suggest that suture patency is directly influenced by brain growth.

### Suture patency in the cranial base enables prolonged craniofacial growth and extended postnatal brain development in marsupials

As aforementioned, the cranial base in marsupials, unlike placentals, is the final region to undergo suture fusion and consequently retaining patency across ontogeny. It is possible that this delayed fusion in the marsupial cranial base could be linked to the divergent timing of marsupial and placental brain development (Lillegraven 1975). Marsupials experience a considerable delay in the development of the brain compared to the oral apparatus (Smith 1997, Nunn and Smith 1998, Weisbecker and Goswami 2010, Goswami *et al*. 2016, Carlisle *et al*. 2017). As a result, marsupial brain development occurs slowly and almost entirely postnatally (Smith 1997, Weisbecker and Goswami 2010). In comparison, their placental counterparts utilize a longer gestation period and the associated maternal basal metabolic rate to support large brain development over a short timeframe (Martin 1981, Isler and van Schaik 2009, Weisbecker and Goswami 2010). Results here suggest that the prolonged patency and delayed fusion of the cranial base bones acts to support extended postnatal growth of the marsupial brain. Interestingly, marsupials and placentals with similar body size have been reported to have comparable brain sizes, which have previously been hypothesized to be the result of extended marsupial lactation (Weisbecker and Goswami 2010). Our findings suggest that the development of these comparable brain sizes may additionally be supported by this delayed marsupial cranial base fusion, given the early fusion of the cranial vault for both marsupials and placentals. Further evidence for this delayed cranial base fusion and brain size relationship is provided by the fusion patterns of both primate species within the dataset (*Sapajus apella* and *Microcebus murinus*). Both primate species exhibit late fusion of the cranial base (Supporting Information, Table S12), which similarly might be delayed to support the enlarged primate brain size. Further research that quantifies mammalian brain size alongside suture fusion pattern could be used to test the hypothesis proposed here.

Marsupials and placentals further differed with regards to their overall level of suture fusion. Similarly, to Rager *et al*. (2014), we observed a lower degree of overall suture closure in marsupials compared to placentals, albeit not significantly. Although this difference in suture fusion was not significant, the small number of adult specimens and thus species (*N* = 22) used to calculate overall suture fusion might have contributed to this lack of significance. Alternatively, the low overall level of suture fusion observed in *Trichosurus vulpecula* may have contributed largely to the reduced overall marsupial suture fusion. Thus, further analysis implementing increased sampling across mammalian species is required. Previous research has suggested that marsupial species with the lowest level of suture closure are those with a high bite force (Rager *et al*. 2014), in order to dissipate strains across the skull (Moazen *et al*. 2009). However, marsupial taxa with a high bite force identified in Rager *et al*. (2014) were not sampled in the current dataset and thus additional factors must be associated with the low suture closure level observed here. As patent sutures are known to act as sites of interstitial bone growth (Opperman 2000) through the action of suture mesenchymal cells (Lana-Elola *et al*. 2007), the overall reduced patency of cranial sutures and, in particular, the delayed fusion of the cranial base sutures, is instead hypothesized here to play an active role in supporting the extended period of postnatal marsupial cranial growth (Smith 2006). This extended cranial growth, in turn, may accommodate for the slow postnatal brain expansion of marsupials (Smith 1997, Weisbecker and Goswami 2010), whilst the early fusion of the cranial vault may provide protection for the underlying brain development. A similar pattern of delayed cranial base contact and early cranial vault contact has previously been reported in marsupials (Spiekman and Werneburg 2017). Nevertheless, variation in cranial ossification and development observed between marsupials and placentals (Nunn and Smith 1998, Bennett and Goswami 2013) may predispose marsupial taxa to a lower overall level of suture fusion.

The reduced level of overall suture fusion observed in marsupials was also reflected in the estimated fusion of the ancestral marsupial compared to that of the ancestral placental. Interestingly, the lower degree of suture fusion observed in the ancestral marsupial also differed highly from the monotreme, as well as the ancestral therian, which closely reflected the ancestral placental. It is important to note here, that limitations of ancestral state estimation relevant to this dataset are highlighted in White *et al*. (2023a) and include longer phylogenetic branches between the ancestral therian and ancestral marsupial compared to the ancestral placental, differences in the number of placental and marsupial taxa, and a lack of early stages for *Ornithorhynchus anatinus*. Similarly, to White *et al*. (2023a), when accounting for different numbers of marsupial and placental species and a lack of foetal specimens for certain species, differences between the ancestral marsupial and the ancestral therian were still identified. This finding supports the recently published studies of Weaver *et al*. (2022), White *et al*. (2023a), and the minor view of marsupial evolution (Renfree 1993, Smith 2001b), thus rejecting the majority view that the marsupial state is more reminiscent of the ancestral therian state and that placentals represent the more derived condition (Lillegraven 1975, Russell 1982, Lillegraven *et al*. 1987, Maier 1993, Cooper and Steppan 2010). Instead, marsupial suture fusion, or lack thereof, reflects the more derived mammalian state that is probably related to their unique developmental strategy of a short gestation time and extended period of pre-weaning postnatal growth (Lillegraven 1975, Russell 1982, Lillegraven *et al*. 1987, Weisbecker and Goswami 2010). This heightened suture patency, which is associated with the unique marsupial developmental strategy, acts to support prolonged interstitial bone growth (Opperman 2000), which itself facilitates the extended period of marsupial postnatal craniofacial development, in a self-reinforcing loop.

## Conclusion

Suture fusion and patency are reported to have a myriad of functions in supporting cranial growth and overall cranial functioning. Nevertheless, sutures have received considerably less attention in the literature than overall shape of the cranium has, despite underlying its function and development. As expected, suture fusion increases with developmental age, similar to many previous palaeontological and archaeological studies, although we suggest that the use of suture fusion as a proxy for developmental age should be implemented with caution given the complex nature of sutures and species-specific variation. Importantly, we found marsupials to have a highly divergent pattern of suture fusion compared to placentals and the Krogman pattern (1930). Instead, we propose a new pattern of suture fusion for marsupials: vault, cranio-facial, facial, circum-meatal, palate, cranial base. This new pattern of marsupial suture fusion has added to our understanding of the importance sutures have in driving craniofacial variation. Early fusion of the marsupial cranio-facial and facial regions in comparison to placentals is probably driven by the functional demands placed on the marsupial oral apparatus. In contrast, prolonged expansion of the marsupial brain appears to be supported by the extended patency of the cranial base sutures, which provide active sites of interstitial cranial bone growth and expansion. Moreover, variation in the overall level of suture fusion between marsupials and placentals amplifies the differences observed at this boundary and adds to our understanding of the marsupial–placental dichotomy. Collectively, our results suggest that the highly variable reproductive strategies of placentals and marsupials may drive divergences beyond ossification timings to encompass both suture fusion timings and patterns. Consequently, these variations in suture fusion may have knock-on implications for cranial morphology; untangling this relationship requires further study jointly analysing cranial and sutural morphological data.

## Supplementary Material

Supplementary data are available at *Zoological Journal of the Linnean Society* online.

Supplementary Material

Supplementary Table S3

## Figures and Tables

**Table 1:** Sutures and synchondroses (collectively referred to as sutures) and their corresponding Krogman region (1930) analysed ectocranially in this study, established using Goswami *et al*. (2013) and coined based on the articulating bones (Rager *et al*. 2014).

Suture	Krogman region
Interfrontal	Vault (1)
Fronto-parietal	Vault (1)
Interparietal	Vault (1)
Supraoccipito-parietal	Vault (1)
Basispheno-presphenoid	Cranial base (2)
Basispheno-basioccipital	Cranial base (2)
Exoccipito-basioccipital	Cranial base (2)
Exoccipito-squamosal	Cranial base (2)
Exoccipito-supraoccipital	Cranial base (2)
Alispheno-squamosal	Circum-meatal (3)
Alispheno-orbitosphenoid	Circum-meatal (3)
Orbitospheno-frontal	Circum-meatal (3)
Fronto-squamosal	Circum-meatal (3)
Parieto-squamosal	Circum-meatal (3)
Supraoccipito-squamosal	Circum-meatal (3)
Interpremaxillary	Palate (4)
Intermaxillary	Palate (4)
Premaxillo-maxillary (ventral)	Palate (4)
Interpalatine	Palate (4)
Maxillo-palatine	Palate (4)
Pterygo-palatine	Palate (4)
Premaxillo-maxillary (facial)	Facial (5)
Premaxillo-nasal	Facial (5)
Internasal	Facial (5)
Maxillo-jugal	Facial (5)
Maxillo-lacrimal	Facial (5)
Naso-frontal	Cranio-facial (6)
Lacrimo-frontal	Cranio-facial (6)
Jugo-squamosal	Cranio-facial (6)
Palato-alisphenoid	Cranio-facial (6)
Palato-orbitosphenoid	Cranio-facial (6)

**Table 2:** Spearman’s rank correlations between total suture closure score, based on ectocranial analysis, and skull size (logged centroid size) for each species (*N* = 22), where bold indicates a significant correlation (*P* < 0.05).

Species	Spearman’s ρ	*P*-value
*Bettongia penicillata*	0.999	**< 0.001**
*Bradypus tridactylus*	0.964	**< 0.001**
*Cyclopes didactylus*	1.000	**< 0.001**
*Dasyprocta leporina*	0.929	**< 0.001**
*Dasypus novemcinctus*	0.771	0.072
*Epomops franqueti*	0.975	**0.004**
*Felis catus*	0.955	**< 0.001**
*Macroscelides proboscideus*	0.771	0.072
*Microcebus murinus*	0.800	0.200
*Monodelphis domestica*	0.934	**< 0.001**
*Mus musculus*	0.829	**0.042**
*Ornithorhynchus anatinus*	1.000	**< 0.001**
*Phacochoerus africanus*	0.745	**0.008**
*Phascolarctos cinereus*	0.934	**< 0.001**
*Phataginus tricuspis*	0.679	0.094
*Rattus rattus*	1.000	**< 0.001**
*Sapajus apella*	1.000	**< 0.001**
*Setifer setosus*	0.976	**< 0.001**
*Setonix brachyurus*	0.989	**< 0.001**
*Sminthopsis macroura*	0.991	**< 0.001**
*Talpa europaea*	0.905	**0.002**
*Trichosurus vulpecula*	0.895	**0.001**

**Table 3:** Order of suture fusion for the marsupials (6 species) and placentals (15 species), based on ectocranial analysis. Sutures are ordered chronologically, starting with the first suture to fuse.

Marsupials	Placentals
Parieto-squamosal	Interfrontal
Pterygo-palatine	Exoccipito-supraoccipital
Fronto-parietal	Exoccipito-basioccipital
Maxillo-jugal	Alispheno-squamosal
Naso-frontal	Internasal
Palato-orbitosphenoid	Fronto-parietal
Alispheno-squamosal	Interparietal
Supraoccipito-parietal	Parieto-squamosal
Exoccipito-squamosal	Naso-frontal
Maxillo-lacrimal	Supraoccipito-parietal
Lacrimo-frontal	Supraoccipito-squamosal
Interfrontal	Maxillo-palatine
Fronto-squamosal	Maxillo-lacrimal
Interpremaxillary	Orbitospheno-frontal
Palato-alisphenoid	Pterygo-palatine
Interparietal	Palato-alisphenoid
Orbitospheno-frontal	Palato-orbitosphenoid
Premaxillo-nasal	Interpremaxillary
Exoccipito-supraoccipital	Interpalatine
Exoccipito-basioccipital	Lacrimo-frontal
Interpalatine	Alispheno-orbitosphenoid
Maxillo-palatine	Intermaxillary
Premaxillo-maxillary (facial)	Maxillo-jugal
Supraoccipito-squamosal	Fronto-squamosal
Intermaxillary	Basispheno-basioccipital
Premaxillo-maxillary (ventral)	Exoccipito-squamosal
Jugo-squamosal	Basispheno-presphenoid
Internasal	Premaxillo-maxillary (facial)
Alispheno-orbitosphenoid	Premaxillo-nasal
Basispheno-presphenoid	Premaxillo-maxillary (ventral)
Basispheno-basioccipital	Jugo-squamosal

**Table 4:** Order of Krogman region fusion for the placentals and marsupials compared to the Krogman pattern (1930), ordered chronologically, starting with the region to fuse first, based on ectocranial analysis. Order calculated from the average percentage of fusion across all sutures of the region for adult only specimens.

Krogman pattern	Full dataset pattern	Placental pattern	Marsupial pattern
Vault	Vault (86.36%)	Vault (87.92%)	Vault (83.04%)
Cranial base	Circum-meatal (77.08%)	Circum-meatal (79.17%)	Cranio-facial (82.14%)
Circum-meatal	Cranio-facial (75.68%)	Cranial base (76.33%)	Facial (75.00%)
Palate	Facial (73.41%)	Palate (73.33%)	Circum-meatal (72.62%)
Facial	Palate (72.54%)	Facial (72.67%)	Palate (70.83%)
Cranio-facial	Cranial base (72.27%)	Cranio-facial (72.67%)	Cranial base (63.57%)

## Data Availability

3D scan data [as published in White *et al*. (2023a)] is available at: https://www.morphosource.org/ (to be made publicly available when accepted). All original code used within this study is, as of the date of publication, publicly available at: https://github.com/HeatherEWhite/mammal_suture_fusion (White *et al*. 2023b) (Zenodo DOI to be published when manuscript is accepted). All code and raw data are available to download, complete with MIT licence. Please cite this paper and the Zenodo DOI: (Zenodo DOI to be published when manuscript is accepted) when using the data or raw code.
