# Supplementary material for: Divergent patterns of cranial suture fusion in marsupial and placental mammals
Source: Zool J Linn Soc. Author manuscript; Available in PMC 2025 Feb 24. (PMC7617424; doi:10.1093/zoolinnean/zlae060)
Supplement: Supplementary Material [file EMS203323-supplement-Supplementary_Material.pdf]

SUPPLEMENTAL MATERIAL

SUPPLEMENTAL METHODS

Cross-sectional suture fusion

Previous work considering suture fusion, in a comparative evolutionary framework, has tended to focus on the ectocranial fusion of the sutures, i.e., the fusion at the external or surficial cranial surface (Wilson and Sánchez-Villagra, 2009; Bärmann and Sánchez-Villagra, 2012; Goswami *et al.* 2013; Rager *et al.* 2014). However, as sutures are inherently three-dimensional structures, the study of the ectocranial component only disregards their internal component and thus its endocranial fusion (internal). Work that explores the cross-sectional aspect of the suture tends to focus on model organisms, specifically the histology and thus the cellular makeup of the suture rather than the cross-sectional morphology or fusion (Opperman, 2000; Lana-Elola *et al.* 2007; Topczewska *et al.* 2016). Interestingly, one study has performed an analysis of cross-sectional suture histology from a phylogenetic perspective focussing on two species of non-avian archosaurs (*Dromaius novaehollandiae* and *Alligator mississippiensis*) (Bailleul and Horner, 2016). As the aforementioned studies focus on the cellular makeup rather than fusion or morphology, there is limited understanding, outside of model organisms, as to whether suture closure also applies to the endocranial or internal aspects of a suture, or simply its ectocranial aspect. Furthermore, the extent of cross-sectional suture fusion may also impact suture function, and thus is important to consider when reconstructing the evolution and development of sutures.

Cross-sectional suture fusion was assessed on the X-ray projection slices (Figure S3), whereas ectocranial fusion was scored on the tomographic reconstructions. Cross-sectional sutures were scored as either open (0), partially closed (½), or closed (1) in the adult specimens only (n=21). Cross-sectional analysis was used to determine whether surficial (ectocranial) fusion was observed in tangent with endocranial or cross-sectional suture fusion. *Dasypus novemcinctus* was excluded from this analysis as no internal features were visible for this 3D surface scanned specimen. Due to the time-consuming nature of assessing longitudinal (cross-sectional) suture fusion, only three sutures of the cranial vault were assessed (interfrontal, sagittal, and coronal sutures), due to their functional importance as well as variation in developmental origin (see below). Given that individual sutures are known to fuse at varying rates along their length, for example the posterior portion of the interfrontal suture undergoes fusion compared to the anterior portion which

remains patent (Opperman, 2000), each suture was therefore assessed at three positions along its length. For the interfrontal and sagittal sutures this equated to an anterior and posterior section as well as the section equidistant between the two, whereas for the coronal suture this equated to a near cranial midline section and the most lateral section, as well as the section equidistant between the two (Figure S2). Classification of cross-sectional suture fusion status was performed directly using the original X-ray images of micro-CT scans. The crosshair function in Avizo v.9.3 (FEI, Hillsboro, OR, USA) was used to determine the correct location of the suture in the coronal plane for the interfrontal and sagittal sutures and the sagittal plane for the coronal suture. Each cross-section was scored as either open (0), partially fused (½), or fused (1) (Figure S3). A total of 9 observations were made for each species, equating to 189 observations across all 21 species.

The three sutures analysed for cross-sectional fusion were of particular interest to our study with a focus on developmental patterns due to the differing developmental origins of the three sutures; the interfrontal suture forms between the two neural crest derived frontal bones; the sagittal suture forms between the two mesodermal derived parietal bones; and the coronal suture forms at the boundary of the neural crest derived frontal bone and mesoderm derived parietal bone (Morriss-Kay, 2001). Moreover, these sutures have different functions. As the sagittal suture is positioned within the cranial vault, it is hypothesised to play a critical role in supporting brain expansion and/or braincase integrity at varying developmental stages. In contrast, the interfrontal and coronal sutures are known to experience greater strains and tension during the masticatory process (Herring and Teng, 2000; Rafferty and Herring, 1999). These sutures also capture both the dorsal and coronal anatomical planes, meaning that a better understanding of suture morphology can be drawn from exploring sutures in more than one plane.

SUPPLEMENTAL RESULTS

Cross-sectional suture fusion

Across the 21 species, three sutures, and three positions assessed along each suture (n=189), a total of 138 positions were recorded as open, whereas only 52 were recorded as fused or partially fused. Of the three sutures studied (interfrontal, coronal, and sagittal), five species presented with fully fused (fused in all three cross-sections) interfrontal sutures, compared to four species for both the coronal and sagittal sutures (Table S14 and S15). Of these species with fully fused sutures, only *Phacochoerus africanus* was fully fused across all three sutures. Some species presented with fused sutures along a proportion of the suture length (fused in either one or two of the three cross-sections): three, two, and five species for the interfrontal, coronal, and sagittal sutures respectively. Species that displayed at least some degree of fusion (fused in one or more of the cross-sections) across all three sutures were: *Ornithorhynchus anatinus*, *Talpa europaea*, *Bradypus tridactylus*, and *Microcebus murinus*. These species that displayed at least some degree of fusion across all three sutures, also had relatively higher total suture closure closures, i.e., falling within the upper 40% of species.

Table S1. Species included within the dataset (n=22), including common names, infraclass, placental superorders, Order, and number of specimens (N).

Species	Common Name	Infraclass	Superorder	Order	N
Ornithorhynchus anatinus	Platypus	Monotremata	-	Monotremata	4
Monodelphis domestica	Gray short-tailed opossum	Marsupialia	-	Didelphimorphia	13
Sminthopsis macroura	Stripe-faced dunnart	Marsupialia	-	Dasyuromorphia	7
Phascolarctos cinereus	Koala	Marsupialia	-	Diprotodontia	11
Trichosurus vulpecula	Common brushtail possum	Marsupialia	-	Diprotodontia	9
Setonix brachyurus	Quokka	Marsupialia	-	Diprotodontia	13
Bettongia penicillata	Woylie	Marsupialia	-	Diprotodontia	10
Dasypus novemcinctus	Nine-banded armadillo	Placentalia	Xenarthra	Cingulata	6
Bradypus tridactylus	Pale-throated sloth	Placentalia	Xenarthra	Pilosa	7
Cyclopes didactylus	Silky anteater	Placentalia	Xenarthra	Pilosa	4
Macroscelides proboscideus	Round-eared elephant shrew	Placentalia	Afrotheria	Macroscelidea	6
Setifer setosus	Greater hedgehog tenrec	Placentalia	Afrotheria	Afrosoricida	8
Talpa europaea	European mole	Placentalia	Laurasiatheria	Eulipotyphla	8
Epomops franqueti	Franquet's epauletted fruit bat	Placentalia	Laurasiatheria	Chiroptera	5
Phacochoerus africanus	Common warthog	Placentalia	Laurasiatheria	Artiodactyla	11
Felis catus	Cat	Placentalia	Laurasiatheria	Carnivora	7
Phataginus tricuspis	Tree pangolin	Placentalia	Laurasiatheria	Pholidota	7
Rattus rattus	Black rat	Placentalia	Euarchontoglires	Rodentia	5
Mus musculus	House mouse	Placentalia	Euarchontoglires	Rodentia	6
Dasyprocta leporina	Red-rumped agouti	Placentalia	Euarchontoglires	Rodentia	8
Microcebus murinus	Gray mouse lemur	Placentalia	Euarchontoglires	Primates	4
Sapajus apella	Tufted capuchin	Placentalia	Euarchontoglires	Primates	6

Table S2. Detailed specimen information for the dataset (n=165), including species, specimen number, collaborator information, age, and CT scanner details with scan voxel size. When only one voxel size is provided, this is because the voxel is isometric.

Species	Specimen Number	Myself (Heather White) or Collaborator	Collection Type	CT Scanner	Voxel Size (mm)
Ornithorhynchus anatinus	NHMUK ZD 1859.5.30.4	Heather White	Skull	X-Tek HMX ST 225 (Nikon Metrology, Belgium) (scanned at: NHMUK)	0.0415586
Ornithorhynchus anatinus	NHMUK ZD 1890.12.4.6	Heather White	Skull	X-Tek HMX ST 225 (Nikon Metrology, Belgium) (scanned at: NHMUK)	0.0522641
Ornithorhynchus anatinus	NHMUK ZD 2022.30	Heather White	Spirit	X-Tek HMX ST 225 (Nikon Metrology, Belgium) (scanned at: NHM)	0.0507174
Ornithorhynchus anatinus	NHMUK ZD 1890.12.4.5	Heather White	Skull	XT H 225 ST (Nikon Metrology, Belgium) (scanned at: NHMUK)	0.0403483
Monodelphis domestica	6 days	Karen Sears (Urban *et al.* 2017)	Colony*	Xradia Bio MicroCT (MicroXCT-400) (scanned at: University of Illinois IACUC)	0.0050040
Monodelphis domestica	10 days	Karen Sears (Urban *et al.* 2017)	Colony*	Xradia Bio MicroCT (MicroXCT-400) (scanned at: University of Illinois IACUC)	0.0052349
Monodelphis domestica	UMUT 15 days	Karen Sears (Urban *et al.* 2017)	Colony*	Xradia Bio MicroCT (MicroXCT-400) (scanned at: University of Illinois IACUC)	0.0166390
Monodelphis domestica	20 days	Karen Sears (Urban *et al.* 2017)	Colony*	Xradia Bio MicroCT (MicroXCT-400) (scanned at: University of Illinois IACUC)	0.0089819
Monodelphis domestica	22 days	Karen Sears (Urban *et al.* 2017)	Colony*	Xradia Bio MicroCT (MicroXCT-400) (scanned at: University of Illinois IACUC)	0.0105283
Monodelphis domestica	25 days	Karen Sears (Urban *et al.* 2017)	Colony*	Xradia Bio MicroCT (MicroXCT-400) (scanned at: University of Illinois IACUC)	0.0144504
Monodelphis domestica	30 days	Karen Sears (Urban *et al.* 2017)	Colony*	Xradia Bio MicroCT (MicroXCT-400) (scanned at: University of Illinois IACUC)	0.0165148
Monodelphis domestica	35 days	Karen Sears (Urban *et al.* 2017)	Colony*	Xradia Bio MicroCT (MicroXCT-400) (scanned at: University of Illinois IACUC)	0.0184618
Monodelphis domestica	TMM M-8269	Vera Weisbecker (Ramírez-Chaves *et al.* 2016)	Spirit	Inveon PET-CT (Siemens, USA) (scanned at: University of Queensland)	0.0166 x 0.0166 x 0.03549

Monodelphis domestica	TMM M-7539	Vera Weisbecker (Ramírez-Chaves *et al.* 2016)	Spirit	Inveon PET-CT (Siemens, USA) (scanned at: University of Queensland)	0.0291 x 0.0291 x 0.0676
Monodelphis domestica	TMM M-7542	Vera Weisbecker (Ramírez-Chaves *et al.* 2016)	Spirit	Inveon PET-CT (Siemens, USA) (scanned at: University of Queensland)	0.035 x 0.035 x 0.0608
Monodelphis domestica	TMM M-8268	Vera Weisbecker (Ramírez-Chaves *et al.* 2016)	Spirit	Inveon PET-CT (Siemens, USA) (scanned at: University of Queensland)	0.02148 x 0.02148 x 0.04802
Monodelphis domestica	TMM M-9038	Vera Weisbecker (Ramírez-Chaves *et al.* 2016)	Spirit	Inveon PET-CT (Siemens, USA) (scanned at: University of Queensland)	0.02734 x 0.02734 x 0.06255
Sminthopsis macroura	Smacd19	Vera Weisbecker (Ramírez-Chaves *et al.* 2016)	Colony*	Skyscan 1072 (Bruker, USA) (scanned at: University College London)	0.0039632
Sminthopsis macroura	Smacd22	Vera Weisbecker (Ramírez-Chaves *et al.* 2016)	Colony*	Skyscan 1072 (Bruker, USA) (scanned at: University College London)	0.0039632
Sminthopsis macroura	Smacd31	Vera Weisbecker (Ramírez-Chaves *et al.* 2016)	Colony*	Skyscan 1072 (Bruker, USA) (scanned at: University College London)	0.0141661
Sminthopsis macroura	Smac54dPYA_No44_08	Vera Weisbecker (Ramírez-Chaves *et al.* 2016)	Colony*	Skyscan 1072 (Bruker, USA) (scanned at: University College London)	0.0168660
Sminthopsis macroura	Smacd64d_9_09	Vera Weisbecker (Ramírez-Chaves *et al.* 2016)	Colony*	Skyscan 1072 (Bruker, USA) (scanned at: University College London)	0.0141661
Sminthopsis macroura	SmacPYE74d_No14_08	Vera Weisbecker (Ramírez-Chaves *et al.* 2016)	Colony*	Skyscan 1072 (Bruker, USA) (scanned at: University College London)	0.0168660
Sminthopsis macroura	SmacAdult33_08	Vera Weisbecker (Ramírez-Chaves *et al.* 2016)	Colony*	Skyscan 1072 (Bruker, USA) (scanned at: University College London)	0.0141661
Phascolarctos cinereus	Pcin3-9	Vera Weisbecker (Ramírez-Chaves *et al.* 2016)	Field*	Skyscan 1072 (Bruker, USA) (scanned at: University College London)	0.0141700
Phascolarctos cinereus	Pcin3-7	Vera Weisbecker (Ramírez-Chaves *et al.* 2016)	Field*	Skyscan 1072 (Bruker, USA) (scanned at: University College London)	0.0141700
Phascolarctos cinereus	Pcin3-6	Vera Weisbecker (Ramírez-Chaves *et al.* 2016)	Field*	Skyscan 1072 (Bruker, USA) (scanned at: University College London)	0.0141700

Phascolarctos cinereus	Pcin3-8	Vera Weisbecker (Ramírez-Chaves *et al.* 2016)	Field*	Skyscan 1072 (Bruker, USA) (scanned at: University College London)	0.0168600
Phascolarctos cinereus	Pcin3-5	Vera Weisbecker (Ramírez-Chaves *et al.* 2016)	Field*	Skyscan 1072 (Bruker, USA) (scanned at: University College London)	0.0168600
Phascolarctos cinereus	Pcin3-13	Vera Weisbecker (Ramírez-Chaves *et al.* 2016)	Field*	Skyscan 1072 (Bruker, USA) (scanned at: University College London)	0.0168600
Phascolarctos cinereus	Pcin3-1	Vera Weisbecker (Ramírez-Chaves *et al.* 2016)	Field*	Skyscan 1072 (Bruker, USA) (scanned at: University College London)	0.0250300
Phascolarctos cinereus	KoalasBottomMiddle_spec2	Vera Weisbecker (Ramírez-Chaves *et al.* 2016)	Field*	X-Tek HMX ST 225 (Nikon Metrology, Belgium) (scanned at: Engineering Department of University of Cambridge)	0.1300000
Phascolarctos cinereus	PcinLge05	Vera Weisbecker (Ramírez-Chaves *et al.* 2016)	Field*	X-Tek HMX ST 225 (Nikon Metrology, Belgium) (scanned at: Engineering Department of University of Cambridge)	0.1300000
Phascolarctos cinereus	PcinLge03	Vera Weisbecker (Ramírez-Chaves *et al.* 2016)	Field*	X-Tek HMX ST 225 (Nikon Metrology, Belgium) (scanned at: Engineering Department of University of Cambridge)	0.1300000
Phascolarctos cinereus	PcinLge02	Vera Weisbecker (Ramírez-Chaves *et al.* 2016)	Field*	X-Tek HMX ST 225 (Nikon Metrology, Belgium) (scanned at: Engineering Department of University of Cambridge)	0.1300000
Trichosurus vulpecula	TVAC	Vera Weisbecker (Ramírez-Chaves *et al.* 2016)	Colony*	Skyscan 1072 (Bruker, USA) (scanned at: University College London)	0.0141700
Trichosurus vulpecula	TVPYE	Vera Weisbecker (Ramírez-Chaves *et al.* 2016)	Colony*	Skyscan 1072 (Bruker, USA) (scanned at: University College London)	0.0141700
Trichosurus vulpecula	TVPYK	Vera Weisbecker (Ramírez-Chaves *et al.* 2016)	Colony*	Skyscan 1072 (Bruker, USA) (scanned at: University College London)	0.0141700
Trichosurus vulpecula	TV7	Vera Weisbecker (Ramírez-Chaves *et al.* 2016)	Colony*	Skyscan 1072 (Bruker, USA) (scanned at: University College London)	0.0141700
Trichosurus vulpecula	TV6	Vera Weisbecker (Ramírez-Chaves *et al.* 2016)	Colony*	Skyscan 1072 (Bruker, USA) (scanned at: University College London)	0.0168600
Trichosurus vulpecula	TV8	Vera Weisbecker (Ramírez-Chaves *et al.* 2016)	Colony*	Skyscan 1072 (Bruker, USA) (scanned at: University College London)	0.0155100
Trichosurus vulpecula	TV3	Vera Weisbecker (Ramírez-Chaves *et al.* 2016)	Colony*	Skyscan 1072 (Bruker, USA) (scanned at: University College London)	0.0337300

Trichosurus vulpecula	TV2	Vera Weisbecker (Ramírez-Chaves *et al.* 2016)	Colony*	X-Tek HMX ST 225 (Nikon Metrology, Belgium) (scanned at: Engineering Department of University of Cambridge)	0.0538000
Trichosurus vulpecula	TV1	Vera Weisbecker (Ramírez-Chaves *et al.* 2016)	Colony*	X-Tek HMX ST 225 (Nikon Metrology, Belgium) (scanned at: Engineering Department of University of Cambridge)	0.0475000
Setonix brachyurus	NHMUK ZD 1989.580	Heather White	Spirit	X-Tek HMX ST 225 (Nikon Metrology, Belgium) (scanned at: NHMUK)	0.0279708
Setonix brachyurus	NHMUK ZD 1989.581	Heather White	Spirit	X-Tek HMX ST 225 (Nikon Metrology, Belgium) (scanned at: NHMUK)	0.0279708
Setonix brachyurus	NHMUK ZD 1989.582	Heather White	Spirit	X-Tek HMX ST 225 (Nikon Metrology, Belgium) (scanned at: NHMUK)	0.0294931
Setonix brachyurus	NHMUK ZD 1989.583	Heather White	Spirit	X-Tek HMX ST 225 (Nikon Metrology, Belgium) (scanned at: NHMUK)	0.0309482
Setonix brachyurus	NHMUK ZD 1989.584	Heather White	Spirit	XT H 225 ST (Nikon Metrology, Belgium) (scanned at: NHMUK)	0.0169685
Setonix brachyurus	NHMUK ZD 1989.585	Heather White	Spirit	X-Tek HMX ST 225 (Nikon Metrology, Belgium) (scanned at: NHMUK)	0.0323641
Setonix brachyurus	NHMUK ZD 1989.586	Heather White	Spirit	X-Tek HMX ST 225 (Nikon Metrology, Belgium) (scanned at: NHMUK)	0.0374950
Setonix brachyurus	NHMUK ZD 1989.587	Heather White	Spirit	X-Tek HMX ST 225 (Nikon Metrology, Belgium) (scanned at: NHMUK)	0.0236082
Setonix brachyurus	NHMUK ZD 1989.588	Heather White	Spirit	X-Tek HMX ST 225 (Nikon Metrology, Belgium) (scanned at: NHMUK)	0.0503569
Setonix brachyurus	NHMUK ZD 1989.589	Heather White	Spirit	X-Tek HMX ST 225 (Nikon Metrology, Belgium) (scanned at: NHMUK)	0.0236082
Setonix brachyurus	NHMUK ZD 1989.591	Heather White	Spirit	X-Tek HMX ST 225 (Nikon Metrology, Belgium) (scanned at: NHMUK)	0.0394608
Setonix brachyurus	NHMUK ZD 1989.590	Heather White	Spirit	X-Tek HMX ST 225 (Nikon Metrology, Belgium) (scanned at: NHMUK)	0.0542887
Setonix brachyurus	NHMUK ZD 1906.8.1.245	Heather White	Skull	XT H 225 ST (Nikon Metrology, Belgium) (scanned at: NHMUK)	0.0403484
Bettongia penicillata	SAM 24233	Vera Weisbecker (Ramírez-Chaves *et al.* 2016)	Museum	Helmholtz Zentrum facility (scanner details not available)	0.0096460

Bettongia penicillata	SAM 24239	Vera Weisbecker (Ramírez-Chaves *et al.* 2016)	Museum	Helmholtz Zentrum facility (scanner details not available)	0.0099790
Bettongia penicillata	SAM 24244	Vera Weisbecker (Ramírez-Chaves *et al.* 2016)	Museum	Helmholtz Zentrum facility (scanner details not available)	0.0116420
Bettongia penicillata	SAM 24238	Vera Weisbecker (Ramírez-Chaves *et al.* 2016)	Museum	Helmholtz Zentrum facility (scanner details not available)	0.0149690
Bettongia penicillata	SAM 24230	Vera Weisbecker (Ramírez-Chaves *et al.* 2016)	Museum	Helmholtz Zentrum facility (scanner details not available)	0.0149690
Bettongia penicillata	SAM 24240	Vera Weisbecker (Ramírez-Chaves *et al.* 2016)	Museum	Helmholtz Zentrum facility (scanner details not available)	0.0166320
Bettongia penicillata	SAM 24246	Vera Weisbecker (Ramírez-Chaves *et al.* 2016)	Museum	Helmholtz Zentrum facility (scanner details not available)	0.0191270
Bettongia penicillata	SAM 24228	Vera Weisbecker (Ramírez-Chaves *et al.* 2016)	Museum	Helmholtz Zentrum facility (scanner details not available)	0.0232850
Bettongia penicillata	SAM 24243	Vera Weisbecker (Ramírez-Chaves *et al.* 2016)	Museum	Helmholtz Zentrum facility (scanner details not available)	0.0232850
Bettongia penicillata	SAM 24247	Vera Weisbecker (Ramírez-Chaves *et al.* 2016)	Museum	Helmholtz Zentrum facility (scanner details not available)	0.0357580
Dasypus novemcinctus	ZMB 40641	Lionel Hautier (Hautier *et al.* 2011)	Spirit	Helmholtz Zentrum facility (scanner details not available)	0.0411110
Dasypus novemcinctus	MNHN 1901-331	Lionel Hautier (Hautier *et al.* 2011)	Spirit	Institut des Sciences de l'Evolution (ISEM) (scanner details not available)	0.0358120
Dasypus novemcinctus	ZMB 40651	Lionel Hautier (Hautier *et al.* 2011)	Spirit	Helmholtz Zentrum facility (scanner details not available)	0.0433330
Dasypus novemcinctus	MNHN 1910-410	Lionel Hautier (Hautier *et al.* 2011)	Spirit	Institut des Sciences de l'Evolution (ISEM) (scanner details not available)	0.0358120
Dasypus novemcinctus	MNHN 1910-409b	Lionel Hautier (Hautier *et al.* 2011)	Spirit	Institut des Sciences de l'Evolution (ISEM) (scanner details not available)	0.0358120
Dasypus novemcinctus	z.134	Anjali Goswami	Skull	Surface scanned using the Go!Scan 20 (Creaform)	NA
Bradypus tridactylus	ZMB 9195	Lionel Hautier (Hautier *et al.* 2011)	Spirit	Helmholtz Zentrum facility (scanner details not available)	0.0414290

Bradypus tridactylus	NHMUK ZD 1994.6	Heather White	Spirit	X-Tek HMX ST 225 (Nikon Metrology, Belgium) (scanned at: NHMUK)	0.0340996
Bradypus tridactylus	ZMB 18834	Lionel Hautier (Hautier *et al.* 2011)		Helmholtz Zentrum facility (scanner details not available)	0.0420000
Bradypus tridactylus	NHMUK ZD 1989.226	Heather White	Spirit	X-Tek HMX ST 225 (Nikon Metrology, Belgium) (scanned at: NHMUK)	0.0528368
Bradypus tridactylus	NHMUK ZD 1952.1173	Heather White	Spirit	XT H 225 ST (Nikon Metrology, Belgium) (scanned at: NHMUK)	0.0233930
Bradypus tridactylus	NHMUK ZD 2022.31	Heather White	Spirit	X-Tek HMX ST 225 ((Nikon Metrology, Belgium) (scanned at: NHMUK)	0.0591151
Bradypus tridactylus	NHMUK ZD 1867.4.12.579	Heather White	Skull	X-Tek HMX ST 225 (Nikon Metrology, Belgium) (scanned at: NHMUK)	0.0522641
Cyclopes didactylus	ZMB 6XII1906a	Lionel Hautier (Hautier *et al.* 2011)	Spirit	Helmholtz Zentrum facility (scanner details not available)	0.0328570
Cyclopes didactylus	NHMUK ZD 2010.105	Lionel Hautier (Hautier *et al.* 2011)	Spirit	X-Tek HMX ST 225 (Nikon Metrology, Belgium) (scanned at: NHMUK)	0.0499000
Cyclopes didactylus	NHMUK ZD 1855.12.36.345	Lionel Hautier (Hautier *et al.* 2011)	Spirit	X-Tek HMX ST 225 (Nikon Metrology, Belgium) (scanned at: NHMUK)	0.0619000
Cyclopes didactylus	NHMUK ZD 1926.12.4.68	Heather White	Skull	XT H 225 ST (Nikon Metrology, Belgium) (scanned at: NHMUK)	0.0248667
Macroscelides proboscideus	UMZC 48	Robert Asher (Asher and Olbricht, 2009)	Spirit *	SkyScan 1172 (Scanco Medical AG, Switzerland) (scanned at: University of Cambridge)	0.0239624
Macroscelides proboscideus	NHMUK ZD 1902.9.1.17	Heather White	Skull	XT H 225 ST (Nikon Metrology, Belgium) (scanned at: NHMUK)	0.0120855
Macroscelides proboscideus	UMZC 2021.37	Robert Asher (Asher and Olbricht, 2009)	Skull *	X-Tek HMX ST 225 (Nikon Metrology, Belgium) (scanned at: University of Cambridge)	0.0163798
Macroscelides proboscideus	NHMUK ZD 1902.9.1.18	Heather White	Skull	XT H 225 ST (Nikon Metrology, Belgium) (scanned at: NHMUK)	0.0144993
Macroscelides proboscideus	UMZC 2021.38	Robert Asher (Asher and Olbricht, 2009)	Spirit *	X-Tek HMX ST 225 (Nikon Metrology, Belgium) (scanned at: University of Cambridge)	0.0174388

Macroscelides proboscideus	NHMUK ZD 1903.1.4.10	Heather White	Skull	XT H 225 ST (Nikon Metrology, Belgium) (scanned at: NHMUK)	0.0163576
Setifer setosus	NHMUK ZD 1974.545	Heather White	Spirit	X-Tek HMX ST 225 (Nikon Metrology, Belgium) (scanned at: NHMUK)	0.0279709
Setifer setosus	NHMUK ZD 1974.554	Heather White	Spirit	X-Tek HMX ST 225 (Nikon Metrology, Belgium) (scanned at: NHMUK)	0.0261776
Setifer setosus	NHMUK ZD 1970.360	Heather White	Spirit	X-Tek HMX ST 225 (Nikon Metrology, Belgium) (scanned at: NHMUK)	0.0227106
Setifer setosus	NHMUK ZD 1855.12.26.304	Heather White	Spirit	X-Tek HMX ST 225 (Nikon Metrology, Belgium) (scanned at: NHMUK)	0.0327363
Setifer setosus	NHMUK ZD 1979.545	Heather White	Spirit	X-Tek HMX ST 225 (Nikon Metrology, Belgium) (scanned at: NHMUK)	0.0277666
Setifer setosus	NHMUK ZD 1976.273	Heather White	Spirit	X-Tek HMX ST 225 (Nikon Metrology, Belgium) (scanned at: NHMUK)	0.0528368
Setifer setosus	NHMUK ZD 1976.274	Heather White	Spirit	X-Tek HMX ST 225 (Nikon Metrology, Belgium) (scanned at: NHMUK)	0.0336312
Setifer setosus	NHMUK ZD 1974.484	Heather White	Spirit	X-Tek HMX ST 225 (Nikon Metrology, Belgium) (scanned at: NHMUK)	0.0352310
Talpa europaea	NHMUK ZD 1957.371	Heather White	Spirit	X-Tek HMX ST 225 (Nikon Metrology, Belgium) (scanned at: NHMUK)	0.0278241
Talpa europaea	NHMUK ZD 1957.368	Heather White	Spirit	X-Tek HMX ST 225 (Nikon Metrology, Belgium) (scanned at: NHMUK)	0.0269893
Talpa europaea	NHMUK ZD 1957.374	Heather White	Spirit	X-Tek HMX ST 225 (Nikon Metrology, Belgium) (scanned at: NHMUK)	0.0348750
Talpa europaea	NHMUK ZD 1957.366	Heather White	Spirit	X-Tek HMX ST 225 (Nikon Metrology, Belgium) (scanned at: NHMUK)	0.0266189
Talpa europaea	NHMUK ZD 1957.367	Heather White	Spirit	X-Tek HMX ST 225 (Nikon Metrology, Belgium) (scanned at: NHMUK)	0.0324534
Talpa europaea	NHMUK ZD 1957.370	Heather White	Spirit	X-Tek HMX ST 225 (Nikon Metrology, Belgium) (scanned at: NHMUK)	0.0314063
Talpa europaea	NHMUK ZD 2022.32	Heather White	Spirit	X-Tek HMX ST 225 (Nikon Metrology, Belgium) (scanned at: NHMUK)	0.0358665
Talpa europaea	NHMUK ZD 1989.565	Heather White	Spirit	X-Tek HMX ST 225 (Nikon Metrology, Belgium) (scanned at: NHMUK)	0.0457336

Epomops franqueti	NHMUK ZD 1969.198	Heather White	Spirit	ZEISS Xradia Versa 520 (Carl Zeiss X-ray Mircoscopy Inc., USA) (scanned at: NHMUK)	0.0185000
Epomops franqueti	NHMUK ZD 1946.283	Heather White	Spirit	X-Tek HMX ST 225 (Nikon Metrology, Belgium) (scanned at: NHMUK)	0.0240287
Epomops franqueti	NHMUK ZD 1968.353	Heather White	Spirit	X-Tek HMX ST 225 (Nikon Metrology, Belgium) (scanned at: NHMUK)	0.0451368
Epomops franqueti	NHMUK ZD 1880.7.21.4	Heather White	Spirit	X-Tek HMX ST 225 (Nikon Metrology, Belgium) (scanned at: NHMUK)	0.0338460
Epomops franqueti	NHMUK ZD 1966.3499	Heather White	Spirit	X-Tek HMX ST 225 (Nikon Metrology, Belgium) (scanned at: NHMUK)	0.0451368
Phacochoerus africanus	NHMUK ZD 1966.807a	Heather White	Spirit	X-Tek HMX ST 225 (Nikon Metrology, Belgium) (scanned at: NHMUK)	0.0829138
Phacochoerus africanus	NHMUK ZD 1966.808a	Heather White	Spirit	X-Tek HMX ST 225 (Nikon Metrology, Belgium) (scanned at: NHMUK)	0.0783939
Phacochoerus africanus	NHMUK ZD 1966.807b	Heather White	Spirit	X-Tek HMX ST 225 (Nikon Metrology, Belgium) (scanned at: NHMUK)	0.0873342
Phacochoerus africanus	NHMUK ZD 1966.811	Heather White	Skull	X-Tek HMX ST 225 (Nikon Metrology, Belgium) (scanned at: NHMUK)	0.0635098
Phacochoerus africanus	NHMUK ZD 1871.7.3.4	Heather White	Skull	XT H 225 ST (Nikon Metrology, Belgium) (scanned at: NHMUK)	0.0750196
Phacochoerus africanus	NHMUK ZD 1966.517	Heather White	Skull	X-Tek HMX ST 225 (Nikon Metrology, Belgium) (scanned at: NHMUK)	0.1185810
Phacochoerus africanus	NHMUK ZD 1966.523	Heather White	Skull	X-Tek HMX ST 225 (Nikon Metrology, Belgium) (scanned at: NHMUK)	0.1267765
Phacochoerus africanus	NHMUK ZD 1966.428	Heather White	Skull	X-Tek HMX ST 225 (Nikon Metrology, Belgium) (scanned at: NHMUK)	0.1185807
Phacochoerus africanus	NHMUK ZD 1971.2124	Heather White	Skull	X-Tek HMX ST 225 (Nikon Metrology, Belgium) (scanned at: NHMUK)	0.1185807
Phacochoerus africanus	NHMUK ZD 1971.2125	Heather White	Skull	X-Tek HMX ST 225 (Nikon Metrology, Belgium) (scanned at: NHMUK)	0.1081032
Phacochoerus africanus	NHMUK ZD 1966.425	Heather White	Skull	X-Tek HMX ST 225 (Nikon Metrology, Belgium) (scanned at: NHMUK)	0.1267761
Felis catus	NHMUK ZD 1999.132	Heather White	Spirit	XT H 225 ST (Nikon Metrology, Belgium) (scanned at: NHMUK)	0.0230142

Felis catus	NHMUK ZD 1992.184	Heather White	Spirit	XT H 225 ST (Nikon Metrology, Belgium) (scanned at: NHMUK)	0.0145483
Felis catus	NHMUK ZD 1992.178	Heather White	Spirit	XT H 225 ST (Nikon Metrology, Belgium) (scanned at: NHMUK)	0.0140769
Felis catus	NHMUK ZD 1969.508	Heather White	Spirit	XT H 225 ST (Nikon Metrology, Belgium) (scanned at: NHMUK)	0.0184898
Felis catus	NHMUK ZD 1919.7.7.3514	Heather White	Skull	XT H 225 ST (Nikon Metrology, Belgium) (scanned at: NHMUK)	0.0248608
Felis catus	NHMUK ZD 1952.10.20.2	Heather White	Skull	XT H 225 ST (Nikon Metrology, Belgium) (scanned at: NHMUK)	0.0328336
Felis catus	NHMUK ZD 1952.10.20.1	Heather White	Skull	XT H 225 ST (Nikon Metrology, Belgium) (scanned at: NHMUK)	0.0447307
Phataginus tricuspis	NHMUK ZD 1999.102	Heather White	Spirit	X-Tek HMX ST 225 (Nikon Metrology, Belgium) (scanned at: NHMUK)	0.0300847
Phataginus tricuspis	NHMUK ZD 1895.7.17.1	Heather White	Spirit	X-Tek HMX ST 225 (Nikon Metrology, Belgium) (scanned at: NHMUK)	0.0316921
Phataginus tricuspis	NHMUK ZD 1909.1.4.66	Heather White	Spirit	XT H 225 ST (Nikon Metrology, Belgium) (scanned at: NHMUK)	0.0224468
Phataginus tricuspis	NHMUK ZD 1999.93	Heather White	Spirit	X-Tek HMX ST 225 (Nikon Metrology, Belgium) (scanned at: NHMUK)	0.0499919
Phataginus tricuspis	NHMUK ZD 1991.363	Heather White	Spirit	X-Tek HMX ST 225 (Nikon Metrology, Belgium) (scanned at: NHMUK)	0.0435780
Phataginus tricuspis	NHMUK ZD 1966.3562	Heather White	Spirit	X-Tek HMX ST 225 (Nikon Metrology, Belgium) (scanned at: NHMUK)	0.0604277
Phataginus tricuspis	NHMUK ZD 1901.8.9.108	Heather White	Spirit	X-Tek HMX ST 225 (Nikon Metrology, Belgium) (scanned at: NHMUK)	0.0770590
Rattus rattus	NHMUK ZD 1979.1343	Heather White	Spirit	X-Tek HMX ST 225 (Nikon Metrology, Belgium) (scanned at: NHMUK)	0.0262976
Rattus rattus	NHMUK ZD 1970.103	Heather White	Spirit	X-Tek HMX ST 225 (Nikon Metrology, Belgium) (scanned at: NHMUK)	0.0202827
Rattus rattus	NHMUK ZD 1997.69	Heather White	Spirit	X-Tek HMX ST 225 (Nikon Metrology, Belgium) (scanned at: NHMUK)	0.0257323
Rattus rattus	NHMUK ZD 1952.1111	Heather White	Spirit	X-Tek HMX ST 225 (Nikon Metrology, Belgium) (scanned at: NHMUK)	0.0228304

Rattus rattus	NHMUK ZD 1999.14	Heather White	Spirit	X-Tek HMX ST 225 (Nikon Metrology, Belgium) (scanned at: NHMUK)	0.0304295
Mus musculus	KCL cfos_WT_1051	Heather White	Colony *	µCT 50 (Scanco Medical AG, Switzerland) (scanned at: King's College London)	0.0200000
Mus musculus	KCL cfos_WT_1044	Heather White	Colony *	µCT 50 (Scanco Medical AG, Switzerland) (scanned at: King's College London)	0.0200000
Mus musculus	KCL CD1_harddiet1	Heather White	Colony *	µCT 50 (Scanco Medical AG, Switzerland) (scanned at: King's College London)	0.0290000
Mus musculus	KCL cfos_het_1038	Heather White	Colony *	µCT 50 (Scanco Medical AG, Switzerland) (scanned at: King's College London)	0.0200000
Mus musculus	KCL CD1_harddiet2	Heather White	Colony *	µCT 50 (Scanco Medical AG, Switzerland) (scanned at: King's College London)	0.0290000
Mus musculus	KCL cfos_WT_1485	Heather White	Colony *	µCT 50 (Scanco Medical AG, Switzerland) (scanned at: King's College London)	0.0200000
Dasyprocta leporina	NHMUK ZD 1867.4.12.587	Heather White	Spirit	ZEISS Xradia Versa 520 (Carl Zeiss X-ray Mircoscopy Inc., USA) (scanned at: NHMUK)	0.0209990
Dasyprocta leporina	NHMUK ZD 1867.4.12.588	Heather White	Spirit	ZEISS Xradia Versa 520 (Carl Zeiss X-ray Mircoscopy Inc., USA) (scanned at: NHMUK)	0.0229980
Dasyprocta leporina	NHMUK ZD 2021.1	Heather White	Spirit	XT H 225 ST (Nikon Metrology, Belgium) (scanned at: NHMUK)	0.0236368
Dasyprocta leporina	NHMUK ZD 1867.4.12.472	Heather White	Spirit	XT H 225 ST (Nikon Metrology, Belgium) (scanned at: NHMUK)	0.0238189
Dasyprocta leporina	NHMUK ZD 1867.4.12.497	Heather White	Spirit	XT H 225 ST (Nikon Metrology, Belgium) (scanned at: NHMUK)	0.0236368
Dasyprocta leporina	NHMUK ZD 1997.8.13.2	Heather White	Skull	XT H 225 ST (Nikon Metrology, Belgium) (scanned at: NHMUK)	0.0356206
Dasyprocta leporina	NHMUK ZD 1912.5.11.8	Heather White	Skull	XT H 225 ST (Nikon Metrology, Belgium) (scanned at: NHMUK)	0.0418011
Dasyprocta leporina	NHMUK ZD 1952.1156	Heather White	Skull	XT H 225 ST (Nikon Metrology, Belgium) (scanned at: NHMUK)	0.0522179
Microcebus murinus	162H	Anthony Herrel (Thomas *et al.* 2016)	Colony *	XT H 225 ST (Nikon Metrology, Belgium) (scanned at: University of Bristol)	0.0142953
Microcebus murinus	948GP	Anthony Herrel (Thomas *et al.* 2016)	Colony *	XT H 225 ST (Nikon Metrology, Belgium) (scanned at: University of Bristol)	0.0268054

Microcebus murinus	143CAD	Anthony Herrel (Thomas *et al.* 2016)	Colony *	XT H 225 ST (Nikon Metrology, Belgium) (scanned at: University of Bristol)	0.0315925
Microcebus murinus	DLC 7030f	Morphosource (Open access)	Colony	XT H 225 ST (Nikon Metrology, Belgium) (scanned at: Duke University)	0.0246918
Sapajus apella	NHMUK ZD 1867.4.12.394	Heather White	Spirit	XT H 225 ST (Nikon Metrology, Belgium) (scanned at: NHMUK)	0.0252490
Sapajus apella	NHMUK ZD 1971.3174	Heather White	Spirit	XT H 225 ST (Nikon Metrology, Belgium) (scanned at: NHMUK)	0.0328336
Sapajus apella	NHMUK ZD 1928.2.9.3	Heather White	Skull	XT H 225 ST (Nikon Metrology, Belgium) (scanned at: NHMUK)	0.0374140
Sapajus apella	NHMUK ZD 1971.3177	Heather White	Spirit	XT H 225 ST (Nikon Metrology, Belgium) (scanned at: NHMUK)	0.0330085
Sapajus apella	NHMUK ZD 1903.7.25.1	Heather White	Skull	XT H 225 ST (Nikon Metrology, Belgium) (scanned at: NHMUK)	0.0374140
Sapajus apella	NHMUK ZD 1912.6.5.8	Heather White	Skull	XT H 225 ST (Nikon Metrology, Belgium) (scanned at: NHMUK)	0.0473584

*Details for the field and colony collected animals are as follows: *Monodelphis domestica*; collected from a colony following fully approved IACUC procedures (University of Illinois at Urbana-Champaign (UIUC)) details in Urban *et al.* (2017). *Phascolarctos cincereus*; Adelaide University, field collected (DENWR (SA) License Nos. K23749 /1 to 25), details in (Ramírez-Chaves *et al.* 2016). *Sminthopsis macroura*; collected from a colony (MAEC (Vic) License No. 06117), details in (Ramírez-Chaves *et al.* 2016). *Trichosurus vulpecula*; TV1-TV8 collected from a colony (DENWR (SA) License No. K23749 /1 to 20), TVAC, TVPYE, TYPYK collected from a colony (MAEC (Vic) License No. 06118), details in (Ramírez-Chaves *et al.* 2016). *Macroscelides proboscideus* (UMZC 2021.37, 2021.38, 48) details in Asher and Olbricht (2009). *Mus musculus*; collected from a colony (King's College London, License No. P8D5E2773). *Microcebus murinus*; collected from a colony (UMR7179 CNRS/MNHN, Brunoy, France; License No. D-91-114-1) details in Thomas *et al.* (2016).

Table S3. X-ray micro-Computed Tomography scanning details of NHMUK specimens. Details include: instrument, voltage (kV), current (μA), target, isotropic voxel size (mm), filter, exposure (ms), number of projections, gain (dB), frame averaging, total magnification, camera binning, source-object distance (mm), optical magnification, number of scans. When the number of scans is 2, stitching was performed following the protocol described in Butler *et al.* (2022). For full scanning details see the attached .xlsx file (Table S3).

Table S4. Specimens mirrored in Geomagic Wrap due to a damaged left-hand side of the skull.

**Species**	**Specimen Number**
Phascolarctos cinereus	Pcin3-9_15days
Trichosurus vulpecula	TV7_35days
Trichosaurus vulpecula	TVPYK_21days
Monodelphis domestica	30days
Monodelphis domestica	TMM-M-9038_adult
Sapajus apella	NHMUK ZD 1971.3174
Microcebus murinus	162H
Microcebus murinus	143CAD
Macroscelides proboscideus	NHMUK ZD 1903.1.4.10
Cyclopes didactylus	NHMUK ZD 1926.12.4.68
Dasypus novemcinctus	MNHN 1910-409b
Bradypus tridactylus	NHMUK ZD 1994.6
Bradypus tridactylus	NHMUK ZD 1867.4.12.579
Rattus rattus	NHMUK ZD 1997.69
Rattus rattus	NHMUK ZD 1979.1343
Phacochoerus africanus	NHMUK ZD 1971.2125
Phacochoerus africanus	NHMUK ZD 1871.7.3.4
Phacochoerus africanus	NHMUK ZD 1966.523
Felis catus	NHMUK ZD 1969.508
Felis catus	NHMUK ZD 1919.7.7.3514
Dasyprocta leporina	NHMUK ZD 1897.8.13.2
Dasyprocta leporina	NHMUK ZD 1867.4.12.587
Setonix brachyurus	NHMUK ZD 1906.8.1.245

Table S5. Specimen age, including discrete age (F = fetal, I = infant; J = juvenile; A = adult), and continuous age (% of adult centroid size).

Specimen	Discrete age	Continuous age
Bettongia penicillata SAM-24228_80days	I	62.569
Bettongia penicillata SAM-24230_46days	I	40.838
Bettongia penicillata SAM-24233_13-15days	F	18.187
Bettongia penicillata SAM-24238_35days	I	34.521
Bettongia penicillata SAM-24239_20days	F	23.388
Bettongia penicillata SAM-24240_56days	I	48.157
Bettongia penicillata SAM-24243_85+days	J	67.901
Bettongia penicillata SAM-24244_28days	I	32.571
Bettongia penicillata SAM-24246_60-69days	I	53.498
Bettongia penicillata SAM-24247_adult	A	100.000
Bradypus tridactylus NHMUK_ZD_1867.4.12.579	A	100.000
Bradypus tridactylus NHMUK_ZD_1989.226	I	57.244
Bradypus tridactylus NHMUK_ZD_1994.6	F	44.806
Bradypus tridactylus NHMUK_ZD_1952.1173	I	57.357
Bradypus tridactylus NHMUK_ZD_2022.31	J	68.579
Bradypus tridactylus ZMB_18834	F/N *	48.120
Bradypus tridactylus ZMB_9195	F	40.354
Sapajus apella NHMUK_ZD_1912.6.5.8	A	100.000
Sapajus apella NHMUK_ZD_1928.2.9.3	I	76.794
Sapajus apella NHMUK_ZD_1903.7.25.1	J	78.359
Sapajus apella NHMUK_ZD_1967.4.12.394	F	44.323
Sapajus apella NHMUK_ZD_1971.3174	I	61.959
Sapajus apella NHMUK_ZD_1971.3177	I	74.628
Cyclopes didactylus NHMUK_ZD_2010.105	I	57.262
Cyclopes didactylus NHMUK_ZD_1926.12.4.68	A	100.000
Cyclopes didactylus NHMUK_ZD_1855.12.36.345	J	66.253
Cyclopes didactylus ZMB_6XII1906a	F	54.274
Dasyprocta leporina NHMUK_ZD_1912.5.11.8	J	80.401
Dasyprocta leporina NHMUK_ZD_1952.1156	A	100.000
Dasyprocta leporina NHMUK_ZD_1867.4.12.472	I	51.264
Dasyprocta leporina NHMUK_ZD_1867.4.12.497	I	53.055
Dasyprocta leporina NHMUK_ZD_1867.4.12.587	F	19.230
Dasyprocta leporina NHMUK_ZD_1867.4.12.588	F	20.485
Dasyprocta leporina NHMUK_ZD_1897.8.13.2	I	71.893
Dasyprocta leporina NHMUK_ZD_2021.1	I	47.699
Dasypus novemcinctus MNHN_1901-331	I	40.817
Dasypus novemcinctus MNHN_1910-409b	I	46.797
Dasypus novemcinctus MNHN_1910-410	I	45.164
Dasypus novemcinctus z.134	A	100.000

Dasypus novemcinctus ZMB_40641	F	33.519
Dasypus novemcinctus ZMB_40651	I	42.608
Epomops franqueti NHMUK_ZD_1948.283	F	44.846
Epomops franqueti NHMUK_ZD_1966.3499	A	100.000
Epomops franqueti NHMUK_ZD_1968.353	F	49.066
Epomops franqueti NHMUK_ZD_1969.198	F	29.886
Epomops franqueti NHMUK_ZD_1880.7.21.4	I	68.085
Felis catus NHMUK_ZD_1919.7.7.3514	I	54.287
Felis catus NHMUK_ZD_1952.10.20.1	A	100.000
Felis catus NHMUK_ZD_1952.10.20.2	J	78.800
Felis catus NHMUK_ZD_1992.178	I	37.953
Felis catus NHMUK_ZD_1992.184	I	36.522
Felis catus NHMUK_ZD_1999.132	F	29.848
Felis catus NHMUK_ZD_1969.508	I	43.631
Macroscelides proboscideus NHMUK_ZD_1902.9.1.17	I	77.066
Macroscelides proboscideus NHMUK_ZD_1902.9.1.18	I	88.458
Macroscelides proboscideus NHMUK_ZD_1903.1.4.10	A	100.000
Macroscelides proboscideus UMZC_48	F	57.892
Macroscelides proboscideus UMZC_2021.37	I	88.547
Macroscelides proboscideus UMZC_2021.38	J	95.605
Phataginus tricuspis NHMUK_ZD_1901.8.9.108	A	100.000
Phataginus tricuspis NHMUK_ZD_1999.102	F	60.341
Phataginus tricuspis NHMUK_ZD_1999.93	I	72.853
Phataginus tricuspis NHMUK_ZD_1966.3562	J	90.253
Phataginus tricuspis NHMUK_ZD_1909.1.4.66	I	74.977
Phataginus tricuspis NHMUK_ZD_1991.363	I	84.389
Phataginus tricuspis NHMUK_ZD_1995.7.17.1	I	69.631
Microcebus murinus DLC_7030f	A	98.384
Microcebus murinus 162H	I	62.478
Microcebus murinus 948GP	I	86.100
Microcebus murinus 143CAD	J	100.000
Monodelphis domestica 10days	F	12.139
Monodelphis domestica 20days	F	31.160
Monodelphis domestica 22days	I	31.255
Monodelphis domestica 25days	I	36.442
Monodelphis domestica 30days	I	40.857
Monodelphis domestica 35days	I	45.332
Monodelphis domestica 6days	E	9.868
Monodelphis domestica TMM-M-7539_57days	I	59.139
Monodelphis domestica TMM-M-7542_75days	I	66.219
Monodelphis domestica TMM-M-8268_90days	J	79.684
Monodelphis domestica TMM-M-8269_48days	I	56.076
Monodelphis domestica TMM-M-9038_adult	A	100.000

Monodelphis domestica UMUT-DK150007_15days	F	25.142
Mus musculus KCL_14days_cfos_WT_1051	I	77.476
Mus musculus KCL_18days_cfos_WT_1044	I	86.263
Mus musculus KCL_25days_CD1_harddiet1	I	89.401
Mus musculus KCL_4.5moths_cfos_WT_1485	A	100.000
Mus musculus KCL_5wks_cfos_het_1038	I	94.540
Mus musculus KCL_9wk_CD1_harddiet1	J	97.972
Ornithorhynchus anatinus NHMUK_ZD_1859.5.30.4	I	72.769
Ornithorhynchus anatinus NHMUK_ZD_1890.12.4.5	A	100.000
Ornithorhynchus anatinus NHMUK_ZD_1890.12.4.6	I	91.614
Ornithorhynchus anatinus NHMUK_ZD_2022.30	J	92.310
Phacochoerus africanus NHMUK_ZD_1966.425	A	100.000
Phacochoerus africanus NHMUK_ZD_1966.428	I	70.670
Phacochoerus africanus NHMUK_ZD_1966.517	I	54.831
Phacochoerus africanus NHMUK_ZD_1966.523	I	64.417
Phacochoerus africanus NHMUK_ZD_1966.807a	F	23.022
Phacochoerus africanus NHMUK_ZD_1966.807b	F	24.520
Phacochoerus africanus NHMUK_ZD_1966.808a	F	24.118
Phacochoerus africanus NHMUK_ZD_1966.811	I	28.103
Phacochoerus africanus NHMUK_ZD_1971.2124	I	74.441
Phacochoerus africanus NHMUK_ZD_1971.2125	J	78.458
Phacochoerus africanus NHMUK_ZD_1871.7.3.4	I	40.527
Phascolarctos cinereus KoalasBottomMiddle_spec2	I	52.546
Phascolarctos cinereus Pcin3-1_57days	I	18.225
Phascolarctos cinereus Pcin3-13_51days	I	15.060
Phascolarctos cinereus Pcin3-5_46days	I	14.758
Phascolarctos cinereus Pcin3-6_25days	I	9.943
Phascolarctos cinereus Pcin3-7_21days	I	8.767
Phascolarctos cinereus Pcin3-8_30days	I	12.278
Phascolarctos cinereus Pcin3-9_15days	F	7.458
Phascolarctos cinereus PcinLge02_100+days	A	100.000
Phascolarctos cinereus PcinLge03_100+days	J	94.477
Phascolarctos cinereus PcinLge05_60+days	I	86.457
Rattus rattus NHMUK_ZD_1997.69	I	79.812
Rattus rattus NHMUK_ZD_1999.14	A	100.000
Rattus rattus NHMUK_ZD_1952.1111	J	81.151
Rattus rattus NHMUK_ZD_1970.103	I	68.724
Rattus rattus NHMUK_ZD_1979.1343	I	66.526
Setifer setosus NHMUK_ZD_1974.484	A	100.000
Setifer setosus NHMUK_ZD_1855.12.26.304	I	60.909
Setifer setosus NHMUK_ZD_1970.360	I	61.236
Setifer setosus NHMUK_ZD_1974.545	I	58.932
Setifer setosus NHMUK_ZD_1974.554	I	51.068

Setifer setosus NHMUK_ZD_1976.273	I	90.376
Setifer setosus NHMUK_ZD_1976.274	J	93.869
Setifer setosus NHMUK_ZD_1979.545	I	90.803
Setonix brachyurus NHMUK_ZD_1989.580	F	27.281
Setonix brachyurus NHMUK_ZD_1989.581	I	29.312
Setonix brachyurus NHMUK_ZD_1989.582	I	36.608
Setonix brachyurus NHMUK_ZD_1989.583	I	39.113
Setonix brachyurus NHMUK_ZD_1989.584	I	42.336
Setonix brachyurus NHMUK_ZD_1989.585	I	48.588
Setonix brachyurus NHMUK_ZD_1989.586	I	50.627
Setonix brachyurus NHMUK_ZD_1989.587	I	53.481
Setonix brachyurus NHMUK_ZD_1989.588	I	57.143
Setonix brachyurus NHMUK_ZD_1989.589	I	54.262
Setonix brachyurus NHMUK_ZD_1989.590	J	59.827
Setonix brachyurus NHMUK_ZD_1989.591	I	58.336
Setonix brachyurus NHMUK_ZD_1906.8.1.245	A	100.000
Sminthopsis macroura Smac54dPYA_No44_08_54days	I	76.278
Sminthopsis macroura Smac64_9_09_64days	I	80.610
Sminthopsis macroura SmacAdult33_08_adult	A	100.000
Sminthopsis macroura Smacd19_19days	F	25.920
Sminthopsis macroura Smacd22_22days	I	29.068
Sminthopsis macroura Smacd31_31days	I	40.828
Sminthopsis macroura SmacPYE74d_No14_08_74days	J	85.005
Talpa europaea NHMUK_ZD_2022.32	J	94.254
Talpa europaea NHMUK_ZD_1989.565	A	100.000
Talpa europaea NHMUK_ZD_1957.366	I	79.558
Talpa europaea NHMUK_ZD_1957.367	I	91.194
Talpa europaea NHMUK_ZD_1957.368	I	64.112
Talpa europaea NHMUK_ZD_1957.370	I	81.395
Talpa europaea NHMUK_ZD_1957.371	I	64.176
Talpa europaea NHMUK_ZD_1957.374	I	71.531
Trichosurus vulpecula TV1_adult	J	91.470
Trichosurus vulpecula TV2_100days	A	100.000
Trichosurus vulpecula TV3_85days	I	80.916
Trichosurus vulpecula TV6_35-42days	I	42.548
Trichosurus vulpecula TV7_35days	I	36.441
Trichosurus vulpecula TV8_68days	I	68.562
Trichosurus vulpecula TVAC_14days	F	20.913
Trichosurus vulpecula TVPYE_15days	F	27.876
Trichosurus vulpecula TVPYK_21days	I	31.659

* Neonate specimen grouped as fetal specimen.

Table S6. Landmark descriptions used across all specimens within the dataset, based on Coombs *et al.* 2022 and Goswami *et al.* 2022. The three common midline landmarks are highlighted in red. Specimen-specific midline landmark details can be found in Table S9. Landmark positions are demonstrated in Figure 2B-D.

Landmark Number	Landmark Description	Region
1	Nasal: anterior most point (midline)	Nasal
2	Nasal: lateral anterior extreme	Nasal
3	Nasal: posterior lateral corner	Nasal
4	Nasal: posterior point	Nasal
5	Tip of the rostrum, anterior dorsal side, anterior midline of tooth row (usually premaxilla)	Premaxilla
6	Premaxilla: anterior dorsal extreme	Premaxilla
7	Premaxilla: posterior dorsal extreme	Premaxilla
8	Premaxilla: posterior lateral ventral extreme	Premaxilla
9	Maxilla: anterior lateral ventral extreme	Maxilla
10	Maxilla: anterior dorsal medial most point (the nasal-premaxilla-maxilla suture)	Maxilla
11	Maxilla: posterior medial most point (usually the nasal-frontal-maxilla suture)	Maxilla
12	Maxilla: dorsal posterior most point to include the lacrimal (usually on the orbit)	Maxilla
13	Maxilla: jugal-maxilla orbit suture	Maxilla
14	Maxilla: posterior ventral lateral extreme, at the jugal-maxilla ventral suture	Maxilla
15	Maxilla: posterior tooth row lateral extreme	Maxilla
16	Jugal: anterior dorsal extreme	Jugal
17	Jugal: anterior ventral extreme	Jugal
18	Jugal: posterior ventral extreme	Jugal
19	Frontal: anterior medial most point (midline)	Frontal
20	Frontal: posterior medial most point (midline)	Frontal
21	Frontal: lateral posterior extreme, at the posterior lateral parietal suture	Frontal
22	Postorbital process (anterior on crest)	Frontal
23	Frontal: anterior lateral extreme (on orbit)	Frontal
24	Frontal: anterior dorsal extreme (usually at the frontal-premaxilla-nasal suture)	Frontal
25	Parietal: anterior medial most point (midline)	Parietal
26	Parietal: posterior medial most point (midline)	Parietal
27	Parietal: posterior lateral extreme (usually the squamosal-occipital suture)	Parietal
28	Parietal: anterior lateral point, at the parietal-squamosal suture (on vault)	Parietal
29	Parietal: dorsal anterior lateral extreme, at the parietal-frontal suture	Parietal
30	Squamosal: dorsal anterior squamosal suture with parietal (or maybe alisphenoid/frontal)	Squamosal
31	Squamosal: medial anterior zygomatic vault junction	Squamosal
32	Squamosal: anterior dorsal jugal-squamosal suture	Squamosal
33	Squamosal: posterior lateral ventral jugal-squamosal suture	Squamosal

34	Squamosal: anterior medial most point of the mandibular articular process	Squamosal
35	Squamosal: posterior lateral extreme of the mandibular articular process	Squamosal
36	Squamosal: posterior lateral ventral extreme, at the squamosal-occipital suture	Squamosal
37	Squamosal: posterior medial dorsal most point, at the squamosal-parietal-occipital suture	Squamosal
38	Interparietal: dorsal anterior most point (midline)	Interparietal
39	Interparietal: posterior most point (midline)	Interparietal
40	Interparietal: posterior lateral extreme, at the lateral interparietal-occipital-parietal suture	Interparietal
41	Posterior margin of the skull roof	Supraoccipital
42	Supraoccipital: anterior medial most point (usually the parietal-occipital suture)	Supraoccipital
43	Dorsal margin of the foramen magnum (midline)	Supraoccipital
44	Dorsal medial occipital condyle	Occipital
45	Dorsal lateral occipital condyle	Occipital
46	Tip of the paraoccipital process (lateral tip)	Paraoccipital
47	Paraoccipital: lateral ventral extreme of the occipital (not the process)	Paraoccipital
48	Supraoccipital: lateral dorsal extreme	Supraoccipital
49	Ventral medial occipital condyle	Occipital
50	Ventral lateral occipital condyle	Occipital
51	Ventral margin of the foramen magnum (basion, midline)	Occipital
52	Basioccipital: anterior medial most point (midline)	Basioccipital
53	Basioccipital: lateral anterior extreme	Basioccipital
54	Basisphenoid: anterior medial most point, just posterior to the pterygoids and palate (midline)	Basisphenoid
55	Basisphenoid: lateral anterior extreme	Basisphenoid
56	Basisphenoid: lateral posterior extreme	Basisphenoid
57	Basisphenoid: posterior medial most point (midline)	Basisphenoid
58	Palate: ventral posterior medial most point (midline)	Palate
59	Palate: ventral anterior medial most point (midline)	Palate
60	Palate: pal-pterygoid suture	Palate
61	Palate: lateral posterior extreme of the pal-max suture	Palate
62	Pterygoid posterior	Pterygoid
63	Posterior pterygoid on the ventral surface of skull base	Pterygoid
64	Maxilla: ventral medial posterior suture (midline)	Maxilla
65	Maxilla: ventral medial anterior suture (midline)	Maxilla
66	Maxilla: ventral anterior lateral extreme	Maxilla
67	Premaxilla: ventral medial posterior suture (midline)	Ventral premaxilla
68	Premaxilla: anterior most point of palatal surface immediately posterior to tooth row (midline)	Ventral premaxilla
69	Premaxilla: ventral posterior lateral extreme	Ventral premaxilla

Table S7. Raw ectocranial suture closure scores (0-5), where suture numbers across the top (1-31) correspond to the suture order in Table S5 and specimen number down the side (1-165) corresponds to the specimen order in Appendix Three: Table S3.

	**Suture number**																														
**Specimen number**	**1**	**2**	**3**	**4**	**5**	**6**	**7**	**8**	**9**	**10**	**11**	**12**	**13**	**14**	**15**	**16**	**17**	**18**	**19**	**20**	**21**	**22**	**23**	**24**	**25**	**26**	**27**	**28**	**29**	**30**	**31**
**1**	2	3	2	1	1	1	1	1	1	3	1	1	2	4	1	1	1	1	1	2	1	1	3	3	3	2	1	5	1	1	5
**2**	1	1	1	1	1	1	1	1	1	3	1	1	1	3	1	2	1	1	1	1	1	1	2	1	2	1	1	1	1	1	3
**3**	1	1	1	1	1	1	1	1	1	1	1	1	1	1	1	1	1	1	1	1	1	1	1	1	1	1	1	1	1	1	1
**4**	1	1	1	1	1	1	1	1	1	2	1	1	1	2	1	1	1	1	1	1	1	1	1	1	2	1	1	1	1	1	3
**5**	1	1	1	1	1	1	1	1	1	1	1	1	1	1	1	1	1	1	1	1	1	1	1	1	1	1	1	1	1	1	1
**6**	1	3	1	1	1	1	1	1	1	3	1	1	1	3	1	1	1	1	1	1	1	1	2	2	2	1	1	3	1	1	5
**7**	4	4	2	2	1	1	1	1	1	4	2	2	2	4	1	2	1	1	1	2	3	2	3	4	3	3	2	5	2	1	5
**8**	1	1	1	1	1	1	1	1	1	2	1	1	1	2	1	1	1	1	1	1	1	1	1	1	1	1	1	1	1	1	3
**9**	1	3	1	2	1	1	1	1	1	3	1	1	1	4	1	1	1	1	1	2	1	1	3	3	3	2	1	4	1	1	5
**10**	5	5	5	5	5	5	5	5	5	5	4	4	5	5	5	5	5	5	3	5	5	5	5	5	5	5	5	5	5	5	5
**11**	5	5	5	4	4	5	5	5	5	5	5	4	5	5	5	5	4	1	5	5	5	1	1	5	5	5	5	4	1	5	5
**12**	3	3	3	2	1	1	1	1	1	2	1	1	1	3	1	5	4	1	3	1	2	1	1	2	3	4	3	1	1	3	3
**13**	1	1	1	1	1	1	1	1	1	1	1	1	1	1	1	5	1	1	1	1	1	1	1	1	1	1	1	1	1	2	1
**14**	4	3	3	3	1	1	1	1	2	1	1	1	1	2	1	5	1	1	1	1	2	1	1	3	1	3	2	2	1	2	3
**15**	4	4	4	4	1	1	1	2	3	3	1	4	1	4	3	5	1	1	1	2	2	1	1	1	2	3	2	1	1	3	3
**16**	1	3	1	1	1	1	1	1	1	1	1	1	1	1	1	5	1	1	1	1	1	1	1	1	2	4	1	1	1	2	3
**17**	1	1	1	1	1	1	1	1	1	1	1	1	1	1	1	5	1	1	1	1	1	1	1	1	1	1	1	1	1	1	1
**18**	5	4	2	3	1	1	4	4	5	4	2	3	1	4	4	3	3	4	3	3	4	5	4	5	4	4	5	4	4	3	3
**19**	5	2	3	1	1	4	1	3	2	2	2	2	1	4	1	3	4	2	3	2	2	4	3	5	4	3	5	4	4	3	2
**20**	5	3	2	3	1	1	4	2	5	2	2	2	1	4	2	2	2	2	2	2	3	4	5	5	4	4	5	3	3	3	3
**21**	1	1	1	1	1	1	1	1	1	1	1	1	1	1	1	1	1	1	1	1	1	1	1	1	1	1	1	2	1	1	1
**22**	3	1	1	1	1	1	1	1	1	1	2	1	1	1	1	1	1	1	1	2	2	2	1	1	1	2	1	2	1	2	1

**23**	5	3	2	2	1	1	3	2	3	3	2	2	1	2	1	2	3	3	2	2	2	3	2	5	2	3	4	3	3	3	2
**24**	2	2	1	1	1	1	1	2	2	1	1	2	1	1	1	5	3	1	3	2	4	1	1	4	1	3	2	4	1	4	4
**25**	5	5	5	5	3	5	5	5	5	5	2	5	1	5	5	5	4	1	4	4	5	1	1	4	1	4	3	5	1	4	5
**26**	4	2	1	2	1	1	1	3	3	1	1	4	1	1	1	5	4	1	5	3	5	1	1	4	1	2	1	5	1	4	5
**27**	2	2	2	1	1	1	1	1	1	1	1	1	1	1	1	5	2	1	5	2	3	1	1	4	1	4	1	4	1	1	3
**28**	5	4	5	5	5	1	4	3	3	3	4	4	5	5	5	4	3	3	5	3	4	4	4	5	4	3	4	3	4	3	4
**29**	4	5	5	5	5	1	5	3	5	4	5	4	4	5	5	4	4	5	5	4	4	3	4	3	4	3	5	3	5	5	4
**30**	2	2	1	1	1	1	1	1	1	2	3	1	2	2	1	3	1	1	5	1	1	1	1	1	2	2	1	1	1	2	2
**31**	2	2	1	1	1	1	1	1	1	2	1	2	2	2	1	2	1	1	3	1	2	1	1	1	2	2	1	1	1	2	2
**32**	1	1	1	1	1	1	1	1	1	2	1	1	2	1	1	4	1	1	1	1	1	1	1	2	3	1	1	1	1	1	1
**33**	1	2	1	1	1	1	1	1	1	3	1	1	3	2	1	4	1	1	1	2	1	1	1	1	3	2	1	1	1	1	1
**34**	5	5	5	4	1	1	3	3	1	4	3	3	4	4	5	4	2	3	5	3	2	2	3	4	3	3	2	2	5	2	3
**35**	1	1	1	1	1	1	1	1	1	2	1	2	3	2	1	5	1	1	4	2	1	1	1	1	3	2	1	1	1	1	1
**36**	1	3	2	2	1	1	1	1	1	1	1	1	1	2	1	2	1	3	1	3	2	2	3	5	1	2	2	2	1	2	1
**37**	1	3	3	2	1	2	1	3	1	2	1	4	1	3	1	2	2	2	1	3	3	2	2	4	2	3	2	2	1	3	3
**38**	1	2	2	3	2	3	1	3	1	2	3	2	1	2	1	3	1	3	1	4	5	3	3	3	3	3	2	2	1	3	5
**39**	5	5	5	5	5	5	4	5	5	5	5	5	5	5	5	5	5	5	5	5	5	5	5	5	5	5	5	5	5	5	5
**40**	1	1	1	1	1	1	1	1	1	1	1	1	1	1	1	1	1	1	1	1	2	1	1	1	2	2	1	1	1	2	1
**41**	1	1	1	1	1	1	1	1	1	4	2	2	1	1	1	3	5	5	5	3	3	5	5	5	3	3	3	3	2	5	3
**42**	1	1	1	1	1	1	1	1	1	1	1	1	1	3	1	1	1	1	1	2	1	2	1	2	1	1	1	1	1	2	1
**43**	5	5	5	5	5	3	5	3	5	4	5	4	1	5	3	1	5	1	5	5	3	3	1	5	5	5	5	5	5	3	4
**44**	4	4	3	3	2	1	1	2	1	2	1	1	1	4	2	1	3	1	2	3	1	3	4	5	2	3	3	4	2	1	2
**45**	1	2	1	1	1	1	1	1	1	1	1	1	1	3	1	1	1	1	1	2	1	3	1	1	1	1	1	1	1	1	1
**46**	5	5	4	4	1	2	4	3	4	4	1	3	1	4	1	1	4	1	3	3	1	3	5	5	3	3	4	5	4	2	2
**47**	4	4	4	4	3	3	3	3	3	3	3	2	3	4	1	5	5	4	1	4	3	3	3	3	5	3	4	1	4	3	2
**48**	5	4	5	5	5	3	5	3	5	5	3	4	5	5	5	5	4	4	2	5	5	3	5	4	4	3	5	1	4	5	5
**49**	3	4	1	1	2	1	2	1	5	3	2	2	5	3	1	1	1	3	1	3	3	4	2	4	4	2	3	1	1	3	2

**50**	1	2	1	1	1	1	1	1	1	2	1	1	1	2	1	1	1	1	1	2	1	1	1	1	1	1	1	1	1	1	1
**51**	1	2	1	1	1	1	1	1	1	2	1	2	1	2	1	1	1	1	1	1	1	1	1	1	1	1	1	1	1	1	1
**52**	1	1	1	1	1	1	1	1	1	1	1	1	1	1	1	1	1	1	1	1	1	1	1	1	1	1	1	1	1	1	1
**53**	1	3	1	1	1	1	1	1	1	3	1	2	1	2	1	1	1	1	1	2	1	1	1	1	1	2	1	1	1	2	2
**54**	4	4	3	4	5	1	3	1	1	4	3	3	5	5	1	5	4	3	4	2	1	2	4	5	5	3	2	3	2	3	4
**55**	4	4	5	5	5	1	5	5	1	5	5	5	5	5	1	5	5	4	5	4	3	4	5	5	5	5	5	5	2	5	5
**56**	5	5	5	5	5	5	5	5	5	5	4	5	5	5	5	4	5	4	5	4	3	5	5	5	4	3	5	5	4	5	5
**57**	2	2	1	1	4	1	1	1	1	3	2	2	1	3	1	5	5	1	5	3	1	2	4	4	3	3	2	2	1	1	4
**58**	4	4	2	3	5	1	3	3	1	4	4	4	5	5	1	5	5	4	5	4	1	4	5	5	5	5	4	5	2	4	4
**59**	2	4	1	5	5	4	5	5	5	4	2	4	1	5	5	5	4	3	3	4	3	2	2	3	3	3	5	5	1	5	5
**60**	4	4	5	5	3	3	4	5	5	4	4	3	2	1	5	5	2	1	1	3	1	1	3	5	1	4	2	2	1	2	4
**61**	1	2	1	1	1	1	1	2	5	1	1	3	1	1	1	5	5	1	5	3	1	2	3	3	1	2	1	1	1	2	2
**62**	4	3	3	3	1	1	1	1	5	3	3	2	1	2	1	4	1	1	1	3	1	1	2	2	1	1	1	1	1	1	3
**63**	2	1	2	3	2	2	2	2	5	3	3	3	1	1	1	5	5	1	4	4	1	1	3	4	1	1	1	1	1	3	4
**64**	1	2	1	1	1	1	1	1	5	1	1	2	1	1	1	3	1	1	1	3	1	1	1	1	1	1	1	1	1	1	2
**65**	3	1	1	2	2	2	1	2	5	2	1	2	2	2	1	5	5	1	2	3	1	1	2	5	1	1	1	1	1	2	4
**66**	1	1	1	1	1	1	1	1	5	1	1	2	1	1	1	5	1	2	2	2	1	1	2	2	1	1	1	1	1	1	3
**67**	5	5	5	4	4	4	4	4	4	5	5	5	4	4	5	5	3	5	5	4	4	5	5	5	5	5	5	5	4	5	4
**68**	1	1	1	1	1	1	1	1	1	2	1	1	1	1	1	3	2	1	1	2	1	1	2	2	2	2	2	2	1	1	1
**69**	3	3	2	1	1	1	4	4	3	4	3	3	3	4	1	4	3	1	1	3	3	2	4	4	3	3	3	5	3	2	1
**70**	5	5	5	5	1	3	5	4	5	5	4	4	5	4	5	5	5	4	5	4	5	5	5	5	4	5	5	5	5	3	3
**71**	1	1	1	1	1	1	1	1	1	1	1	1	1	1	1	1	1	1	1	1	1	2	2	1	2	2	1	3	1	1	1
**72**	1	3	1	1	1	1	1	1	1	3	1	1	1	1	1	5	2	2	1	2	1	1	4	2	2	3	4	2	1	2	3
**73**	1	1	1	1	1	1	1	1	1	2	1	1	1	1	1	5	1	2	1	2	1	1	4	4	2	2	3	2	1	1	1
**74**	3	3	1	2	1	1	1	1	1	3	1	2	1	2	1	5	1	1	1	2	2	3	5	4	2	3	5	2	2	2	3
**75**	4	3	1	2	1	1	1	1	1	2	1	2	1	3	1	5	3	2	3	2	3	3	5	5	3	4	5	4	3	2	4
**76**	3	3	2	3	1	1	1	2	1	2	1	3	1	3	1	5	2	2	3	2	3	2	4	5	3	4	5	5	4	2	3

**77**	1	1	1	1	1	1	1	1	1	1	1	1	1	1	1	1	1	1	1	1	1	2	1	1	2	1	1	1	1	1	1
**78**	5	5	5	5	1	4	4	3	5	5	3	2	5	5	1	5	5	4	4	3	4	4	5	5	5	4	5	5	4	5	5
**79**	5	5	5	5	2	4	5	4	5	5	3	3	5	5	1	5	5	5	5	5	4	5	5	5	5	5	5	5	5	5	5
**80**	3	4	2	4	1	1	5	3	1	5	3	3	5	4	1	2	1	2	2	4	2	2	2	1	4	4	3	4	4	5	5
**81**	3	3	1	3	1	1	1	1	1	4	1	2	1	3	1	2	1	1	2	2	3	2	4	2	3	3	4	4	1	2	2
**82**	4	5	5	5	5	5	5	3	5	5	3	4	5	5	5	4	4	3	5	4	5	3	3	1	4	4	5	3	4	5	5
**83**	1	1	1	1	1	1	1	1	1	1	1	1	1	1	1	5	2	2	1	1	1	2	1	1	3	2	2	1	1	1	1
**84**	2	2	1	1	1	1	1	1	1	2	1	1	2	2	1	3	1	1	1	2	2	2	2	4	2	3	1	1	1	3	1
**85**	3	3	2	2	1	1	3	1	2	4	2	2	5	3	1	3	2	2	1	3	3	3	2	4	3	4	2	1	1	2	2
**86**	4	4	3	3	1	1	3	1	5	4	2	2	5	4	1	5	1	2	2	4	2	2	3	5	4	5	3	5	1	3	3
**87**	4	3	2	2	1	3	5	1	5	4	3	3	4	3	1	4	3	4	3	4	4	3	3	4	4	4	3	3	1	3	3
**88**	5	3	4	3	1	3	5	1	5	4	3	3	5	4	2	4	3	3	3	4	4	3	3	5	4	4	3	3	2	3	3
**89**	5	4	3	3	3	3	5	1	5	4	3	3	4	4	1	4	4	3	3	5	5	4	3	5	4	5	5	5	2	4	3
**90**	5	4	5	4	1	1	1	4	1	5	3	4	1	5	3	1	5	5	5	4	1	5	5	3	5	5	5	5	5	1	2
**91**	5	5	5	5	5	5	5	5	5	5	3	4	1	5	5	1	5	5	5	5	5	5	5	4	5	5	5	5	5	5	5
**92**	5	5	5	5	5	5	1	5	3	5	3	4	1	5	5	1	5	5	5	4	3	3	5	3	5	5	5	5	5	5	5
**93**	5	5	5	5	5	5	4	5	5	5	3	4	1	5	5	1	5	4	5	5	3	3	4	3	5	5	5	5	5	5	5
**94**	5	5	5	5	5	5	5	5	4	5	5	4	5	5	5	1	2	4	3	4	5	3	2	4	5	5	5	5	2	5	4
**95**	4	4	5	5	5	2	5	3	4	5	4	4	5	5	5	1	3	2	4	4	5	3	3	3	4	3	4	3	2	5	4
**96**	5	4	5	5	5	1	2	5	2	5	3	5	5	5	5	2	3	3	5	3	5	2	2	4	4	4	4	5	3	5	3
**97**	5	4	5	4	5	4	2	5	2	5	3	5	5	4	5	1	3	3	3	4	5	3	2	4	4	5	5	5	2	5	3
**98**	1	1	1	1	1	1	1	1	1	1	1	2	1	1	1	1	1	1	1	1	1	1	1	1	1	2	1	2	1	1	2
**99**	2	2	4	1	1	1	1	2	1	4	2	2	2	2	1	1	1	1	1	2	2	1	1	3	2	2	1	2	1	2	2
**100**	1	1	1	1	1	2	1	2	1	2	1	2	2	1	1	1	1	1	1	1	1	1	1	1	1	2	1	2	1	1	2
**101**	4	3	4	2	1	5	4	4	1	4	3	4	4	3	1	2	1	1	2	2	3	2	4	4	3	2	2	3	2	3	3
**102**	4	3	4	5	5	2	5	3	4	4	4	4	4	5	5	1	2	3	3	4	5	2	3	3	4	3	4	2	2	5	4
**103**	4	4	5	5	5	2	5	3	1	3	4	3	5	5	5	1	1	4	3	3	5	1	2	1	4	3	4	2	2	4	4

**104**	5	5	5	5	1	3	5	5	2	5	3	5	5	5	5	4	3	3	1	2	5	1	5	5	3	4	4	3	3	5	3
**105**	2	1	1	1	1	1	1	1	1	2	1	1	1	1	1	5	3	3	5	3	5	3	1	3	4	4	3	1	1	1	4
**106**	1	1	1	1	1	1	1	1	1	1	1	1	1	1	1	1	1	1	1	1	1	1	1	1	2	1	1	1	1	1	1
**107**	1	1	1	1	1	1	1	1	1	1	1	1	1	1	1	1	1	1	1	1	1	1	1	1	2	2	2	2	1	1	1
**108**	1	1	1	1	1	1	1	1	1	1	1	1	1	1	1	1	1	1	1	1	1	1	1	1	1	1	1	1	1	1	1
**109**	1	1	1	1	1	1	1	1	1	1	1	1	1	1	1	1	1	1	1	1	1	1	1	1	1	1	1	1	1	1	1
**110**	1	1	1	1	1	1	1	1	1	1	1	1	1	1	1	1	1	1	1	1	1	1	1	1	1	1	1	1	1	1	1
**111**	1	1	1	1	1	1	1	1	1	1	1	1	1	1	1	1	1	1	1	1	1	1	1	1	1	1	1	1	1	1	1
**112**	1	1	1	1	1	1	1	1	1	1	1	1	1	1	1	1	1	1	1	1	1	1	1	1	1	1	1	1	1	1	1
**113**	5	5	5	5	1	1	1	5	1	5	4	5	3	5	1	5	5	3	5	3	5	3	1	3	5	4	4	3	4	3	5
**114**	5	4	5	5	1	1	1	3	1	4	3	5	2	5	1	5	5	3	5	3	5	2	3	4	5	4	4	4	4	2	5
**115**	1	1	1	1	1	1	1	1	1	2	1	1	1	1	1	1	2	2	2	1	1	1	1	1	2	2	1	1	1	1	5
**116**	3	3	1	2	1	1	3	1	3	4	2	1	2	4	2	4	4	3	4	3	3	3	2	5	3	1	4	1	1	3	1
**117**	5	3	4	2	1	2	5	3	5	5	4	3	4	5	2	5	3	4	3	4	4	4	3	5	4	4	3	2	1	4	5
**118**	4	4	2	3	1	1	1	2	1	3	4	2	4	4	1	4	4	4	4	4	3	3	3	5	2	2	5	3	1	4	3
**119**	3	3	3	2	1	1	1	1	1	3	2	2	3	3	2	4	4	3	5	3	3	3	3	5	4	1	2	1	1	3	1
**120**	2	3	1	1	1	1	1	1	1	2	1	1	1	2	1	4	1	1	2	2	2	1	2	4	3	1	2	1	1	2	1
**121**	5	5	5	5	3	5	5	2	5	5	3	5	5	5	5	5	5	4	5	5	5	5	5	5	1	4	5	5	1	5	3
**122**	2	4	1	4	1	1	1	1	1	4	2	4	4	4	1	5	5	4	4	4	3	1	5	4	1	4	5	4	1	3	3
**123**	4	4	3	4	3	1	1	2	1	5	2	4	4	4	1	5	4	2	3	3	3	1	4	4	1	4	5	3	1	3	3
**124**	2	4	1	4	3	1	1	1	1	4	2	3	5	4	1	3	4	2	3	3	1	1	3	4	1	4	5	3	1	1	1
**125**	1	4	1	3	1	1	1	1	1	2	2	2	2	4	1	4	1	2	1	3	1	1	2	4	1	4	2	4	1	1	1
**126**	4	4	2	2	3	1	5	1	5	4	2	4	5	3	1	3	3	2	5	3	3	3	5	5	1	4	5	5	1	4	3
**127**	5	4	2	3	3	2	5	1	5	5	2	5	5	4	3	3	4	2	3	3	4	2	2	5	1	4	5	5	1	4	3
**128**	4	5	3	5	3	3	4	1	5	5	2	5	5	5	1	4	5	2	4	3	3	3	4	5	1	4	4	3	1	4	4
**129**	1	1	1	1	1	1	1	1	1	1	1	1	1	1	1	3	1	1	1	1	1	1	1	1	1	3	1	2	1	1	5
**130**	1	1	1	1	1	1	1	1	1	3	1	1	1	1	1	3	1	1	1	1	1	1	2	2	1	3	1	1	1	1	5

**131**	2	2	1	1	1	1	1	1	1	4	1	1	1	1	1	3	1	1	1	1	1	1	2	2	2	3	1	3	1	1	5
**132**	4	2	1	1	1	1	1	1	1	5	1	3	1	4	1	3	1	1	3	1	3	1	5	3	3	4	2	5	2	1	5
**133**	3	2	1	3	1	1	1	1	1	5	1	1	1	4	1	3	1	1	1	1	1	1	5	3	3	3	2	5	1	1	5
**134**	4	3	2	3	1	1	1	1	1	4	1	2	1	4	1	3	1	1	2	1	3	1	5	4	4	4	2	5	1	3	5
**135**	4	4	3	3	1	1	1	1	1	4	1	2	1	4	1	3	1	1	1	1	3	1	5	3	3	3	2	5	1	3	5
**136**	4	4	3	3	1	1	1	1	1	4	1	2	1	5	1	4	1	1	2	1	3	1	4	4	4	3	2	5	1	4	5
**137**	4	4	4	3	1	1	1	1	1	4	1	2	1	5	1	4	3	1	1	1	3	1	4	5	4	3	4	5	2	4	5
**138**	4	4	3	3	1	1	1	1	1	4	1	2	1	4	1	4	2	1	1	3	3	1	4	4	4	3	4	5	1	4	5
**139**	5	4	4	4	1	1	1	3	1	5	1	2	1	5	1	3	1	1	1	4	3	1	5	4	4	4	4	5	3	5	5
**140**	5	4	3	3	1	1	1	3	1	4	1	2	1	4	1	3	1	1	2	3	3	1	5	5	4	3	4	5	1	4	5
**141**	5	5	5	5	1	1	5	5	5	5	5	4	5	5	5	3	1	3	4	5	5	4	5	1	5	4	5	5	2	5	5
**142**	3	3	1	1	1	1	1	3	1	3	1	3	4	3	1	5	1	1	3	1	4	3	4	4	4	4	3	4	4	1	1
**143**	3	3	1	1	1	1	1	3	1	3	1	3	4	4	1	5	1	2	3	1	4	3	5	5	4	4	3	4	4	1	1
**144**	3	4	1	2	3	2	4	3	5	3	1	3	5	5	3	5	3	4	3	1	5	5	5	5	5	4	5	5	4	3	3
**145**	1	1	1	1	1	1	1	1	1	1	1	1	1	1	1	1	1	1	1	1	1	1	1	1	1	1	1	1	1	1	1
**146**	1	1	1	1	1	1	1	1	1	1	1	1	1	1	1	1	1	1	1	1	1	1	1	1	1	1	1	1	1	1	1
**147**	1	2	1	1	1	1	1	1	1	1	1	1	1	1	1	5	1	1	3	1	1	2	3	1	2	3	1	1	1	1	1
**148**	3	4	1	2	1	1	4	3	4	4	1	3	5	5	4	5	3	3	3	1	5	3	5	5	5	4	4	4	4	3	1
**149**	4	4	1	1	2	2	1	1	1	4	1	3	3	2	2	4	5	1	5	2	3	2	3	5	5	5	5	3	1	1	3
**150**	5	5	4	5	3	5	4	2	3	5	2	5	5	4	4	4	5	4	5	5	5	5	5	5	5	5	5	5	5	3	3
**151**	3	4	1	1	1	1	1	1	1	2	2	3	1	2	1	4	5	1	2	3	3	2	3	4	5	3	5	1	1	1	1
**152**	4	4	1	1	3	3	1	1	1	3	2	3	3	2	2	4	5	2	4	3	4	2	4	5	5	4	5	3	3	2	2
**153**	1	3	1	1	1	1	1	1	1	1	1	1	1	2	1	5	3	1	1	3	2	1	3	1	5	3	2	1	1	1	1
**154**	4	5	1	1	3	2	1	1	1	4	2	1	1	3	1	4	5	2	4	4	4	2	5	5	5	5	5	2	5	1	4
**155**	1	3	1	1	1	1	1	1	1	1	1	1	1	2	1	4	5	1	2	3	2	1	3	5	2	2	2	1	1	1	1
**156**	2	3	1	1	1	1	1	1	1	2	1	1	1	2	1	5	5	1	2	3	3	2	5	5	5	3	5	1	1	1	1
**157**	2	4	1	3	1	1	1	5	1	5	1	3	1	4	1	3	2	2	2	3	4	2	4	4	4	4	3	5	1	3	5

**158**	2	4	2	4	1	1	1	4	1	4	1	3	1	4	1	2	2	2	1	3	4	1	4	4	4	4	4	4	1	3	5
**159**	1	2	1	2	1	1	1	1	1	2	1	1	1	2	1	1	2	1	1	1	2	1	4	2	2	2	3	2	1	1	5
**160**	1	1	1	1	1	1	1	1	1	1	1	1	1	1	1	1	1	1	1	1	1	1	1	1	1	1	1	1	1	1	5
**161**	1	1	1	1	1	1	1	1	1	1	1	1	1	1	1	1	1	1	1	1	1	1	1	1	1	1	1	1	1	1	5
**162**	1	1	1	1	1	1	1	1	1	2	1	1	1	1	1	1	2	1	1	1	2	1	3	1	1	2	1	1	1	1	5
**163**	1	1	1	1	1	1	1	1	1	1	1	1	1	1	1	1	1	1	1	1	1	1	1	1	1	1	1	1	1	1	5
**164**	1	1	1	1	1	1	1	1	1	1	1	1	1	1	1	1	1	1	1	1	1	1	1	1	1	1	1	1	1	1	5
**165**	1	1	1	1	1	1	1	1	1	1	1	1	1	1	1	1	1	1	1	1	1	1	1	1	1	1	1	1	1	1	5

Table S8. Developmental origin of each suture, reported as neural crest, mesoderm, or the boundary between the two. Full references can be found in reference list of this this thesis.

Suture	Developmental Origin	Reference
Interfrontal	Neural crest	Morriss-Kay (2001)
Fronto-parietal	Boundary	Morriss-Kay (2001)
Interparietal	Mesoderm	Morriss-Kay (2001)
Supraoccipito-parietal	Mesoderm *	Morriss-Kay (2001); Teng *et al.* (2019)
Basispheno-presphenoid	Neural crest	McBratney-Owen *et al.* (2008)
Basispheno-basioccipital	Boundary	McBratney-Owen *et al.* (2008)
Exoccipito-basioccipital	Mesoderm	McBratney-Owen *et al.* (2008)
Exoccipito-squamosal	Boundary	Morriss-Kay (2001); Jiang *et al.* (2002)
Exoccipito-supraoccipital	Mesoderm	Teng *et al.* (2019)
Alispheno-squamosal	Neural crest	Morriss-Kay (2001); Jiang *et al.* (2002)
Alispheno-orbitosphenoid	Boundary	Morriss-Kay (2001); Noden and Francis-West (2006); McBratney-Owen *et al.* (2008)
Orbitospheno-frontal	Boundary	Morriss-Kay (2001); Noden and Francis-West (2006); McBratney-Owen *et al.* (2008)
Fronto-squamosal	Neural crest	Morriss-Kay (2001); Jiang *et al.* (2002)
Parieto-squamosal	Boundary	Morriss-Kay (2001); Jiang *et al.* (2002)
Supraoccipito-squamosal	Boundary	Morriss-Kay (2001); Jiang *et al.* (2002); Teng *et al.* (2019)
Interpremaxillary	Neural crest	Jiang *et al.* (2002)
Intermaxillary	Neural crest	Jiang *et al.* (2002)
Premaxillo-maxillary (ventral)	Neural crest	Jiang *et al.* (2002)
Interpalatine	Neural crest	Chai *et al.* (2000)
Maxillo-palatine	Neural crest	Chai *et al.* (2000); Jiang *et al.* (2002)
Pterygo-palatine	Neural crest	Chai *et al.* (2000)
Premaxillo-maxillary (facial)	Neural crest	Jiang *et al.* (2002)
Premaxillo-nasal	Neural crest	Morriss-Kay (2001); Jiang *et al.* (2002)
Internasal	Neural crest	Morriss-Kay (2001)
Maxillo-jugal	Neural crest	Jiang *et al.* (2002)
Maxillo-lacrimal	Neural crest	Jiang *et al.* (2002)
Naso-frontal	Neural crest	Morriss-Kay (2001)
Lacrimo-frontal	Neural crest	Morriss-Kay (2001)
Jugo-squamosal	Neural crest	Morriss-Kay (2001); Jiang *et al.* (2002)
Palato-alisphenoid	Neural crest	Chai *et al.* (2000); Morriss-Kay (2001)
Palato-orbitosphenoid	Boundary	Chai *et al.* (2000); Noden and Francis-West (2006); McBratney-Owen *et al.* (2008)

* Considered mesoderm as most species don’t have an interparietal, which is a dual origin bone (Koyabu *et al.* 2012).

Table S9. Suture closure scores (%) (SCS) calculated from ectocranial analysis across all adult specimens, across the full dataset, across all adult placental mammal specimens, and across all adult marsupial mammal specimens

Suture	SCS (%) (adults)	SCS (%) (all specimens)	SCS (%) (placentals)	SCS (%) (marsupials)
Interfrontal	89.773	45.606	95.000	75.000
Fronto-parietal	89.773	47.424	88.333	91.667
Interparietal	82.955	31.667	86.667	70.833
Supraoccipito-parietal	84.091	35.303	83.333	83.333
Basispheno-presphenoid	59.091	19.394	63.333	41.667
Basispheno-basioccipital	60.227	16.212	66.667	37.500
Exoccipito-basioccipital	84.091	26.212	91.667	62.500
Exoccipito-squamosal	71.591	24.697	66.667	79.167
Exoccipito-supraoccipital	86.364	28.333	93.333	66.667
Alispheno-squamosal	90.909	49.091	91.667	87.500
Alispheno-orbitosphenoid	63.636	22.273	70.000	50.000
Orbitospheno-frontal	76.136	33.636	78.333	70.833
Fronto-squamosal	67.045	30.303	68.333	75.000
Parieto-squamosal	88.636	48.182	85.000	95.833
Supraoccipito-squamosal	76.136	19.848	81.667	58.333
Interpremaxillary	72.727	52.424	76.667	75.000
Intermaxillary	68.182	36.061	70.000	58.333
Premaxillo-maxillary (ventral)	61.364	24.091	60.000	58.333
Interpalatine	71.591	36.364	73.333	62.500
Maxillo-palatine	77.273	36.667	81.667	62.500
Pterygo-palatine	84.091	37.727	78.333	95.833
Premaxillo-maxillary (facial)	63.636	26.212	61.667	62.500
Premaxillo-nasal	65.909	44.091	61.667	70.833
Internasal	79.545	53.030	90.000	54.167
Maxillo-jugal	77.273	43.939	70.000	91.667
Maxillo-lacrimal	80.682	46.818	80.000	79.167
Naso-frontal	87.500	44.545	85.000	91.667
Lacrimo-frontal	76.136	43.939	73.333	79.167
Jugo-squamosal	53.409	21.212	48.333	58.333
Palato-alisphenoid	78.409	36.212	78.333	75.000
Palato-orbitosphenoid	82.955	50.758	78.333	91.667

Table S10. Total suture closure score (%) for each specimen, calculated from ectocranial analysis by combining all sutures for each specimen. For discrete age: fetal (F), infant (I), juvenile (J), adult (A).

**Specimen**	**Discrete age**	**Total suture closure score (%)**
Bettongia penicillata SAM-24228_80days	I	20.968
Bettongia penicillata SAM-24230_46days	I	7.258
Bettongia penicillata SAM-24233_13-15days	F	0
Bettongia penicillata SAM-24238_35days	I	4.032
Bettongia penicillata SAM-24239_20days	F	0
Bettongia penicillata SAM-24240_56days	I	12.097
Bettongia penicillata SAM-24243_85+days	J	33.065
Bettongia penicillata SAM-24244_28days	I	3.226
Bettongia penicillata SAM-24246_60-69days	I	18.548
Bettongia penicillata SAM-24247_adult	A	96.774
Bradypus tridactylus NHMUK_ZD_1867.4.12.579	A	83.065
Bradypus tridactylus NHMUK_ZD_1989.226	I	25.806
Bradypus tridactylus NHMUK_ZD_1994.6	F	4.032
Bradypus tridactylus NHMUK_ZD_1952.1173	I	20.161
Bradypus tridactylus NHMUK_ZD_2022.31	J	31.452
Bradypus tridactylus ZMB_18834	F	10.484
Bradypus tridactylus ZMB_9195	F	3.226
Sapajus apella NHMUK_ZD_1912.6.5.8	A	62.097
Sapajus apella NHMUK_ZD_1928.2.9.3	I	45.161
Sapajus apella NHMUK_ZD_1903.7.25.1	J	48.387
Sapajus apella NHMUK_ZD_1967.4.12.394	F	0.806
Sapajus apella NHMUK_ZD_1971.3174	I	7.258
Sapajus apella NHMUK_ZD_1971.3177	I	37.097
Cyclopes didactylus NHMUK_ZD_2010.105	I	25.806
Cyclopes didactylus NHMUK_ZD_1926.12.4.68	A	70.161
Cyclopes didactylus NHMUK_ZD_1855.12.36.345	J	35.484
Cyclopes didactylus ZMB_6XII1906a	F	20.968
Dasyprocta leporina NHMUK_ZD_1912.5.11.8	J	72.581
Dasyprocta leporina NHMUK_ZD_1952.1156	A	79.839
Dasyprocta leporina NHMUK_ZD_1867.4.12.472	I	13.710
Dasyprocta leporina NHMUK_ZD_1867.4.12.497	I	11.290
Dasyprocta leporina NHMUK_ZD_1867.4.12.587	F	6.452
Dasyprocta leporina NHMUK_ZD_1867.4.12.588	F	10.484
Dasyprocta leporina NHMUK_ZD_1897.8.13.2	I	54.839
Dasyprocta leporina NHMUK_ZD_2021.1	I	12.903
Dasypus novemcinctus MNHN_1901-331	I	17.742
Dasypus novemcinctus MNHN_1910-409b	I	28.226
Dasypus novemcinctus MNHN_1910-410	I	34.677

Dasypus novemcinctus z.134	A	99.194
Dasypus novemcinctus ZMB_40641	F	3.226
Dasypus novemcinctus ZMB_40651	I	40.323
Epomops franqueti NHMUK_ZD_1948.283	F	4.839
Epomops franqueti NHMUK_ZD_1966.3499	A	75.000
Epomops franqueti NHMUK_ZD_1968.353	F	33.065
Epomops franqueti NHMUK_ZD_1969.198	F	4.839
Epomops franqueti NHMUK_ZD_1880.7.21.4	I	51.613
Felis catus NHMUK_ZD_1919.7.7.3514	I	55.645
Felis catus NHMUK_ZD_1952.10.20.1	A	80.645
Felis catus NHMUK_ZD_1952.10.20.2	J	34.677
Felis catus NHMUK_ZD_1992.178	I	3.226
Felis catus NHMUK_ZD_1992.184	I	3.226
Felis catus NHMUK_ZD_1999.132	F	0
Felis catus NHMUK_ZD_1969.508	I	8.065
Macroscelides proboscideus NHMUK_ZD_1902.9.1.17	I	54.839
Macroscelides proboscideus NHMUK_ZD_1902.9.1.18	I	82.258
Macroscelides proboscideus NHMUK_ZD_1903.1.4.10	A	91.935
Macroscelides proboscideus UMZC_48	F	33.065
Macroscelides proboscideus UMZC_2021.37	I	68.548
Macroscelides proboscideus UMZC_2021.38	J	66.129
Phataginus tricuspis NHMUK_ZD_1901.8.9.108	A	51.613
Phataginus tricuspis NHMUK_ZD_1999.102	F	24.194
Phataginus tricuspis NHMUK_ZD_1999.93	I	22.581
Phataginus tricuspis NHMUK_ZD_1966.3562	J	33.871
Phataginus tricuspis NHMUK_ZD_1909.1.4.66	I	8.871
Phataginus tricuspis NHMUK_ZD_1991.363	I	27.419
Phataginus tricuspis NHMUK_ZD_1995.7.17.1	I	12.903
Microcebus murinus DLC_7030f	A	88.710
Microcebus murinus 162H	I	8.871
Microcebus murinus 948GP	I	43.548
Microcebus murinus 143CAD	J	86.290
Monodelphis domestica 10days	F	4.839
Monodelphis domestica 20days	F	20.161
Monodelphis domestica 22days	I	14.516
Monodelphis domestica 25days	I	29.032
Monodelphis domestica 30days	I	40.323
Monodelphis domestica 35days	I	41.129
Monodelphis domestica 6days	E	1.613
Monodelphis domestica TMM-M-7539_57days	I	79.839
Monodelphis domestica TMM-M-7542_75days	I	88.710
Monodelphis domestica TMM-M-8268_90days	J	49.194
Monodelphis domestica TMM-M-8269_48days	I	27.419

Monodelphis domestica TMM-M-9038_adult	A	80.645
Monodelphis domestica UMUT-DK150007_15days	F	8.871
Mus musculus KCL_14days_cfos_WT_1051	I	15.323
Mus musculus KCL_18days_cfos_WT_1044	I	33.871
Mus musculus KCL_25days_CD1_harddiet1	I	50.000
Mus musculus KCL_4.5moths_cfos_WT_1485	A	53.226
Mus musculus KCL_5wks_cfos_het_1038	I	59.677
Mus musculus KCL_9wk_CD1_harddiet1	J	67.742
Ornithorhynchus anatinus NHMUK_ZD_1859.5.30.4	I	62.903
Ornithorhynchus anatinus NHMUK_ZD_1890.12.4.5	A	90.323
Ornithorhynchus anatinus NHMUK_ZD_1890.12.4.6	I	80.645
Ornithorhynchus anatinus NHMUK_ZD_2022.30	J	83.871
Phacochoerus africanus NHMUK_ZD_1966.425	A	81.452
Phacochoerus africanus NHMUK_ZD_1966.428	I	70.161
Phacochoerus africanus NHMUK_ZD_1966.517	I	70.968
Phacochoerus africanus NHMUK_ZD_1966.523	I	71.774
Phacochoerus africanus NHMUK_ZD_1966.807a	F	3.226
Phacochoerus africanus NHMUK_ZD_1966.807b	F	17.742
Phacochoerus africanus NHMUK_ZD_1966.808a	F	6.452
Phacochoerus africanus NHMUK_ZD_1966.811	I	44.355
Phacochoerus africanus NHMUK_ZD_1971.2124	I	64.516
Phacochoerus africanus NHMUK_ZD_1971.2125	J	58.065
Phacochoerus africanus NHMUK_ZD_1871.7.3.4	I	70.161
Phascolarctos cinereus KoalasBottomMiddle_spec2	I	28.226
Phascolarctos cinereus Pcin3-1_57days	I	0.806
Phascolarctos cinereus Pcin3-13_51days	I	3.226
Phascolarctos cinereus Pcin3-5_46days	I	0
Phascolarctos cinereus Pcin3-6_25days	I	0
Phascolarctos cinereus Pcin3-7_21days	I	0
Phascolarctos cinereus Pcin3-8_30days	I	0
Phascolarctos cinereus Pcin3-9_15days	F	0
Phascolarctos cinereus PcinLge02_100+days	A	66.129
Phascolarctos cinereus PcinLge03_100+days	J	62.903
Phascolarctos cinereus PcinLge05_60+days	I	8.065
Rattus rattus NHMUK_ZD_1997.69	I	37.903
Rattus rattus NHMUK_ZD_1999.14	A	64.516
Rattus rattus NHMUK_ZD_1952.1111	J	48.387
Rattus rattus NHMUK_ZD_1970.103	I	37.097
Rattus rattus NHMUK_ZD_1979.1343	I	15.323
Setifer setosus NHMUK_ZD_1974.484	A	84.677
Setifer setosus NHMUK_ZD_1855.12.26.304	I	48.387
Setifer setosus NHMUK_ZD_1970.360	I	49.194
Setifer setosus NHMUK_ZD_1974.545	I	37.097

Setifer setosus NHMUK_ZD_1974.554	I	23.387
Setifer setosus NHMUK_ZD_1976.273	I	56.452
Setifer setosus NHMUK_ZD_1976.274	J	59.677
Setifer setosus NHMUK_ZD_1979.545	I	63.710
Setonix brachyurus NHMUK_ZD_1989.580	F	7.258
Setonix brachyurus NHMUK_ZD_1989.581	I	9.677
Setonix brachyurus NHMUK_ZD_1989.582	I	14.516
Setonix brachyurus NHMUK_ZD_1989.583	I	32.258
Setonix brachyurus NHMUK_ZD_1989.584	I	26.613
Setonix brachyurus NHMUK_ZD_1989.585	I	35.484
Setonix brachyurus NHMUK_ZD_1989.586	I	33.871
Setonix brachyurus NHMUK_ZD_1989.587	I	37.903
Setonix brachyurus NHMUK_ZD_1989.588	I	42.742
Setonix brachyurus NHMUK_ZD_1989.589	I	40.323
Setonix brachyurus NHMUK_ZD_1989.590	J	49.194
Setonix brachyurus NHMUK_ZD_1989.591	I	43.548
Setonix brachyurus NHMUK_ZD_1906.8.1.245	A	78.226
Sminthopsis macroura Smac54dPYA_No44_08_54days	I	37.097
Sminthopsis macroura Smac64_9_09_64days	I	40.323
Sminthopsis macroura SmacAdult33_08_adult	A	65.323
Sminthopsis macroura Smacd19_19days	F	0
Sminthopsis macroura Smacd22_22days	I	0
Sminthopsis macroura Smacd31_31days	I	10.484
Sminthopsis macroura SmacPYE74d_No14_08_74days	J	58.065
Talpa europaea NHMUK_ZD_2022.32	J	43.548
Talpa europaea NHMUK_ZD_1989.565	A	83.871
Talpa europaea NHMUK_ZD_1957.366	I	30.645
Talpa europaea NHMUK_ZD_1957.367	I	48.387
Talpa europaea NHMUK_ZD_1957.368	I	16.935
Talpa europaea NHMUK_ZD_1957.370	I	50.000
Talpa europaea NHMUK_ZD_1957.371	I	18.548
Talpa europaea NHMUK_ZD_1957.374	I	29.839
Trichosurus vulpecula TV1_adult	J	43.548
Trichosurus vulpecula TV2_100days	A	41.129
Trichosurus vulpecula TV3_85days	I	15.323
Trichosurus vulpecula TV6_35-42days	I	3.226
Trichosurus vulpecula TV7_35days	I	3.226
Trichosurus vulpecula TV8_68days	I	8.065
Trichosurus vulpecula TVAC_14days	F	3.226
Trichosurus vulpecula TVPYE_15days	F	3.226
Trichosurus vulpecula TVPYK_21days	I	3.226

Table S11. Reconstructed ancestral total suture closure scores (%), calculated from ectocranial analysis using the adult specimens only. Ancestral nodes correspond with the nodes on the phylogeny in Appendix Three: Figure S1.

Species/Ancestral node	Reconstructed total suture closure score (%)
Bettongia penicillata	96.774
Bradypus tridactylus	83.065
Cyclopes didactylus	70.161
Dasyprocta leporina	79.839
Dasypus novemcinctus	99.194
Epomops franqueti	75.000
Felis catus	80.645
Macroscelides proboscideus	91.935
Microcebus murinus	88.710
Monodelphis domestica	80.645
Mus musculus	53.226
Ornithorhynchus anatinus	90.326
Phacochoerus africanus	81.452
Phascolarctos cinereus	66.129
Phataginus tricuspis	51.613
Rattus rattus	64.516
Sapajus apella	62.097
Setifer setosus	84.677
Setonix brachyurus	78.226
Sminthopsis macroura	65.323
Talpa europaea	83.871
Trichosurus vulpecula	41.129
Ancestral mammal (node 23)	79.272
Ancestral therian mammal (node 24)	78.225
Ancestral eutherian (node 25)	78.691
Ancestral Laurasiatheria (node 27)	76.862
Ancestral Euarchontoglires (node 31)	75.533
Ancestral Afrotheria (node 36)	81.315
Ancestral Xenarthra (node 37)	81.762
Ancestral metatherian (node 39)	72.258

Table S12. Raw suture closure scores for interfrontal, frontoparietal, and interparietal sutures, from ectocranial and cross-sectional scoring. Ectocranial analysis scores for each suture, and the raw closure score across the three sutures. A score of 15 indicates complete fusion across the three sutures, whilst 0 indicates patency of all three sutures. Cross-sectional fused score is similarly combined for the three sutures. A score of 9 for cross-sectional fusion indicates complete fusion across all three sutures at all three sample points along the suture length, whilst 0 indicates complete patency for all three sutures.

Species	Ectocranial (Interfrontal)	Ectocranial (Fronto-parietal)	Ectocranial (Interparietal)	Ectocranial three suture closure score	Cross-sectional fusion score
Bettongia penicillata	5	5	5	15	0
Bradypus tridactylus	5	5	5	15	8
Sapajus apella	5	4	2	11	3
Cyclopes didactylus	5	5	5	15	3
Dasyprocta leporina	4	5	5	14	2
Epomops franqueti	5	5	5	15	6
Felis catus	5	4	5	14	0
Macroscelides proboscideus	5	5	5	15	0
Phataginus tricuspis	4	4	5	13	0
Microcebus murinus	5	5	5	15	4
Monodelphis domestica	4	5	5	14	1
Mus musculus	4	3	2	9	0
Ornithorhynchus anatinus	5	5	5	15	8
Phacochoerus aethiopicus	5	5	5	15	9
Phascolarctos cincereus	5	5	5	15	0
Rattus rattus	5	3	4	12	1
Setifer setosus	5	5	5	15	0
Setonix brachyurus	5	5	5	15	1
Sminthopsis macroura	3	4	1	8	0
Talpa europaea	5	5	4	14	7

Trichosaurus vulpecula	2	4	2	8	0

Table S13. Pattern of fusion across the Krogman regions (1930) for each species (n=22), based on ectocranial analysis. Number of species (N), maximum suture closure score (%) calculated for the adult specimen of each species (MSC). Kendall's τ indicates the correlation between the species pattern of fusion and the Krogman pattern. Spearman's ρ indicates the correlation between total suture closure score and skull size (logCS). Where * indicates $p < 0.05$.

Species	Fusion Pattern	N	MSC (%)	Kendall's τ	Spearman's ρ
B. penicillata	Vault (1), Cranial base (1), Facial (1), Cranio-facial (1), Circum-meatal (1), Palate (1)	10	96.77	-0.18	0.99 *
B. tridactylus	Circum-meatal (1), Cranial base (2), Vault (3), Palate (4), Cranio-facial (5), Facial (6)	7	83.06	0.73 *	0.96 *
C. didactylus	Vault (1), Cranial base (2), Circum-meatal (3), Palate (3), Cranio-facial (5), Facial (6)	4	70.16	-0.55	1 *
D. leporina	Vault (1), Circum-meatal (2), Cranio-facial (3), Palate (4), Cranial base (5), Facial (6)	8	79.84	0.83 *	0.93 *
D. novemcinctus	Vault (1), Circum-meatal (1), Palate (1), Facial (1), Cranio-facial (1), Cranial base (6)	6	99.19	0.60	0.77
E. franqueti	Vault (1), Cranio-facial (2), Cranial base (3), Facial (4), Circum-meatal (5), Palate (6)	5	75.00	-0.11	0.97 *
F. catus	Vault (1), Circum-meatal (2), Cranial base (3), Palate (4), Cranio-facial (5), Facial (6)	7	80.65	0.20	0.95 *
M. proboscideus	Vault (1), Cranial base (1), Circum-meatal (3), Cranio-facial (4), Facial (5), Palate (6)	6	91.94	0.55	0.77
M. murinus	Facial (1), Vault (2), Circum-meatal (3), Cranio-facial (4), Palate (5), Cranial base (6)	4	88.71	0.47	0.80
M. domestica	Vault (1), Cranial base (2), Circum-meatal (3), Cranio-facial (4), Palate (5), Facial (6)	13	80.65	-0.07	0.93 *
M. musculus	Palate (1), Facial (2), Cranial base (3), Circum-meatal (3), Vault (5), Cranio-facial (6)	6	53.23	0.73 *	0.83 *
O. anatinus	Vault (1), Cranial base (1), Cranio-facial (1), Facial (4), Palate (5), Circum-meatal (6)	4	90.32	-0.28	1 *
P. africanus	Vault (1), Circum-meatal (2), Cranial base (3), Cranio-facial (4), Facial (5), Palate (6)	11	81.45	0	0.75 *
P. cinereus	Vault (1), Palate (2), Circum-meatal (3), Cranio-facial (4), Facial (5), Cranial base (6)	11	66.13	0.73 *	0.93 *
P. tricuspis	Vault (1), Cranial base (2), Circum-meatal (3), Facial (4), Cranio-facial (5), Palate (6)	7	51.61	0.47	0.68
R. rattus	Facial (1), Circum-meatal (2), Palate (2), Vault (4), Cranial base (5), Cranio-facial (6)	5	64.51	-0.14	1 *
S. apella	Facial (1), Cranio-facial (2), Vault (3), Palate (4), Cranial base (5), Circum-meatal (5)	6	70.16	0.55	1 *
S. setosus	Vault (1), Palate (2), Circum-meatal (3), Cranial base (4), Facial (4), Cranio-facial (6)	8	84.68	0.33	0.98 *
S. brachyurus	Vault (1), Circum-meatal (2), Cranio-facial (3), Facial (4), Palate (5), Cranial base (6)	13	78.23	-1.00 *	0.99 *
S. macroura	Facial (1), Cranio-facial (2), Palate (3), Cranial base (4), Circum-meatal (5), Vault (6)	7	65.32	-0.33	0.99 *
T. europaea	Facial (1), Palate (2), Vault (3), Cranio-facial (4), Circum-meatal (5), Cranial base (6)	8	83.87	-0.36	0.90 *
T. vulpecula	Facial (1), Cranio-facial (1), Vault (3), Circum-meatal (4), Palate (4), Cranial base (6)	9	41.13	0.73 *	0.89 *

Table S14. Suture closure scores (%) for each suture for each species, calculated from ectocranial analysis by combining all specimens of the same species.

Suture	*Bettongia penicillata*	*Bradypus tridactylus*	*Sapajus apella*	*Cyclopes didactylus*	*Dasyprocta leporina*	*Dasypus novemcinctus*	*Epomops franqueti*	*Felis catus*	*Macroscelides proboscideus*
Interfrontal	20.000	42.857	75.000	56.250	40.625	16.667	55.000	32.143	62.500
Fronto-parietal	32.500	46.429	33.333	43.750	43.750	37.500	60.000	46.429	70.833
Interparietal	15.000	39.286	20.833	31.250	37.500	33.333	45.000	25.000	45.833
Supraoccipito-parietal	15.000	32.143	20.833	31.250	34.375	33.333	45.000	25.000	70.833
Basispheno-presphenoid	10.000	10.714	0	12.500	25.000	20.833	25.000	25.000	95.833
Basispheno-basioccipital	10.000	14.286	12.500	25.000	0	29.167	15.000	14.286	29.167
Exoccipito-basioccipital	10.000	14.286	33.333	25.000	28.125	12.500	35.000	25.000	66.667
Exoccipito-squamosal	10.000	17.857	29.167	43.750	18.750	33.333	25.000	14.286	58.333
Exoccipito-supraoccipital	10.000	25.000	45.833	43.750	18.750	16.667	35.000	35.714	33.333
Alispheno-squamosal	42.500	25.000	29.167	25.000	43.750	37.500	35.000	42.857	79.167
Alispheno-orbitosphenoid	10.000	14.286	20.833	6.250	34.375	29.167	20.000	17.857	58.333
Orbitospheno-frontal	10.000	21.429	20.833	50.000	31.250	37.500	25.000	25.000	70.833
Fronto-squamosal	15.000	14.286	0	0	53.125	16.667	0	35.714	66.667
Parieto-squamosal	47.500	35.714	41.667	25.000	46.875	33.333	70.000	42.857	91.667
Supraoccipito-squamosal	10.000	21.429	16.667	25.000	37.500	16.667	15.000	14.286	33.333
Interpremaxillary	15.000	100.000	25.000	100.000	68.750	41.667	0	28.571	95.833
Intermaxillary	10.000	21.429	33.333	56.250	18.750	37.500	45.000	25.000	91.667
Premaxillo-maxillary (ventral)	10.000	0	29.167	0	25.000	54.167	0	28.571	54.167
Interpalatine	5.000	21.429	25.000	81.250	65.625	33.333	35.000	3.571	87.500
Maxillo-palatine	17.500	17.857	25.000	43.750	28.125	54.167	50.000	39.286	62.500
Pterygo-palatine	15.000	25.000	33.333	81.250	25.000	58.333	10.000	28.571	25.000
Premaxillo-maxillary (facial)	12.500	0	54.167	0	18.750	50.000	45.000	25.000	54.167
Premaxillo-nasal	30.000	0	41.667	0	25.000	54.167	35.000	25.000	79.167

Internasal	30.000	25.000	66.667	75.000	31.250	70.833	65.000	28.571	87.500
Maxillo-jugal	32.500	28.571	41.667	0	50.000	41.667	35.000	35.714	79.167
Maxillo-lacrimal	20.000	50.000	45.833	56.250	31.250	50.000	40.000	21.429	66.667
Naso-frontal	12.500	28.571	62.500	18.750	25.000	37.500	45.000	32.143	70.833
Lacrimo-frontal	42.500	14.286	50.000	87.500	15.625	37.500	55.000	0	79.167
Jugo-squamosal	12.500	0	41.667	0	34.375	20.833	40.000	21.429	25.000
Palato-alisphenoid	10.000	39.286	37.500	56.250	28.125	58.333	20.000	32.143	70.833
Palato-orbitosphenoid	65.000	42.857	25.000	81.250	31.250	50.000	25.000	25.000	87.500

Table S14 cont.

Suture	*Phataginus tricuspis*	*Microcebus murinus*	*Monodelphis domestica*	*Mus musculus*	*Ornithorhynchus anatinus*	*Phacochoerus africanus*	*Phascolarctos cinereus*	*Rattus rattus*
Interfrontal	32.143	62.500	42.308	70.833	100.000	65.909	20.455	60.000
Fronto-parietal	25.000	62.500	48.077	54.167	93.750	56.818	15.909	55.000
Interparietal	25.000	56.250	26.923	37.500	100.000	75.000	18.182	30.000
Supraoccipito-parietal	32.143	43.750	40.385	33.333	93.750	63.636	18.182	25.000
Basispheno-presphenoid	14.286	18.750	9.615	8.333	75.000	54.545	0	0
Basispheno-basioccipital	14.286	31.250	19.231	25.000	75.000	38.636	0	5.000
Exoccipito-basioccipital	14.286	62.500	28.846	66.667	43.750	56.818	0	30.000
Exoccipito-squamosal	25.000	56.250	19.231	0	93.750	61.364	13.636	15.000
Exoccipito-supraoccipital	100.000	56.250	23.077	70.833	62.500	27.273	0	30.000
Alispheno-squamosal	28.571	75.000	50.000	66.667	100.000	72.727	20.455	60.000
Alispheno-orbitosphenoid	25.000	56.250	15.385	33.333	50.000	50.000	11.364	40.000
Orbitospheno-frontal	35.714	56.250	25.000	33.333	75.000	65.909	18.182	20.000
Fronto-squamosal	7.143	56.250	30.769	79.167	0	72.727	6.818	45.000
Parieto-squamosal	7.143	56.250	42.308	58.333	100.000	68.182	18.182	65.000
Supraoccipito-squamosal	14.286	50.000	7.692	4.1667	87.500	63.636	0	15.000
Interpremaxillary	89.286	81.250	71.154	70.833	0	11.364	27.273	80.000
Intermaxillary	46.429	56.250	30.769	33.333	100.000	22.727	25.000	55.000
Premaxillo-maxillary (ventral)	3.571	43.750	28.846	37.500	93.750	34.091	15.909	50.000
Interpalatine	32.143	50.000	32.692	29.167	100.000	36.364	29.545	65.000
Maxillo-palatine	50.000	56.250	34.615	66.667	87.500	43.182	13.636	55.000
Pterygo-palatine	0	56.250	34.615	58.333	50.000	70.455	27.273	50.000
Premaxillo-maxillary (facial)	3.571	56.250	36.538	45.833	75.000	20.455	11.364	45.000
Premaxillo-nasal	32.143	75.000	61.538	41.667	93.750	34.091	4.545	40.000
Internasal	53.571	75.000	46.154	87.500	56.250	50.000	15.909	95.000

Maxillo-jugal	0	62.500	51.923	62.500	100.000	54.545	31.818	55.000
Maxillo-lacrimal	14.286	68.750	53.846	79.167	100.000	54.545	25.000	20.000
Naso-frontal	3.571	68.750	67.308	45.833	100.000	54.545	20.455	55.000
Lacrimo-frontal	3.571	81.250	53.846	50.000	100.000	52.273	13.636	15.000
Jugo-squamosal	0	56.250	36.538	8.333	100.000	22.727	13.636	0
Palato-alisphenoid	17.857	43.750	40.385	50.000	75.000	68.182	6.818	55.000
Palato-orbitosphenoid	53.571	31.250	50.000	37.500	81.250	52.273	34.091	30.000

Table S14 cont.

Suture	*Setifer setosus*	*Setonix brachyurus*	*Sminthopsis macroura*	*Talpa europaea*	*Trichosurus vulpecula*
Interfrontal	59.375	63.462	28.571	50.000	5.556
Fronto.parietal	81.250	51.923	39.286	71.875	19.444
Interparietal	31.250	36.538	0	9.375	2.778
Supraoccipito-parietal	68.750	40.385	7.143	12.500	16.667
Basispheno-presphenoid	37.500	0	7.143	21.875	0
Basispheno-basioccipital	21.875	0	3.571	25.000	0
Exoccipito-basioccipital	46.875	7.692	21.429	9.375	0
Exoccipito-squamosal	6.250	15.385	28.571	3.125	19.444
Exoccipito-supraoccipital	50.000	7.692	25.000	6.250	0
Alispheno-squamosal	81.250	75.000	32.143	43.750	25.000
Alispheno-orbitosphenoid	28.125	7.692	0	12.500	0
Orbitospheno-frontal	75.000	23.077	28.571	31.250	11.111
Fronto-squamosal	84.375	7.692	50.000	25.000	0
Parieto-squamosal	78.125	65.385	46.429	34.375	19.444
Supraoccipito-squamosal	18.750	7.692	17.857	15.625	0
Interpremaxillary	75.000	55.769	71.429	81.250	8.333
Intermaxillary	71.875	5.769	14.286	93.750	11.111
Premaxillo-maxillary (ventral)	37.500	3.846	21.429	15.625	5.556
Interpalatine	62.500	15.385	35.714	53.125	2.778
Maxillo-palatine	59.375	21.154	0	56.250	11.111
Pterygo-palatine	46.875	38.462	50.000	56.250	22.222
Premaxillo-maxillary (facial)	28.125	5.769	39.286	28.125	2.778
Premaxillo-nasal	68.750	75.000	60.714	71.875	30.556
Internasal	87.500	53.846	53.571	84.375	19.444

Maxillo-jugal	0	55.769	53.571	90.625	19.444
Maxillo-lacrimal	75.000	57.692	50.000	68.750	22.222
Naso-frontal	87.500	40.385	39.286	81.250	19.444
Lacrimo-frontal	75.000	82.692	46.429	28.125	22.222
Jugo-squamosal	0	9.615	42.857	31.250	0
Palato-alisphenoid	53.125	46.154	14.286	9.375	11.111
Palato-orbitosphenoid	40.625	100.000	7.143	25.000	100

Table S15. Number of open and fused sutures analysed in the cross-section, out of a possible nine suture locations for each species (n=21).

Species	Open	Fused/Partially fused
Ornithorhynchus anatinus	2	7
Monodelphis domestica	8	1
Sminthopsis macroura	9	0
Phascolarctos cinereus	9	0
Trichosurus vulpecula	9	0
Setonix brachyurus	8	1
Bettongia penicillata	9	0
Bradypus tridactylus	1	8
Cyclopes didactylus	6	3
Macroscelides proboscideus	9	0
Setifer setosus	9	0
Talpa europaea	2	7
Epomops franqueti	3	6
Phacochoerus africanus	0	9
Felis catus	9	0
Phataginus tricuspis	9	0
Rattus rattus	8	1
Mus musculus	9	0
Dasyprocta leporina	7	2
Microcebus murinus	5	4
Sapajus apella	6	3

Table S16. Raw cross-sectional suture closure status for the three sutures (interfrontal, sagittal, coronal) at three positions for the 21 species. Suture closure status: open (0), partially fused (½), closed (1).

Species	Interfrontal (anterior)	Interfrontal (middle)	Interfrontal (posterior)	Coronal (medial)	Coronal (middle)	Coronal (lateral)	Sagittal (anterior)	Sagittal (middle)	Sagittal (lateral)
Ornithorhynchus anatinus	1	1	1	0	1	1	1	1	1
Monodelphis domestica	0	0	0	0	0	0	0	0	1
Sminthopsis macroura	0	0	0	0	0	0	0	0	0
Phascolarctos cinereus	0	0	0	0	0	0	0	0	0
Trichosurus vulpecula	0	0	0	0	0	0	0	0	0
Setonix brachyurus	0	0	0	0	0	0	0	0	1
Bettongia penicillata	0	0	0	0	0	0	0	0	0
Bradypus tridactylus	0	½	1	1	1	1	1	1	1
Cyclopes didactylus	½	1	½	0	0	0	0	0	0
Macroscelides proboscideus	0	0	0	0	0	0	0	0	0
Setifer setosus	0	0	0	0	0	0	0	0	0
Talpa europaea	1	1	1	1	1	1	1	0	0
Epomops franqueti	0	0	0	1	1	1	1	1	1
Phacochoerus africanus	1	1	1	1	1	1	1	1	1
Felis catus	0	0	0	0	0	0	0	0	0
Phataginus tricuspis	0	0	0	0	0	0	0	0	0
Rattus rattus	0	1	0	0	0	0	0	0	0
Mus musculus	0	0	0	0	0	0	0	0	0
Dasyprocta leporina	0	0	0	0	0	0	0	1	1
Microcebus murinus	1	1	0	0	1	0	0	0	1
Sapajus apella	1	1	1	0	0	0	0	0	0

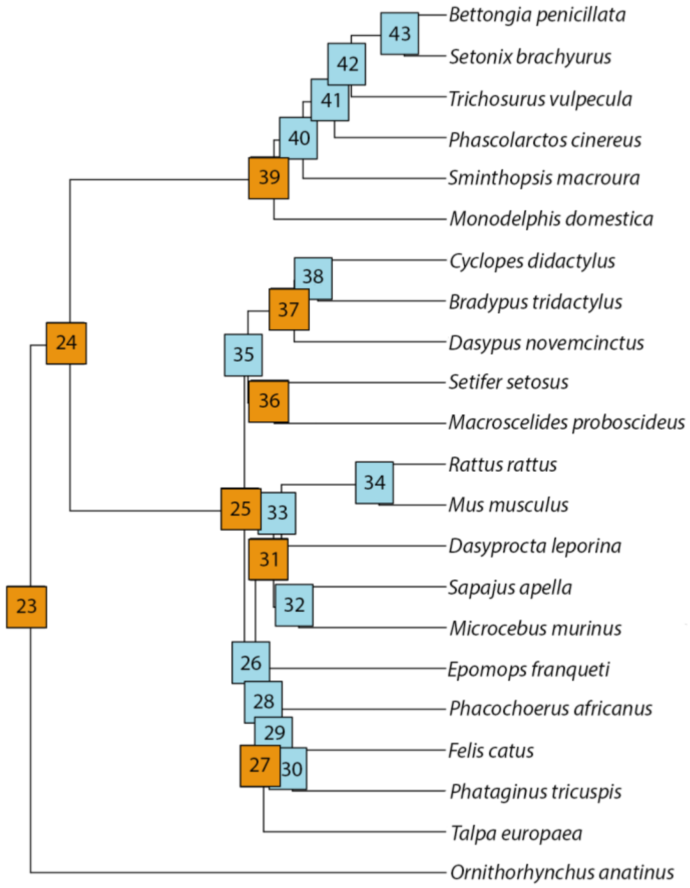

Figure S1. Time-calibrated maximum clade credibility phylogeny trimmed from the 5911 species in Upham *et al.* (2019) to the 22 species analysed here. Numbers reflect the ancestral nodes, orange nodes were the key nodes of interest, where 23 = ancestral mammal; 24 = ancestral therian; 25 = ancestral eutherian; 27 = ancestral Laurasiatheria; 31 = ancestral Euarchontoglires; 36 = ancestral Afrotheria; 37 = ancestral Xenarthra; 39 = ancestral Marsupialia.

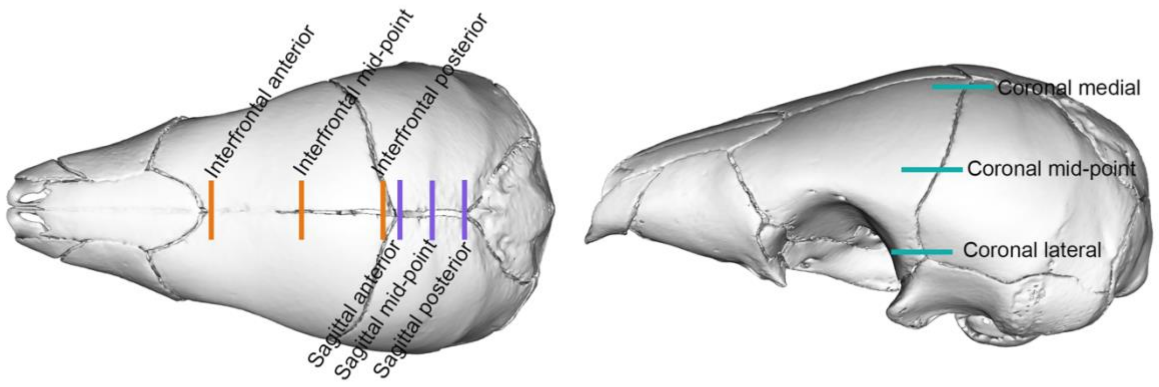

Figure S2. Location of endocranial (cross-sectional) suture fusion analysis for each of the three analysed sutures (interfrontal, sagittal, and coronal). Positions demonstrated on *Phataginus tricuspis* from the analysed dataset, in dorsal and lateral views.

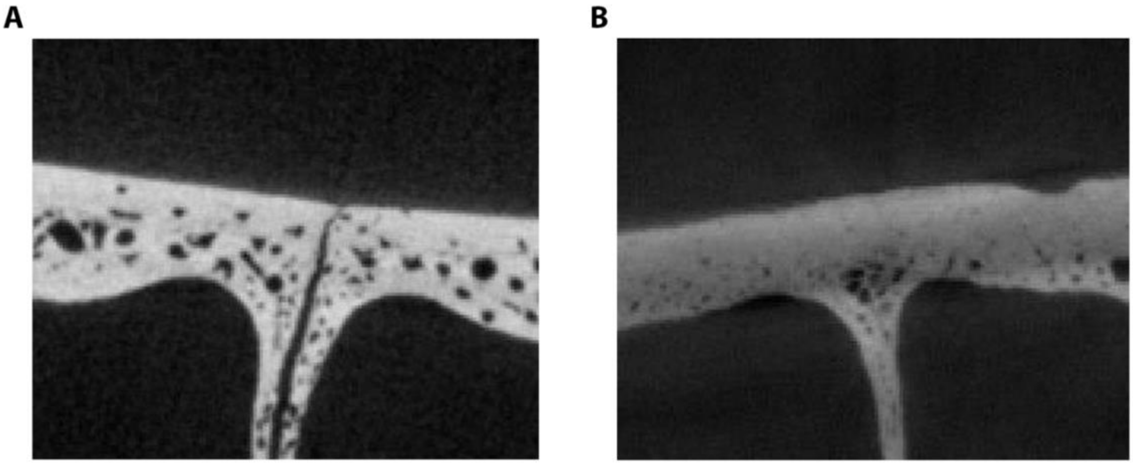

Figure S3. Cross-sectional suture fusion in micro-CT scan X-ray slices. A, an example of an open interfrontal suture; B, an example of a fused interfrontal suture. In *Felis catus* and *Phacochoerus africanus* respectively.

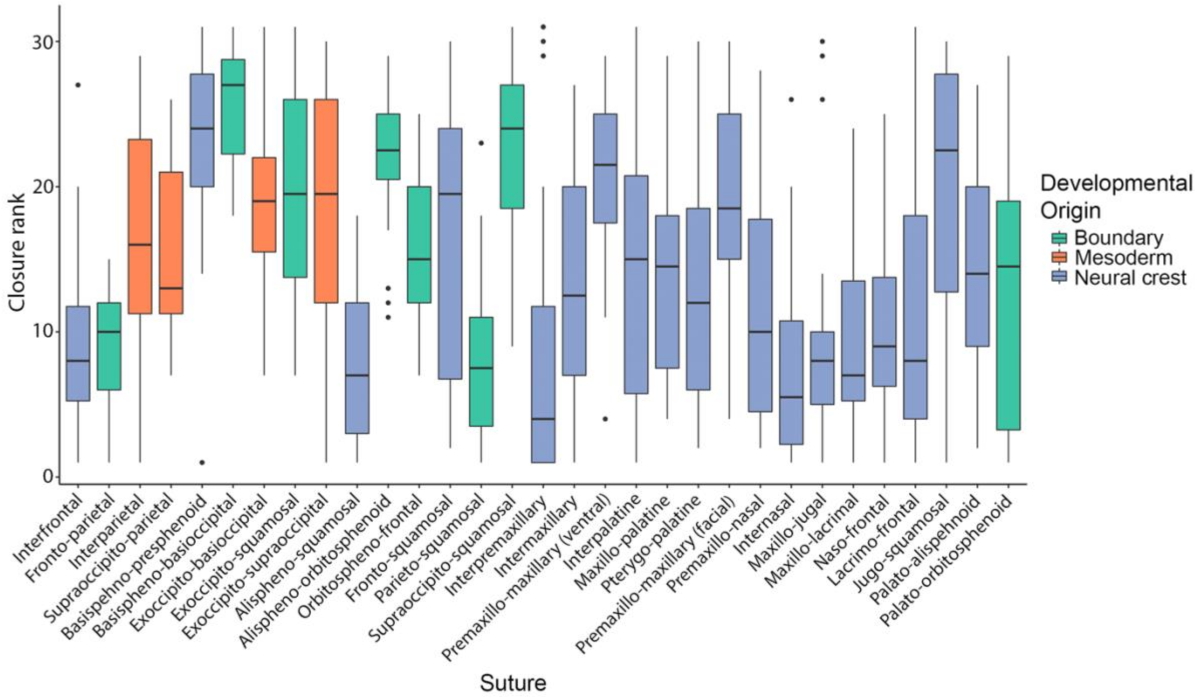

Figure S4. Distribution of closure ranks for each suture (n=31) across every specimen, identified from the ectocranial analysis. Sutures grouped by Krogman region (1930). Colours indicate the developmental origin of each suture: mesoderm, neural crest, boundary.

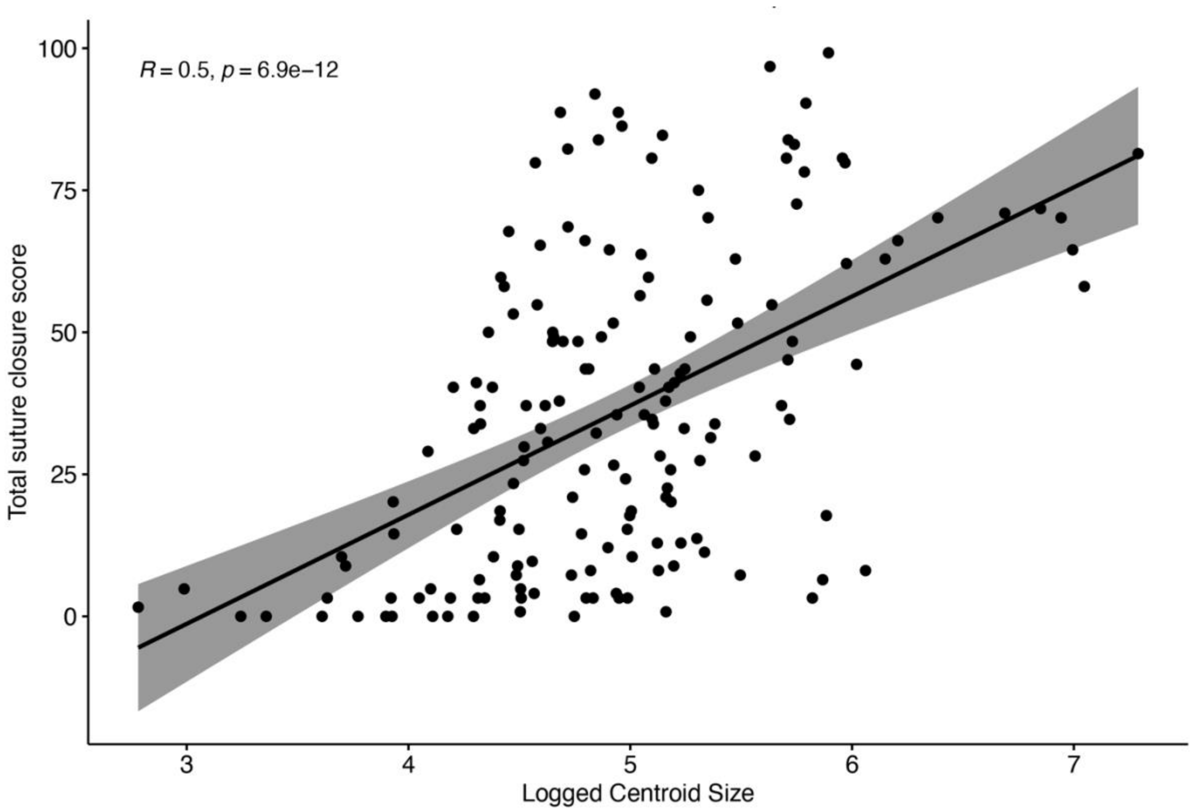

Figure S5. Spearman's rank correlation between total suture closure score from the ectocranial analysis and skull size (logged centroid size) for the entire dataset (n=165), plotted with 95% confidence interval.

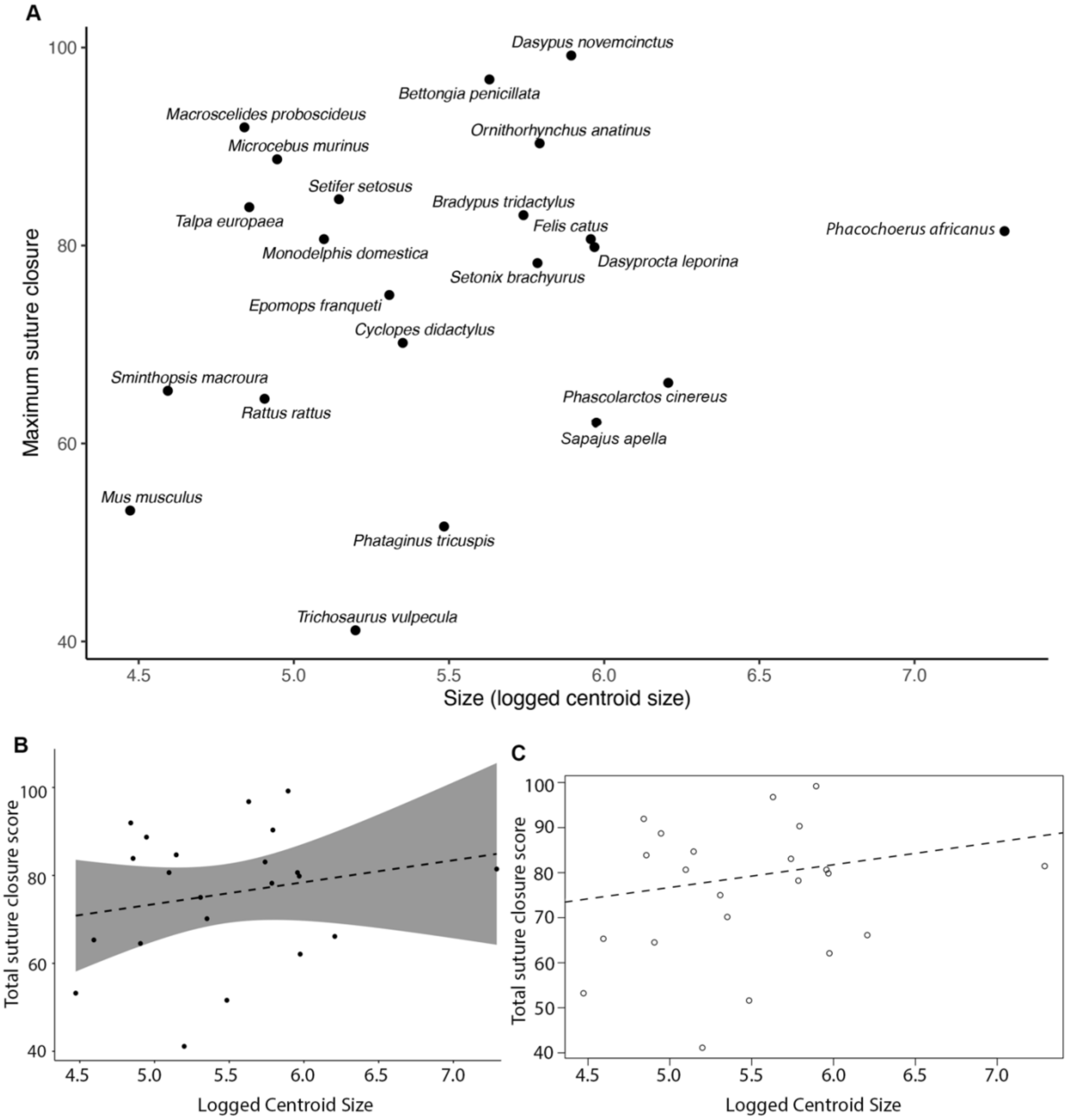

Figure S6. Correlation between total suture closure score from the ectocranial analysis and skull size (logged centroid size) for the adult only specimens (n=22). A, indicates the labelled species; B, shows the Spearman's rank correlation; C, indicates the phylogenetic generalised least squares to correct for phylogeny.

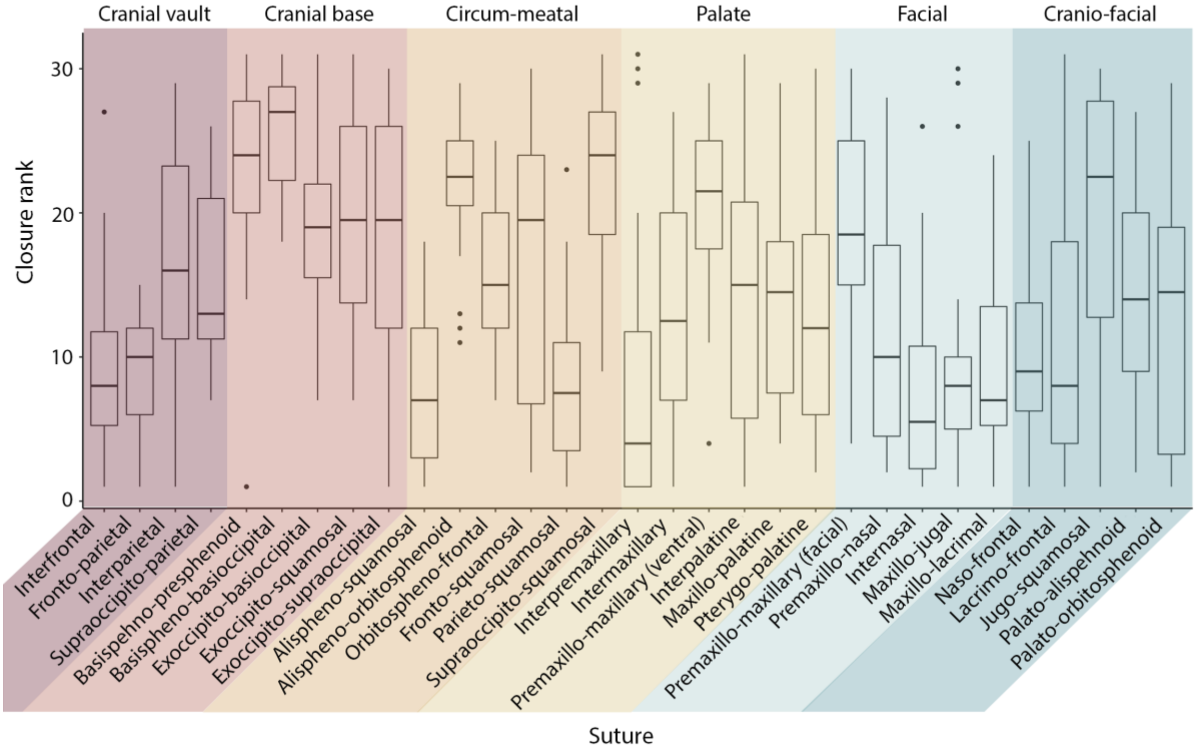

Figure S7. Distribution of closure ranks for each suture (n=31) across every specimen from the ectocranial analysis. Sutures grouped by Krogman region (1930).

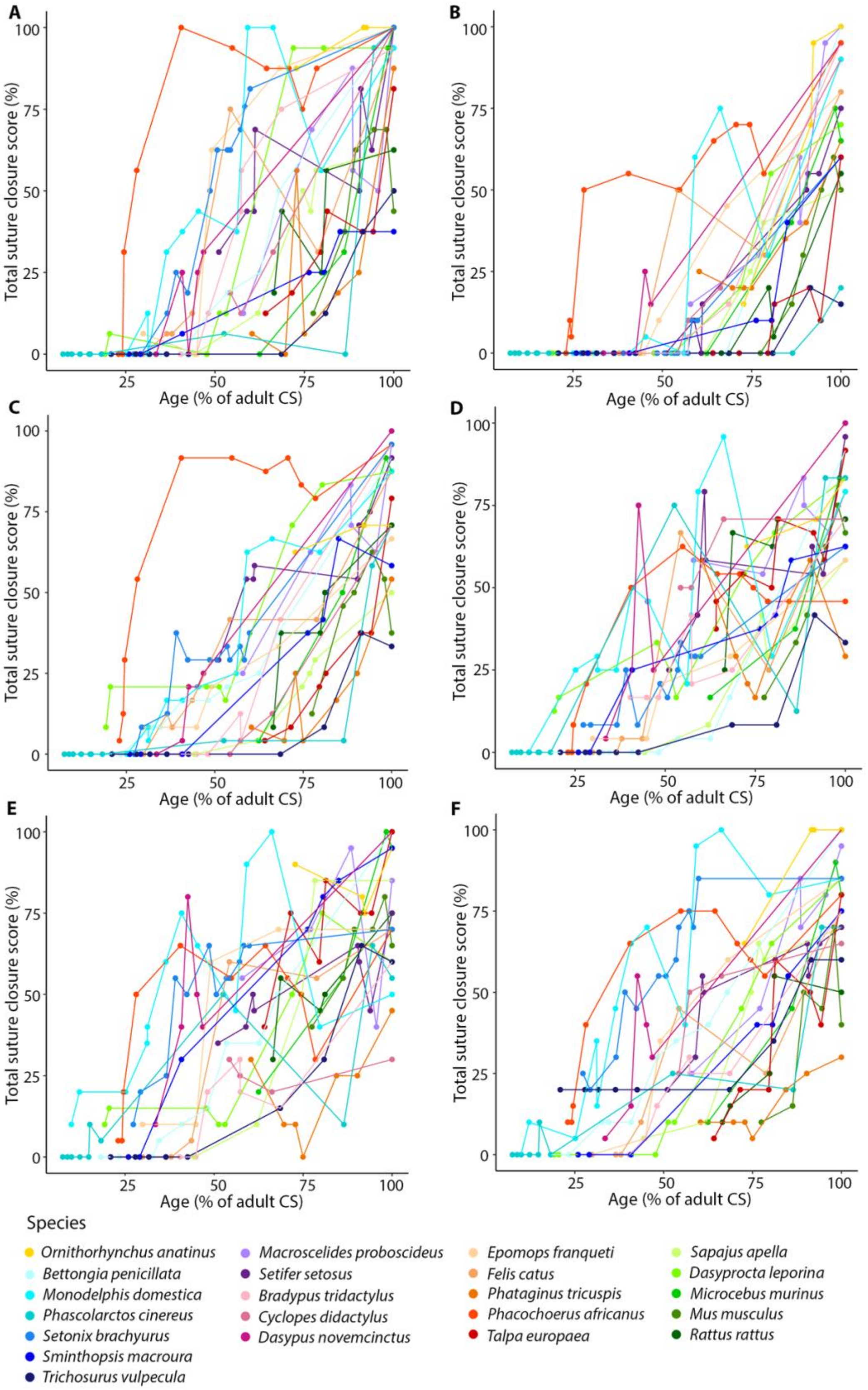

Figure S8. Change in total suture closure scores from the ectocranial analysis across ontogeny separated into the six Krogman regions: A, cranial vault; B cranial base; C circum-meatal; D palate; E facial; F cranio-facial. Ontogeny is reflected as a continuous variable, calculated as the percentage of adult centroid size, where the adult is 100%. Colours indicate the species analysed (n=22).

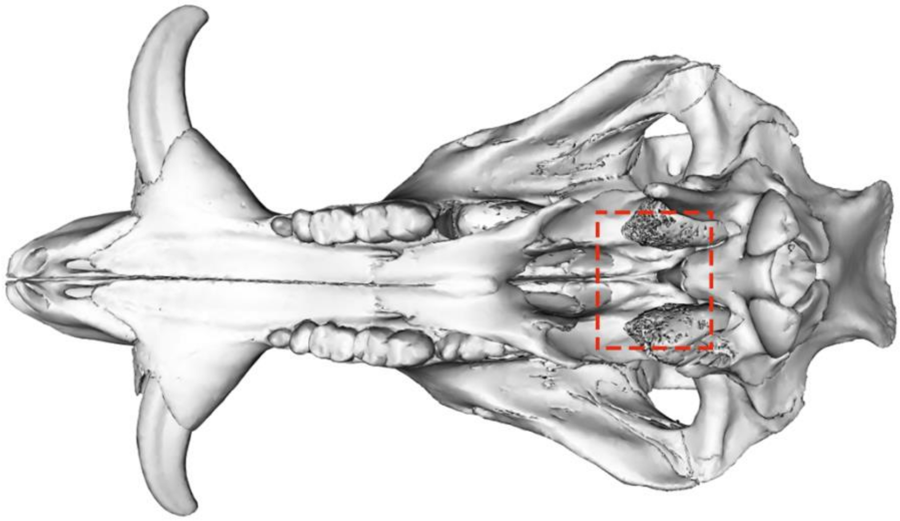

Figure S9. Cranial base flexion in *Phacochoerus africanus* skull, ventral view, with the red box indicating the region of cranial base flexion.

REFERNCES

Asher RJ, Olbricht G. 2009. Dental ontogeny in *Macroscelides proboscideus* (Afrotheria) and *Erinaceus europaeus* (Lipotyphla). Journal of Mammalian Evolution **16**: 99-115

Bailleul AM, Horner JR. 2016. Comparative histology of some craniofacial sutures and skull-base synchondroses in non-avian dinosaurs and their extant phylogenetic bracket. *Journal of Anatomy* **229**: 252-285

Bärmann EV, Sánchez-Villagra MR. 2012. A phylogenetic study of late growth events in a mammalian evoutionary radiation - the cranial sutures of terrestrial cetartiodactyl mammals. *Journal of Mammalian Evolution* **19**: 43-56

Butler RJ, Fernandez V, Nesbitt SJ, Leite JV, Gower DJ. 2022. A new pseudosuchian archosaur, *Mambawakale ruhuhu* gen. et sp. nov., from the Middle Triassic Manda Beds of Tanzania. *Royal Society Open Science* **9**: 211622

Chai Y, Jiang X, Ito Y, Bringas Jr P, Han J, Rowitch DH, Soriano P, McMahon AP, Sucov HM. 2000. Fate of the mammalian cranial neural crest during tooth and mandibular morphogenesis. *Development* **127**: 1671-1679

Coombs EJ, Felice RN, Clavel J, Park T, Bennion RF, Churchill M, Geisler JH, Beatty B, Goswami A. 2022. The tempo of cetacean cranial evolution. *Current Biology* **32**: 2233-2247.e4

Goswami A, Foley L, Weisbecker V. 2013. Patterns and implications of extensive heterochrony in carnivoran cranial suture closure. *Journal of Evolutionary Biology* **26**: 1294-1306

Goswami A, Noirault E, Coombs EJ, Clavel J, Fabre A-C, Halliday TJD, Churchill M, Curtis A, Watanabe A, Simmons NB, Beatty BL, Geisler JH, Fox DL, Felice RN. 2022. Attenuated evolution of mammals through the Cenozoic. *Science* **378**: 377-383

Hautier L, Weisbecker V, Goswami A, Knight F, Kardjilov N, Asher RJ. 2011. Skeletal ossification and sequence heterochrony in xenarthran evolution. *Evolution and Development* **13**: 460-476

Herring SW, Teng S. 2000. Strain in the braincase and its sutures during function. *American Journal of Physical Anthropology* **112**: 575-593

Jiang X, Iseki S, Maxson RE, Sucov HM, Morriss-Kay GM. 2002. Tissue origins and interactions in the mammalian skull vault. *Developmental Biology* **241**: 106-116

Koyabu D, Maier W, Sánchez-Villagra MR. 2012. Paleontological and developmental evidence resolve the homology and dual embryonic origin of a mammalian skull bone, the interparietal. *Proceedings of the National Academy of Sciences* **109**: 14075-14080

Krogman WM. 1930. Studies in growth changes in the skull and face of anthropoids: ectocranial and endocranial suture closure in anthropoids and Old World apes. *American Journal of Physical Anthropology* **46**: 315-353

McBratney-Owen B, Iseki S, Bamforth SD, Olsen BR, Morriss-Kay GM. 2008. Development and tissue origins of the mammalian cranial base. *Developmental Biolgy* **322**: 121-132

Lana-Elola E, Rice R, Grigoriadis A, Rice DC. 2007. Cell fate specification during calvarial bone and suture development. *Developmental Biology* **311**: 335-346

Morriss-Kay GM. 2001. Derivation of the mammalian skull vault*. Journal of Anatomy* **199**: 143-151

Noden DM, Francis-West P. 2006. The differentiation and morphogenesis of craniofacial muscles. *Developmental Dynamics* **235**: 1194-1218

Opperman LA. 2000. Cranial sutures as intramembranous bone growth sites. *Developmental Dynamics* **219**: 472-485

Rafferty KL, Herring SW. 1999. Craniofacial sutures: morphology, growth, and in vivo masticatory strains. *Journal of Morphology* **242**: 167-179

Rager L, Hautier L, Forasiepi A, Goswami A, Sánchez-Villagra MR. 2014. Timing of cranial suture closure in placental mammals: phylogenetic patterns, intraspecific variation, and comparison with marsupials. *Journal of Morphology* **275**: 125-140

Ramírez-Chaves HE, Wroe SW, Selwood L, Hinds LA, Leigh C, Koyabu D, Kardjilov N, Weisbecker V. 2016. Mammalian development does not recapitulate suspected key transformations in the evolutionary detachment of the mammalian middle ear. *Proceedings of the Royal Society B: Biological Sciences* **283**: 20152606

Teng CS, Cavin L, Maxson Jnr RE, Sánchez-Villagra MR, Crump JG. 2019. Resolving homology in the face of shifting germ layer origins: Lessons from a major skull vault boundary. *Elife* **8**, e52814

Thomas P, Herrel A, Hardy I, Aujard F, Pouydebat E. 2016. Exploration behavior and morphology are correlated in captive gray mouse lemurs (*Microcebus murinus*). *International Journal of Primatology* **37**: 405-415

Topczewska JM, Shoela RA, Tomaszewski JP, Mirmira RB, Gosain AK. 2016. The morphogenesis of cranial sutures in zebrafish. *PLoS ONE* **11**, e0165775

Upham NS, Esselstyn JA, Jetz W. 2019. Inferring the mammal tree: Species-level sets of phylogenies for questions in ecology, evolution, and conservation. *PLoS Biology* **17**: e3000494

Urban DJ, Anthwal N, Luo Z-X, Maier JA, Sadier A, Tucker AS, Sears KE. 2017. A new developmental mechanism for the separation of the mammalian middle ear ossicles from the jaw. *Proceedings of the Royal Society B: Biological Sciences* **284**: 20162416

Wilson LAB, Sánchez-Villagra MR. 2009. Heterochrony and patterns of cranial suture closure in hystricognath rodents. *Journal of Anatomy* **214**: 339-354
